# Design, Synthesis and In Vitro Experimental Validation of Novel TRPV4 Antagonists Inspired by Labdane Diterpenes

**DOI:** 10.3390/md18100519

**Published:** 2020-10-18

**Authors:** Sarah Mazzotta, Gabriele Carullo, Aniello Schiano Moriello, Pietro Amodeo, Vincenzo Di Marzo, Margarita Vega-Holm, Rosa Maria Vitale, Francesca Aiello, Antonella Brizzi, Luciano De Petrocellis

**Affiliations:** 1 Department of Pharmacy, Health and Nutritional Sciences, DoE 2018–2022, University of Calabria, Edificio Polifunzionale, 87036 Rende (CS), Italy; sarmaz1@alum.us.es (S.M.); gabriele.carullo@unisi.it (G.C.); 2 Department of Organic and Medicinal Chemistry, Faculty of Pharmacy, University of Seville, Profesor García González 2, 41071 Seville, Spain; mvegaholm@us.es; 3 Department of Biotechnology, Chemistry and Pharmacy, DoE 2018–2022, University of Siena, Via Aldo Moro 2, 53100 Siena, Italy; brizzi3@unisi.it; 4 Endocannabinoid Research Group (ERG), Institute of Biomolecular Chemistry, National Research Council (ICB-CNR), Via Campi Flegrei 34, 80078 Pozzuoli (NA), Italy; aniello.schianomoriello@icb.cnr.it (A.S.M.); vincenzo.dimarzo@criucpq.ulaval.ca (V.D.M.); luciano.depetrocellis@icb.cnr.it (L.D.P.); 5 Epitech Group SpA, 35030 Saccolongo (PD), Italy; 6 Institute of Biomolecular Chemistry, National Research Council (ICB-CNR), Via Campi Flegrei 34, 80078 Pozzuoli (NA), Italy; pamodeo@icb.cnr.it; 7 Canada Excellence Research Chair on the Microbiome-Endocannabinoidome Axis in Metabolic Health (CERC-MEND)-Université Laval, Québec City, QC G1V 0A6, Canada

**Keywords:** labdane scaffold, bioactive diterpenes, sclareolide, structure-activity relationships, TRPV4 channel, amides/esters, COVID-19, SARS-CoV-2

## Abstract

Labdane diterpenes are widespread classes of natural compounds present in variety of marine and terrestrial organisms and plants. Many of them represents “natural libraries” of compounds with interesting biological activities due to differently functionalized drimane nucleus exploitable for potential pharmacological applications. The transient receptor potential channel subfamily V member 4 (TRPV4) channel has recently emerged as a pharmacological target for several respiratory diseases, including the severe acute respiratory syndrome coronavirus 2 (SARS-CoV-2) infection. Inspired by the labdane-like bicyclic core, a series of homodrimane-derived esters and amides was designed and synthesized by modifying the flexible tail in position 1 of (+)-sclareolide, an oxidized derivative of the bioactive labdane-type diterpene sclareol. The potency and selectivity towards rTRPV4 and hTRPV1 receptors were assessed by calcium influx cellular assays. Molecular determinants critical for eliciting TRPV4 antagonism were identified by structure-activity relationships. Among the selective TRPV4 antagonists identified, compound **6** was the most active with an IC_50_ of 5.3 μM. This study represents the first report of semisynthetic homodrimane TRPV4 antagonists, selective over TRPV1, and potentially useful as pharmacological tools for the development of novel TRPV4 channel modulators.

## 1. Introduction

Labdanes are bicyclic diterpenes found as secondary metabolites in marine organisms and in plants, characterized by a high chemical diversity. They exhibit a broad panel of pharmacological activities, ranging from antimicrobial and anti-inflammatory to cytotoxic and antitumoral ones [1].

The diversity of organisms, nature, extent and distribution of chemical modifications and functionalization and the corresponding spread of biological functions make the labdane diterpenes and their derivatives a set of naturally occurring libraries of bioactive compounds [2].

In the field of drug discovery, natural compounds represent useful tools for the development of transient receptor potential (TRP) channel modulators [3,4].

In particular, TRPV4 is a polymodal, non-selective cation channel, belonging to the vanilloid subfamily (V) member 4 of the TRP ion channels [5,6,7]. It is activated by a series of physical and chemical stimuli, including temperature, pH, hypotonicity, and stretch, as well as arachidonic acid and its metabolites. TRPV4 is a homo-tetramer sharing an overall architecture similar to that of other TRPV family members, featuring a transmembrane domain (TMD), consisting of helices S1 to S6, and a cytosolic region formed by the N- and C-terminal domains. The transmembrane helices S5–S6 and the pore loop form the pore channel, flanked by an S1–S4 voltage-sensor like domain (VSLD) showing a peculiar arrangement as emerged from the recently solved structures of TRPV4 from *Xenopus tropicalis* [8].

TRPV4 is implicated in various physiological processes due to it high expression in various tissues of the human body [8]. In particular, it is expressed in alveolo-capillary and immune cells of the immune system, such as alveolar macrophages and neutrophil granulocytes, which contribute to alveolo-capillary barrier function through proteases and cytokine release, as well as reactive oxygen species production [9].

TRPV4 has recently emerged as a pharmacological target for the treatment of pulmonary oedema caused by COVID-19 (coronavirus disease of 2019). TRPV4-evoked calcium uptake in lung endothelium has been associated with elevated pulmonary vascular pressure, lung congestion, and resulting dyspnea. Selective TRPV4 agonists have been shown to increase lung permeability in a dose-dependent manner in wild-type mice but not in TRPV4 knockout mice, suggesting the advantage of TRPV4 inhibition in lung oedema treatment [10].

**Figure 1:**
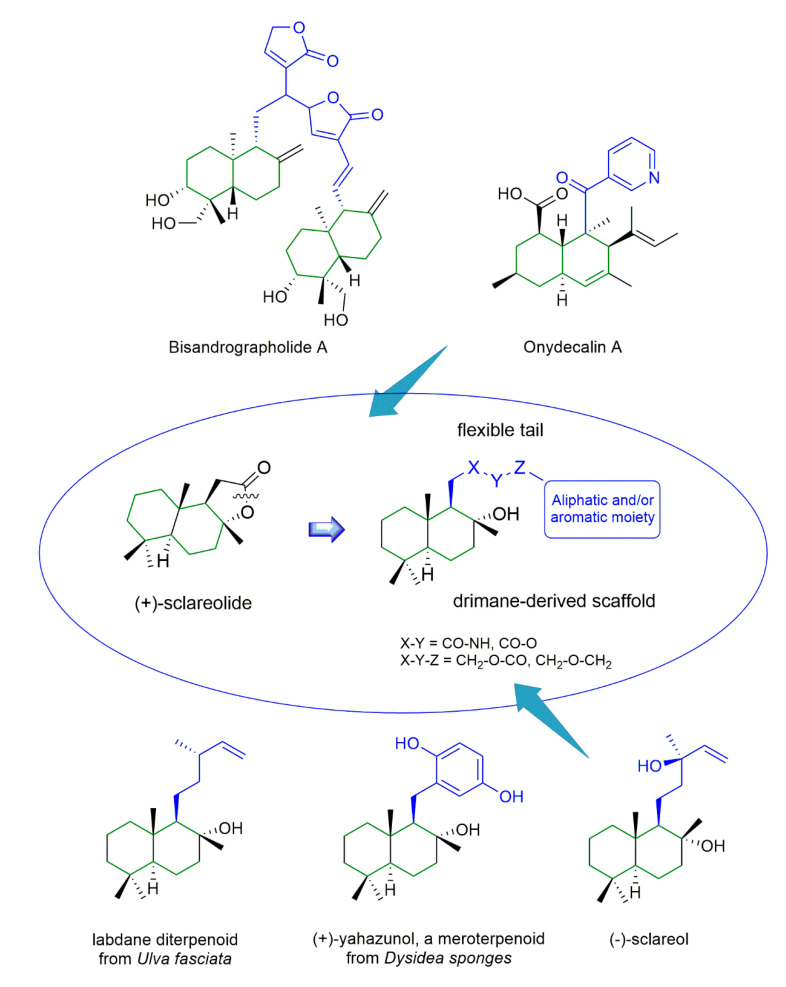
Design of new drimane-derived antagonists inspired by labdane diterpenoids starting from known natural TRPV4 ligands.

To date, only a limited number of TRPV4 modulators have been identified; thus, the discovery and the development of new selective TRPV4 ligands represent an attractive challenge [11,12]. The first identified TRPV4 agonist was bisandrographolide A (BAA, EC_50_ 790–950 nM, Figure 1), a plant dimeric diterpenoid [13]. Among the antagonists, the quinoline-carboxamide GSK2193874, as well as 1-(4-piperidinyl)-benzimidazole amides [14], were developed for the treatment of pulmonary oedema associated with congestive heart failure [15].

The pyridine polyketide onydecalin A (Figure 1) was also validated as a TRPV4 antagonist (IC_50_ 45.9 µM), with a partial activity towards another TRPV channel, i.e., member 1 (TRPV1) [16].

The occurrence of a *trans*-decalin lipophilic moiety in two plant-derived TRPV4 modulators, i.e., bisandrographolide A, an agonist, and onydecalin A, an antagonist, as well as in labdane terpenes, such as (+)-yahazunol [17,18] and sclareol [19] (Figure 1), prompted us to exploit the potential of the homodrimane bicyclic nucleus for the development of a new class of semi-synthetic TRPV4 modulators.

Interestingly, the flexible tail of labdanescaffold is differently functionalized in natural compounds or even replaced by different functional group families, pointing to a suitable region to be varied for the creation of targeted libraries toward specific classes of pharmacologically interesting proteins.

In the present study, we focused on (+)-sclareolide (Figure 1), a fragrant compound found in *Salvia sclarea*, used as flavor additive in food. It represents the oxidized derivative of the bioactive labdane-type diterpene sclareol. (+)-Sclareolide gained attention due to its many biomedical properties, including anticancer and antiviral activities [20,21,22]. In addition, it also displays chemical versatility, since its lactone ring condensed with a *trans*-decalin-related homodrimane core can be easily opened and functionalized [23].

We performed the synthesis of a small, but diversified library of new derivatives, characterized by the homodrimane backbone bearing flexible tails of different nature and chemical properties at position 1 (Figure 1). We used the commercially available (+)-sclareolide as starting molecule. In particular, the substituent groups, bound to the bicyclic nucleus by either an amide or ester or ether functionality, differ in size, flexibility, and electronic properties.

Therefore, recurring chemical motifs, such as variously substituted benzyl and phenylethyl residues, were inserted into the new molecules by proper choices of amines and alcohols. Functional properties and selectivity on TRPV4 and TRPV1 channels of all final compounds were then evaluated by an in vitro calcium influx assay in intact living cells overexpressing either channel.

Among these new derivatives, some relative potent and selective TRPV4 antagonists were identified and their structure-activity relationship (SAR) study highlighted the crucial role of specific functional groups in eliciting TRPV4 antagonism that could be used for the development of a new class of selective TRPV4 modulators.

## 2. Results

### 2.1. Chemistry

**Scheme 1:**
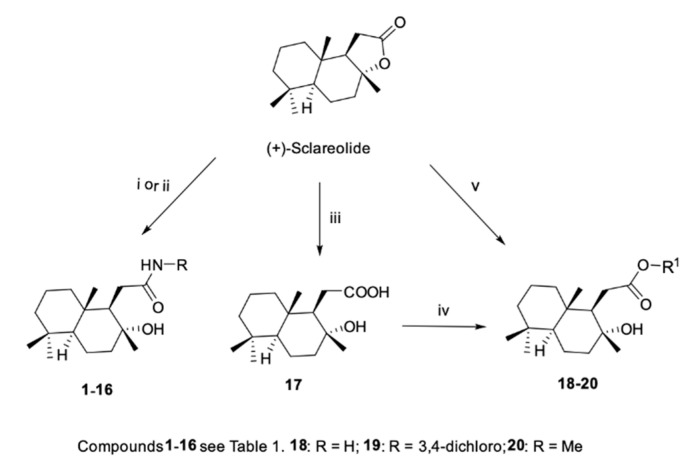
Reagents and conditions. (**i**) Aromatic amine, DIBAL-H, dry THF, rt (room temperature), 3–5 h; (**ii**) Aliphatic amine, dry THF, 45 ºC, 48 h; (**iii**) NaOH, MeOH, rt, 2 h; (**iv**) R1-Br, K_2_CO_3_, dry DMF, rt, 24 h; (**v**) MeOH, 45 °C, 72 h.

**Scheme 2:**
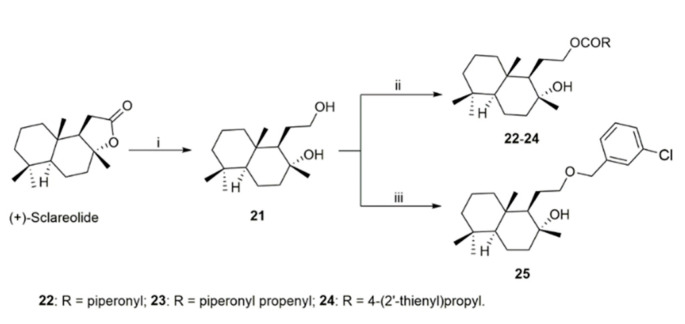
Reagents and conditions. (**i**) LiAlH_4_, dry THF, rt, 6 h; (**ii**) RCOOH, EDCI, DMAP, DCM, rt, 48–72 h; (**iii**) NaH, 3-chlorobenzyl bromide, dry THF, reflux, 48 h.

With the aim of synthesizing a set of new compounds, we introduced several modifications in the (+)-sclareolide scaffold by lactone ring-opening, thus resulting in the homodrimane sesquiterpene moiety. Amide derivatives **1**–**16** were generated through two different procedures both carried out in THF (tetrahydrofuran) as the solvent, as depicted in Scheme 1. Derivatives **1**–**3** were obtained by DIBAL-H (diisobutylaluminium hydride) assisted amidation using aromatic amines while the direct aminolysis of (+)-sclareolide using aliphatic amines provided instead compounds **4**–**16**. Homodrimanyl acid esters **18** and **19** were prepared in two steps (Scheme 1).

(+)-Sclareolide was first hydrolyzed with sodium hydroxide in methanol to give the free carboxylic acid **17** which was then reacted in anhydrous DMF (dimethylformamide) with the appropriate benzyl bromide in the presence of K_2_CO_3_, thus affording the desired compounds. Methyl ester **20** was obtained by alcoholysis, following a procedure previously described (see Supplementary Materials). Diol derivative **21** represented the key intermediate for the synthesis of reverted esters **22**–**24** and ether **25**, as shown in Scheme 2. Reduction of the carbonyl functionality of (+)-sclareolide with LiAlH_4_ in anhydrous THF provided the desired diol **21** (in good yield), which was converted into the corresponding homodrimanyl alcohol esters **22**–**24** by reaction with the appropriate carboxylic acids under Steglich conditions. Differently, ether **25** was synthesized by reacting the diol derivative **21** with 3-chlorobenzyl bromide under basic condition in refluxing anhydrous THF.

### 2.2. TRPV4 Assay

The interaction properties of all homodrimane-derived compounds **1**–**16**, **18**–**19**, and **22**–**25** with either TRPV type-4 or type-1 were studied in human embryonic kidney (HEK)-293 cells overexpressing either the rat recombinant type-4 (rTRPV4), which shares with the human orthologue ~95% of sequence identity, and the human recombinant type-1 (hTRPV1) channel, evaluating the changes in intracellular calcium levels. The values for both EC_50_ (for activation) and IC_50_ (for antagonism) were calculated and are summarized in Table 1.

All compounds share the homodrimane scaffold while they differ in the nature of the substituent at position 1. This latter includes an aliphatic and/or aromatic moiety, connected to C1 by a spacer of variable length containing one of the following functional groups: amide, ester, reverted ester, and ether. Accordingly, the final set of synthesized compounds featured homodrimanyl acid amides (compounds **1**–**16**), homodrimanyl acid esters (compounds **18** and **19**), homodrimanyl alcohol esters (compounds **22**–*24*), having a reverted ester group, and one ether derivative (compound **25**). Besides this library of trans-decalin-related homodrimane derivatives, also (+)-sclareolide, the methyl ester **20**, and homodrimanyl alcohol **21**, a key intermediate in the synthesis of the reverted ester and ether compounds (see Scheme 1), were tested for their ability to interact with rTRPV4 and hTRPV1. Homodrimanyl acid methyl ester **20**, reverted esters **22**–**24**, and ether compound **25** were found to be inactive toward both hTRPV1 and rTRPV4, regardless of the size, electronic properties or spacer length of the substituent at position 1 (see Table 1). Similarly, the natural starting compound, (+)-sclareolide, and compound **21**, characterized by the 2-hydroxyethyl group at position 1, were unable to modulate either channel. Instead, the homodrimanyl acid amide series **1**–**16** is by far more interesting, since most derivatives behaved as rTRPV4 ligands endowed with antagonistic activity. Moreover, all amides resulted completely selective for rTRPV4, showing no relevant activity versus hTRPV1 (see Supplementary Materials).

### 2.3. Cytotoxicity Assay

**Figure 2:**
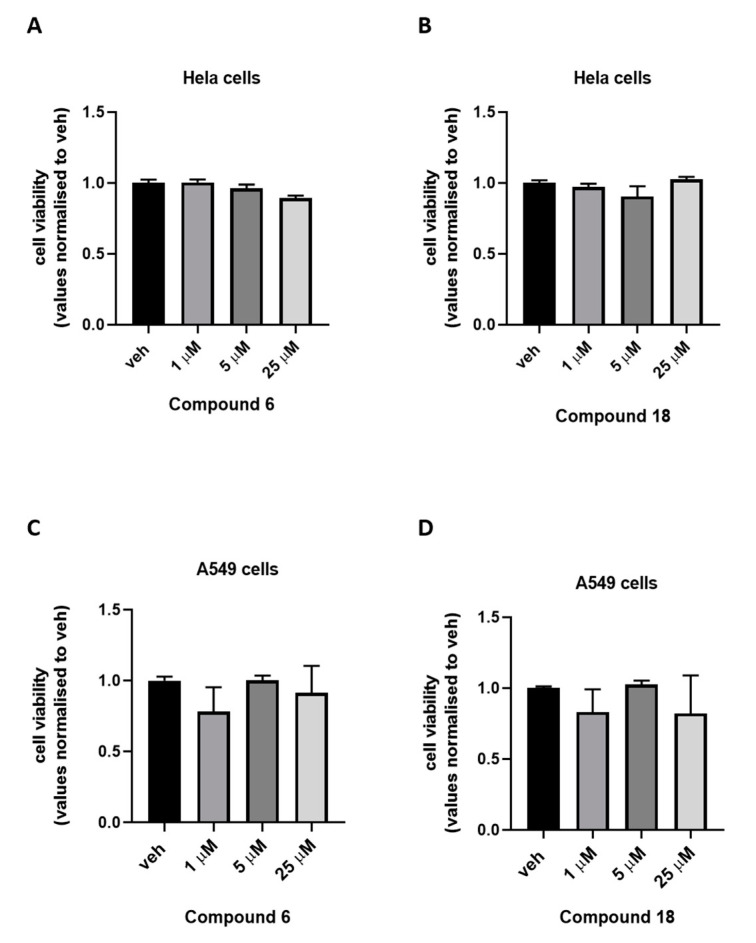
Cell viability measured in HeLa and A549 cells treated for 24 h with increasing concentration of compound **6** (panel **A**, **C**) and **18** (panel **B**, **D**). Bar graphs show cell viability measured using the 3-(4,5-dimethylthiazol-2-yl)-2,5-diphenyltetrazolium bromide (MTT) assay. Data are expressed as optical density (OD) at 595 nm, normalized to control (vehicle). The bar is the standard error of the mean (SEM) on 3 independent determinations. Statistically significant differences were accepted when the *p*-value was at least ≤0.05.

To exclude cytotoxic effects, the most active compounds within the series, namely **6** and **18**, were evaluated on both human cervical (HeLa) and human lung (A549) carcinoma cells at 1, 5, and 25 μM, using the 3-(4,5-dimethylthiazol-2-yl)-2,5-diphenyltetrazolium bromide (MTT) assay. As shown in Figure 2, neither compound caused statistically-significant cytotoxic effects at any tested concentration.

## 3. Discussion

### Biological Activity and Structure-Activity Relationships (SARs)

Among the aromatic amides, characterized by a three-atom spacer and a phenyl head, only compound **3** behaved as a good rTRPV4 agonist, even though not very effective. This result suggests that a relatively bulky and electron-rich group (i.e., 2-(1H-pyrrol-1-yl), compound **3**), rather than a small and strongly electronegative substituent (i.e., 2-fluoro, compound **2**), is necessary at position 2 of the phenyl ring to produce rTRPV4 modulation. Amides characterized by a benzyl- or phenylethyl- moiety provided the most notable compounds, all behaving as rTRPV4 modulators with the only exception of compound **15**. Indeed, in the benzyl amide subset of compounds, a substituent in meta or para position was generally well tolerated. However, electron-attracting halogens (compounds **5**, **6** and **7**) performed better than an electron-donor methoxy group (compounds **8** and **9**); moreover, the 4-chloro derivative (compound **5**) was more active than the corresponding 4-fluoro one (compound **7**). This effect can be probably ascribed to composite factors including relative halogen-bonding propensity, lipophilicity and electron withdrawing properties. The introduction of a second chlorine at position 3 (compound **6**), further increased the activity, producing the most potent antagonist in this library of trans-decalin-related homodrimane derivatives. Conversely, the introduction of a bulky substituent, such as a second phenyl ring in para position (compound **15**) or the replacement of the benzyl- with a furan-2-yl-methyl- (compound **14**) moiety, was detrimental for rTRPV4 interaction, leading to derivatives either completely devoid of activity, i.e., compound **15**, or with very weak antagonist properties (compound **14**). Concerning the phenylethyl amide subset, compounds **11**, **12**, and **13** were more potent than compound **10**. Thus, a halogen atom in para- or meta-position of the phenyl nucleus caused a slight increase of the activity, consistently with previous observations. Anyway, these derivatives appeared to be less affected by the nature of the halogen atom, as indicated by their IC_50_ value trend (compounds **11**–**13**). Considering the influence of the length of the spacer connecting the homodrimane portion to the amide head, compounds **10**, **11**, and **12** performed better than compounds **4**, **5**, and **7**. In this view, experimental results suggested that a five-atom spacer, typical of the phenylethyl derivatives, provided a higher potency than the four-atom spacer present in the benzylic ones. However, a further extension to a six-atom spacer, as in compound **16**, characterized by a 3-(1H-imidazol-1-yl)propyl moiety, determined the complete loss of activity on rTRPV4. Since the amide series gave rise to the most interesting compounds within the tested panel, the role of the amide group was explored by synthesizing two benzyl analogs bearing the ester group in place of the amide, i.e., homodrimanyl acid esters **18** and **19**. Intriguingly, the biological activity results were quite surprising and not conclusive. Indeed, compound **18**, the benzyl ester analog of amide **4**, resulted in a considerably more potent antagonist (IC_50_ 5.41 µM and 32.0 µM, respectively), representing the most active synthesized derivative together with amide **6** (IC_50_ 5.30 μM). On the contrary, compound **19**, the 3,4-dichlorobenzyl ester analog of amide **6**, was completely devoid of activity on rTRPV4. A possible explanation for these results could be that the homodrimanyl amide and ester derivatives might occupy different receptor (sub)sites. The higher flexibility of the ester group compared to the amide group and the lack of the H-bond donor could facilitate its accommodation in a narrow pocket, filled by the phenyl group. The presence of two chlorine substituents, by increasing the bulkiness of the aromatic ring, may give rise to steric bumps within the pocket, thus preventing a correct ligand orientation. Finally, the presence of a carbonyl functionality (and its position in the spacer), appeared as stringent requirements, too, since its displacement, as in reverted esters **22**–**24**, or its removal, as in the ether derivative **25**, all resulted in inactive derivatives. Unfortunately, the combination of low resolution/missing regions of the available TRPV4 3D structures with the lack of structural insights in the binding site of known antagonists, do not allow at the present any reliable prediction and unequivocal identification of the putative binding sites of these compounds.

## 4. Materials and Methods

### 4.1. Chemicals, Materials, and Methods

All the reagents were purchased from Merck (Darmstadt, Germany) or Alfa Aesar (Tewksbury, MA, USA) and were used as received. Melting points were obtained using a Gallenkamp (G) (Fiorano Modenese, Italy) melting point apparatus. The structures of final compounds were unambiguously assessed by ^1^H NMR (nuclear magnetic resonance) and ^13^C NMR. Spectra were recorded in the indicated solvent (Chloroform- CDCl_3_, Dimethyl Sulphoxide-DMSO-*d_6_*) at 25 °C on a Bruker 300 MHz spectrometer (Bruker, Milano, Italy) or a Bruker Advance DPX400 employing TMS (tetramethyl silane)as internal standard. Chemical shifts are expressed in δ values (ppm) and coupling constants (*J*) in hertz (Hz). IR spectra were recorded on a PerkinElmer machine 10.4.00 (PerkinElmer, Milan, Italy). Reactions were monitored by TLC (thin layer chromatography) on silica gel plates Merck 60 F254 (Merck, Burlington, MA, USA). Final products were purified by a flash chromatography system with column chromatography, using Merck 60 silica gel, 230–400 mesh. Elemental analyses were performed on Leco Trunspec CHNS Micro elemental system (St. Joseph, MI, USA). The purity of final compounds was evaluated by C, H, N analysis, and it was confirmed to be ≥95%.

### 4.2. Chemistry

#### 4.2.1. General Procedure for the Synthesis of Homodrimanyl Aryl Amides (**1**–**3**): DIBAL-H-Mediated Amidation From Anilines

According with Li D. et al. (2017) [24], a solution of DIBAL-H (1 M in toluene, 3.0 eq.) was added dropwise to a solution of appropriate aniline (1.0 mmoL, 2.5 eq.) in anhydrous THF (1.5 mL) at 0 °C, under argon flux and stirring. The reaction mixture was warmed to rt and stirred for the next 2 h. The prepared complex was used directly for the aminolysis. (+)-Sclareolide (100 mg, 0.4 mmoL, 1.0 eq.) was dissolved in anhydrous THF (1.0 mL), and the DIBAL-H-aniline complex solution was added. The mixture was stirred at rt until sclareolide spot disappeared on the TLC plate (about 3–5 h). Then, it was cooled to 0 °C, quenched with a 1 M KHSO_4_ aqueous solution (2.0 mL), and extracted with DCM (3 × 10 mL). The combined organic layers were finally washed with brine and dried over anhydrous Na_2_SO_4_. After filtration, the evaporation of the solvent to dryness furnished the corresponding homodrimanyl amide. Column chromatography using a mixture of petroleum eter/ethyl acetate as eluent gave the pure compound in good yield.

##### 2-((1*R*,2*R*,4a*S*,8a*S*)-2-Hydroxy-2,5,5,8a-tetramethyldecahydronaphthalen-1-yl)-*N*-phenylacetamide (**1**)

Ref. [21] The product was purified by column chromatography using PE/EtOAc (5/1) as eluent to give the compound as a white solid, mp 166–167 °C (G). Yield 84%. ^1^H NMR (300 MHz, CDCl_3_) δ (ppm): 8.77 (s, 1H, NH), 7.45 (d, *J* = 7.5 Hz, 2H, Ar), 7.21 (t, *J* = 7.4 Hz, 2H, Ar), 7.01 (t, *J* = 7.4 Hz, 1H, Ar), 3.10 (s, 1H, OH), 2.55 (dd, *J*_1_ = 15.3 Hz, *J*_2_ = 4.3 Hz, 1H, CH_2_CO), 2.22 (dd, *J*_1_ = 15.5 Hz, *J*_2_ = 4.7 Hz, 1H, CH_2_CO), 1.89 (dt, *J*_1_ = 12.3 Hz, *J*_2_ = 2.8 Hz, 1H), 1.77 (t, *J* = 4.1 Hz, 1H), 1.70-1.18 (m, 9H), 1.12 (s, 3H, CH_3_), 1.10-0.85 (m, 1H, CH), 0.80 (s, 3H, CH_3_), 0.74 (s, 6H, CH_3_). ^13^C NMR (75 MHz, CDCl_3_) δ (ppm): 174.0, 138.4, 129.8, 127.7, 124.8, 120.7, 118.8, 73.6, 57.8, 55.1, 43.4, 41.7, 38.8, 35.9, 34.9, 33.2, 24.8, 23.3, 22.2, 20.5, 18.2, 143.6. Anal. Calcd. for C_22_H_33_NO_2_: C, 76.92; H, 9.68; N, 4.08. Found: C, 77.05; H, 9.71; N, 4.07.

##### *N*-(2,5-difluorophenyl)-2-((1*R*,2*R*,4a*S*,8a*S*)-2-hydroxy-2,5,5,8a-tetramethyldecahydronaphthalen-1-yl)acetamide (**2**)

The product was purified by column chromatography using PE/EtOAc (8/1) as eluent to give the compound as a white solid, mp 165–166 °C. IR ν (cm^−1^): 3266, 2925, 1680, 1630, 1542, 1441, 1189, 754. Yield 73%. ^1^H NMR (400 MHz, CDCl_3_) δ (ppm): 8.78 (brs, 2H), 8.24-8.14 (m, 1H, Ar), 6.99-6.91 (m, 1H, Ar), 6.67-6.61 (m, 1H, Ar), 2.59 (dd, *J*_1_ = 15.0 Hz, *J*_2_ = 4.6 Hz, 1H, CH_2_CO), 2.26 (dd, *J*_1_ = 15.0 Hz, *J*_2_ = 4.0 Hz, 1H, CH_2_CO), 1.92 (dt, *J*_1_ = 12.3 Hz, *J*_2_ = 3.1 Hz, 1H, -CH_2_-COH(CH_3_)), 1.78 (t, *J* = 4.2 Hz, 1H, -CH-COH(CH_3_)), 1.71-1.66 (m, 2H), 1.60-1.50 (m, 2H), 1.48-1.24 (m, 4H), 1.20 (s, 3H, COH(CH_3_)), 1.00-0.89 (m, 2H), 0.85 (s, 3H, CH_3_), 0.79 (s, 3H, CH_3_), 0.78 (s, 3H, CH_3_). ^13^C NMR (100 MHz, CDCl_3_) δ (ppm): 173.6 (C = O), 158.7 (d, *J* = 240 Hz, Cq-F), 148.3 (d, *J* = 238 Hz, Cq-F), 128.1 (CqAr), 114.9 (dd, *J*_1_ = 9.7 Hz, *J*_2_ = 21.9 Hz, CHAr), 109.2 (d, *J* = 24.6 Hz, CHAr), 108.5 (d, *J* = 36.3 Hz, CHAr), 74.1 (Cq-OH(CH_3_)), 58.2 (CH-CH_2_CO), 56.0 (CH), 44.2, 41.8, 39.4, 38.9 (Cq-(CH_3_)2), 34.7, 33.3, 29.7, 24.3, 21.4, 20.5, 18.2, 15.3. Anal. Calcd. for C_22_H_31_F_2_NO_2_: C, 69.63; H, 8.23; N, 3.69. Found: C, 69.50; H, 8.27; N, 3.68.

##### *N*-(2-(1*H*-pyrrol-1-yl)phenyl)-2-((1*R*,2*R*,4a*S*,8a*S*)-2-hydroxy-2,5,5,8a-tetramethyldecahydronaphthalen-1-yl)acetamide (**3**)

The product was purified by column chromatography using PE/EtOAc (4.5/1) as eluent to give the compound as a light yellow oil. Yield 80%. IR ν (cm^−1^): 3669, 2970, 1681, 1525, 1451, 1215, 1069, 748, 666. ^1^H NMR (400 MHz, CDCl_3_) δ (ppm): 8.32 (d, *J* = 8.2 Hz, 1H, Ar), 8.10 (brs, 1H), 7.36 (t, *J* = 7.8 Hz, 1H, Ar), 7.21 (d, *J* = 7.4 Hz, 1H, Ar), 7.10 (d, *J* = 7.6 Hz, 1H, Ar), 6.79 (t, *J* = 1.8 Hz, 2H, Pyrrol), 6.36 (t, *J* = 1.8 Hz, 2H, Pyrrol), 2.41 (dd, *J*_1_ = 14.9 Hz, *J*_2_ = 4.5 Hz, 1H, CH_2_CO), 2.11 (dd, *J*_1_ = 14.9 Hz, *J*_2_ = 4.1 Hz, 1H, CH_2_CO), 1.84 (dt, *J*_1_ = 11.8 Hz, *J*_2_ = 2.9 Hz, 1H, -CH_2_-COH(CH_3_)), 1.69-1.63 (m, 2H), 1.61-1.48 (m, 2H), 1.45-1.33 (m, 3H), 1.28-1.10 (m, 2H), 1.05 (s, 3H, COH(CH_3_)), 0.90-0.89 (m, 2H), 0.86 (s, 3H, CH_3_) 0.76 (s, 3H, CH_3_), 0.73 (s, 3H, CH_3_). ^13^C NMR (100 MHz, CDCl_3_) δ (ppm): 173.8 (C=O), 134.8 (CqAr), 130.7 (CqAr), 129.0 (CHAr), 127.3 (CHAr), 123.7 (CHAr), 122.8 (×2, CHPyrrol), 121.8 (CHAr), 109.7 (×2, CHPyrrol), 73.4 (Cq-OH(CH_3_)), 59.3 (CH-CH_2_CO), 56.2 (CH), 43.7, 41.8, 39.4, 38.8 (Cq-(CH_3_)_2_), 34.7, 33.3, 33.2, 23.7, 21.4, 20.4, 18.2, 15.2. Anal. Calcd. for C_26_H_36_N_2_O_2_: C, 76.43; H, 8.88; N, 6.86. Found: C, 76.52; H, 8.91; N, 6.84.

#### 4.2.2. General Procedure for the Synthesis of Homodrimanyl Aliphatic Amides (**4**–**16**): Aminolysis Reaction from Amines

According to a published procedure [21] with little modifications, a solution of (+)-sclareolide (100 mg, 0.4 mmoL, 1.0 eq.) and the opportune amine in dry THF (1.5 mL) was stirred at 45 °C for 48–72 h. The reaction mixture was then concentrated under reduced pressure and dispersed in water (15 mL). The inorganic phase was extracted twice with EtOAc (15 mL), and the collected organic layers were washed with brine (15 mL), dried over anhydrous Na_2_SO_4_ and filtered. The solvent was removed under vacuum, and the crude product was purified by flash chromatography using a mixture of PE/EtOAc as eluent to give the homodrimanyl aliphatic amide in good yield.

##### *N*-benzyl-2-((1*R*,2*R*,4a*S*,8a*S*)-2-hydroxy-2,5,5,8a-tetramethyldecahydronaphthalen-1-yl)acetamide (**4**)

The product was purified by column chromatography using PE/EtOAc (1.5/1) as eluent to give the compound as a colorless oil. Yield 98%. IR ν (cm^−1^): 3014, 2926, 1650, 1214, 748, 666. ^1^H NMR (300 MHz, CDCl_3_) δ (ppm): 7.28-7.14 (m, 4H, Ar), 6.92 (t, *J* = 12.1 Hz, 1H, Ar), 4.38-4.21 (m, 2H, CH_2_NH), 3.53 (s, 1H, OH), 2.36 (dd, *J*_1_ = 15.2 Hz, *J*_2_ = 4.5 Hz, 1H, CH_2_CO), 2.09 (dd, *J*_1_ = 15.5 Hz, *J*_2_ = 4.7 Hz, 1H, CH_2_CO), 1.83 (dt, *J*_1_ = 12.1 Hz, *J*_2_ = 2.7 Hz, 1H), 1.68 (t, *J* = 4.7 Hz, 1H), 1.62-1.08 (m, 9H, CH_2_), 1.02 (s, 3H, CH_3_), 0.94-0.85 (m, 1H, CH), 0.80 (s, 3H, CH_3_), 0.71 (s, 6H, CH_3_). ^13^C NMR (75 MHz, CDCl_3_) δ (ppm): 175.6, 138.4, 129.1, 128.6, 128.3, 128.1, 72.9, 57.3, 56.6, 55.1, 43.5, 41.7, 38.8, 34.1, 33.2, 32.5, 24.4, 22.9, 22.2, 20.5, 18.3, 16.2, 14.6. Anal. Calcd. for C_23_H_35_NO_2_: C, 77.27; H, 9.87; N, 3.92. Found: C, 77.38; H, 9.90; N, 3.93.

##### *N*-(4-chlorobenzyl)-2-((1*R*,2*R*,4a*S*,8a*S*)-2-hydroxy-2,5,5,8a-tetramethyldecahydronaphthalen-1-yl)acetamide (**5**)

The product was purified by column chromatography using PE/EtOAc (2/1) as eluent to give the compound as a white solid, mp 154–155 °C (G). Yield 70%. IR ν (cm^−1^): 3279, 2924, 1642, 1492, 1387, 1091, 1015, 938, 800. ^1^H NMR (300 MHz, CDCl_3_) δ: 7.25-7.23(m, 2H, Ar), 7.15-7.13 (m, 2H, Ar), 4.35-4.21 (m, 2H, CH_2_NH), 3.69 (brs, 1H, OH), 2.42 (dd, *J*_1_ = 15.4 Hz, *J*_2_ = 4.5 Hz, 1H, CH_2_CO), 2.16 (dd, *J*_1_ = 15.4 Hz, *J*_2_ = 4.6 Hz, 1H, CH_2_CO), 1.90-1.85 (m, 1H, CH), 1.71-1.20 (m, 10H), 1.07 (s, 3H, CH_3_), 0.94-0.90 (m, 1H, CH), 0.86 (s, 3H, CH_3_), 0.77 (s, 3H, CH_3_), 0.74 (s, 3H, CH_3_). ^13^C NMR (75 MHz, CDCl_3_) δ: 175.8, 137.1, 132.9, 128.8 (×2), 128.6 (×2), 72.9, 57.9, 55.9, 44.1, 42.8, 41.7, 39.2, 38.7, 33.3, 33.2, 32.5, 23.7, 21.4, 20.4, 18.3, 15.4. Anal. Calcd. for C_23_H_34_ClNO_2_: C, 70.48; H, 8.74; N, 3.57. Found: C, 70.22; H, 8.77; N, 3.56.

##### *N*-(3,4-dichlorobenzyl)-2-((1*R*,2*R*,4a*S*,8a*S*)-2-hydroxy-2,5,5,8a-tetramethyldecahydronaphthalen-1-yl)acetamide (**6**)

The product was purified by column chromatography using PE/EtOAc (1/1.5) as eluent to give the compound as a white solid, mp 149-150 °C (G). Yield 83%. IR ν (cm^−1^): 3298, 2926, 1642, 1548, 1470, 1388, 1082, 1032, 754. ^1^H NMR (400 MHz, DMSO) δ (ppm): 8.23 (t, 1H, *J* = 6.1 Hz, NH), 7.51 (d, *J* = 8.3 Hz, 1H, Ar), 7.44 (d, *J* = 1.8 Hz, 1H, Ar), 7.20 (dd, *J*_1_ = 8.3 Hz, *J*_2_ = 1.8 Hz, 1H, Ar), 4.26 (dd, *J*_1_ = 15.5 Hz, *J*_2_ = 6.2 Hz, 1H, CH_2_NH), 4.15 (dd, *J*_1_ = 15.5 Hz, *J*_2_ = 5.8 Hz, 1H, CH_2_NH), 2.34 (dd, *J*_1_ = 15.4 Hz, *J*_2_ = 2.8 Hz, 1H, CH_2_CO), 2.05 (dd, *J*_1_ = 15.4 Hz, *J*_2_ = 7.1 Hz, 1H, CH_2_CO), 1.76-1.66 (m, 2H), 1.54-1.02 (mm, 8H), 0.93 (s, 3H, COH(CH_3_)), 0.87-0.82 (m, 2H), 0.80 (s, 3H, CH_3_), 0.72 (s, 6H, CH_3_). ^13^C NMR (100 MHz, DMSO) δ (ppm): 174.6 (C=O), 141.7 (CqAr), 131.3 (CqAr), 130.8 (CHAr), 129.6 (CHAr + CqAr), 128.1 (CHAr), 71.6 (Cq-OH(CH_3_)), 56.8 (CH), 56.0 (CH), 44.2, 42.0, 41.6, 39.2, 38.7, 33.7, 33.3, 31.6, 24.6, 21.8, 20.5, 18.3, 15.5. Anal. Calcd. for C_23_H_32_Cl_2_NO_2_: C, 64.78; H, 7.80; N, 3.28. Found: C, 65.02; H, 7.77; N, 3.29.

##### *N*-(4-fluorobenzyl)-2-((1*R*,2*R*,4a*S*,8a*S*)-2-hydroxy-2,5,5,8a-tetramethyldecahydronaphthalen-1-yl)acetamide (**7**)

The product was purified by column chromatography using PE/EtOAc (1.5/1) as eluent to give the compound as a white solid, mp 135-136 °C (G). Yield 97%. IR ν (cm^−1^): 3675, 2987, 2907, 1510, 1214, 1057, 742, 666. ^1^H NMR (400 MHz, CDCl_3_) δ (ppm): 7.22-7.19 (m, 2H, Ar), 6.97 (t, *J* = 8.6 Hz, 2H, Ar), 6.38 (brs, 1H, NH), 4.37 (dd, *J*_1_ = 15.0 Hz, *J*_2_ = 6.0 Hz, 1H, CH_2_-NH), 4.31 (dd, *J*_1_ = 15.0 Hz, *J*_2_ = 5.9 Hz, 1H, CH_2_-NH), 2.46 (brs, 1H, OH), 2.39 (dd, *J*_1_ = 15.4 Hz, *J*_2_ = 5.2 Hz, 1H, CH_2_CO), 2.14 (dd, *J*_1_ = 15.4 Hz, *J*_2_ = 4.1 Hz, 1H, CH_2_CO), 1.90 (dt, *J*_1_ = 12.5 Hz, *J*_2_ = 3.0 Hz, 1H, -CH_2_-COH(CH_3_)), 1.76 (t, *J* = 4.6 Hz, 1H, -CH-COH(CH_3_)), 1.68-1.52 (m, 2H), 1.50-1.32 (m, 4H), 1.29-1.12 (m, 2H), 1.10 (s, 3H, COH(CH_3_)), 0.97-0.88 (m, 2H), 0.85 (s, 3H, CH_3_), 0.76 (s, 3H, CH_3_), 0.75 (s, 3H, CH_3_). ^13^C NMR (100 MHz, CDCl_3_) δ (ppm): 175.2 (C=O), 162.1 (d, *J* = 267 Hz, Cq-F), 134.3 (CqAr), 129.4 (×2, *J* = 7.9 Hz, CHAr), 115.5 (×2, *J* = 21.3 Hz, CHAr), 73.2 (Cq-OH(CH_3_)), 57.9 (CH), 56.0 (CH), 44.3, 43.0, 41.8, 39.4, 38.8 (Cq-(CH_3_)_2_), 33.3, 32.6, 29.7, 23.8, 21.4, 20.5, 18.4, 15.5. Anal. Calcd. for C_23_H_24_FNO_2_: C, 73.56; H, 9.13; N, 3.73. Found: C, 73.76; H, 9.09; N, 3.72.

##### 2-((1*R*,2*R*,4a*S*,8a*S*)-2-hydroxy-2,5,5,8a-tetramethyldecahydronaphthalen-1-yl)-*N*-(4-methoxybenzyl)acetamide (**8**)

The product was purified by column chromatography using PE/EtOAc (1/1) as eluent to give the compound as a yellow oil. Yield 78%. IR ν (cm^−1^): 3675, 3289, 2920, 1512, 1214, 1038, 748, 666. ^1^H NMR (400 MHz, CDCl_3_) δ (ppm): 7.17 (d, 2H, *J* = 8.4 Hz, Ar), 6.83 (d, 2H, *J* = 8.6 Hz, Ar), 6.23 (brs, 1H, NH), 4.34 (dd, *J*_1_ = 14.8 Hz, *J*_2_ = 5.8 Hz, 1H, *CH_2_*NH), 4.29 (dd, *J*_1_ = 14.8 Hz, *J*_2_ = 5.6 Hz, 1H, *CH_2_*NH), 3.77 (s, 3H, OCH_3_), 2.47 (brs, 1H, OH), 2.39 (dd, *J*_1_ = 15.4 Hz, *J*_2_ = 5.2 Hz, 1H, *CH_2_*CO), 2.12 (dd, *J*_1_ = 15.4 Hz, *J*_2_ = 4.0 Hz, 1H, *CH_2_*CO), 1.90 (dt, *J*_1_ = 9.6 Hz, *J*_2_ = 2.9 Hz, 1H, -*CH_2_*-COH(CH_3_)), 1.78 (t, *J* = 4.5 Hz, 1H, -*CH*-COH(CH_3_)), 1.68-1.45 (mm, 4H), 1.42-1.12 (mm, 4H), 1.10 (s, 3H, COH(*CH_3_*)), 0.97-0.93 (m, 2H), 0.85 (s, 3H, CH_3_), 0.76 (s, 3H, CH_3_), 0.75 (s, 3H, CH_3_). ^13^C NMR (100 MHz, CDCl_3_) δ (ppm): 175.1 (C=O), 159.0 (*CqAr*), 130.5 (*CqAr*), 129.1 (×2, *CHAr*), 114.1 (×2, *CHAr*), 73.1 (*Cq*-OH(CH_3_)), 57.8 (CH), 56.0 (CH), 55.3 (OCH_3_), 44.3, 43.3, 41.8, 39.4, 38.8 (*Cq*-(CH_3_)_2_), 33.3, 33.2, 32.5, 23.7, 21.4, 20.5, 18.4, 15.5. Anal. Calcd. for C_24_H_37_NO_3_: C, 74.38; H, 9.62; N, 3.61. Found: C, 74.42; H, 9.66; N, 3.62.

##### 2-((1*R*,2*R*,4a*S*,8a*S*)-2-hydroxy-2,5,5,8a-tetramethyldecahydronaphthalen-1-yl)-*N*-(3 methoxybenzyl)acetamide (**9**)

The product was purified by column chromatography using PE/EtOAc (1/1) as eluent to give the compound as a yellow oil. Yield 85%. IR ν (cm^−1^): 3291, 2945, 1642, 1264, 1214, 1051, 746, 666. ^1^H NMR (300 MHz, CDCl_3_) δ (ppm): 7.15-7.07 (m, 1H, Ar), 6.74-6.68 (m, 3H, Ar), 4.30-4.17 (m, 2H, *CH_2_*NH), 3.68 (s, 3H, CH_3_O), 2.36 (dd, *J*_1_ = 15.4 Hz, *J*_2_ = 4.4 Hz, 1H, CH_2_CO), 2.09 (dd, *J*_1_ = 15.3 Hz, *J*_2_ = 4.7 Hz, 1H, CH_2_CO), 1.95-1.79 (m, 1H, CH), 1.67 (t, *J* = 4.5 Hz, 1H), 1.57-1.03 (m, 9H, CH_2_), 1.00 (s, 3H, CH_3_), 0.87-0.81 (m, 1H, CH), 0.78 (s, 3H, CH_3_), 0.69 (s, 6H, CH_3_). ^13^C NMR (75 MHz, CDCl_3_) δ (ppm): 178.9, 159.6, 140.1, 129.5, 119.7, 112.9, 112.7, 72.9, 57.8, 55.9, 44.1, 43.4, 41.7, 39.2, 38.7, 33.3, 33.2 (×2), 32.4, 23.6, 21.4, 20.4, 18.3, 15.3. Anal. Calcd. for C_24_H_37_NO_3_: C, 74.38; H, 9.62; N, 3.61. Found: C, 74.48; H, 9.65; N, 3.60.

##### 2-((1*R*,2*R*,4a*S*,8a*S*)-2-hydroxy-2,5,5,8a-tetramethyldecahydronaphthalen-1-yl)-*N*-phenethylacetamide (**10**)

The product was purified by column chromatography using PE/EtOAc (1/1) as eluent to give the compound as a light yellow oil. Yield 95%. ^1^H NMR (300 MHz, CDCl_3_) δ (ppm): 7.29-7.19 (m, 2H, Ar), 7.18-7.10 (m, 2H, Ar), 6.60-6.51 (m, 1H, Ar), 3.54 (s, 1H, OH), 3.48-3.32 (m, 2H, *CH_2_*NH), 2.74 (t, *J* = 6.6 Hz, 2H, CH_2_Ar), 2.02 (dd, *J*_1_ = 14.5 Hz, *J*_2_ = 3.4 Hz, 1H, CH_2_CO), 1.85 (dd, *J*_1_ = 12.3 Hz, *J*_2_ = 5.0 Hz, 1H, CH_2_CO), 1.91-1.80 (m, 1H), 1.65-1.10 (m, 10H), 1.04 (s, 3H, CH_3_), 0.92-0.85 (m, 1H, CH), 0.80 (s, 3H, CH_3_), 0.70 (s, 6H, CH_3_). ^13^C NMR (75 MHz, CDCl_3_) δ (ppm): 175.6, 139.0, 129.8, 129.6, 127.8, 127.5, 125.3, 72.9, 57.2, 55.1, 40.7, 38.7, 35.5, 33.2, 32.6, 24.5, 23.1, 22.2, 20.5, 19.8, 19.1, 18.3, 16.2, 14.6. IR ν (cm^−1^): 3021, 2930, 1655, 1214, 748, 666. Anal. Calcd. for C_24_H_37_NO_2_: C, 77.58; H, 10.04; N, 3.77. Found: C, 77.60; H, 10.07; N, 3.77.

##### *N*-(4-chlorophenethyl)-2-((1*R*,2*R*,4a*S*,8a*S*)-2-hydroxy-2,5,5,8a-tetramethyldecahydronaphthalen-1-yl)acetamide (**11**)

The product was purified by column chromatography using PE/EtOAc (1/1) as eluent to give the compound as a white solid, mp 168 °C (G). Yield 75%. IR ν (cm^−1^): 3298, 2977, 2914, 1634, 1214, 1056, 749, 666. ^1^H NMR (400 MHz, DMSO) δ (ppm): 7.68 (m, 1H, NH), 7.31-7.28 (m, 2H, Ar), 7.22-7.19 (m, 2H, Ar), 4.23 (s, 1H, OH), 3.27-3.20 (m, 2H, *CH_2_*NH), 2.69-2.64 (m, 2H, *CH_2_*-Ar), 2.21 (d, *J* = 15.4 Hz, 1H, *CH_2_*CO), 1.95 (dd, *J*_1_ = 15.3 Hz, *J*_2_ = 6.1 Hz, 1H, *CH_2_*CO), 1.71-1.60 (m, 4H), 1.53-1.40 (m, 2H), 1.37-1.12 (m, 4H), 1.08-1.01 (m, 2H), 0.93 (s, 3H, COH(*CH_3_*)), 0.82 (s, 3H, CH_3_), 0.73 (s, 3H, CH_3_), 0.69 (s, 3H, CH_3_). ^13^C NMR (100 MHz, DMSO) δ (ppm): 174.4 (C=O), 139.1 (*CqAr*), 131.0 (×2, *CHAr* + *CqAr*), 128.6 (×2, *CHAr*), 71.6 (*Cq*-OH(CH_3_)), 56.8 (CH), 56.0 (CH), 44.2, 42.0, 39.4 (under DMSO), 38.9, 38.7 (*Cq*-(CH_3_)_2_), 34.7, 33.8, 33.3, 31.8, 24.6, 21.8, 20.5, 18.4, 15.5. Anal. Calcd. for C_24_H_36_ClNO_2_: C, 71.00; H, 8.94; N, 3.45. Found: C, 71.12; H, 8.97; N, 3.46.

##### *N*-(4-fluorophenethyl)-2-((1*R*,2*R*,4a*S*,8a*S*)-2-hydroxy-2,5,5,8a-tetramethyldecahydronaphthalen-1-yl)acetamide (**12**)

The product was purified by column chromatography using PE/EtOAc (1/1) as eluent to give the compound as a white solid, mp 114–115 °C (G). Yield 83%. IR ν (cm^−1^): 3298, 2970, 2933, 1642, 1509, 1215, 1057, 748, 666. ^1^H NMR (400 MHz, CDCl_3_) δ (ppm): 7.15-7.12 (m, 2H, Ar), 6.96 (t, 2H, *J* = 8.2 Hz, Ar), 6.03 (brs, 1H, NH), 3.52-3.37 (m, 2H, *CH_2_*NH), 2.76 (t, *J* = 6.1 Hz, 2H, *CH_2_*-Ar), 2.47 (brs, 1H, OH), 2.29 (dd, *J*_1_ = 15.3 Hz, *J*_2_ = 5.2 Hz, 1H, *CH_2_*CO), 2.04 (dd, *J*_1_ = 15.3 Hz, *J*_2_ = 3.9 Hz, 1H, *CH_2_*CO), 1.89 (dt, *J*_1_ = 12.5 Hz, *J*_2_ = 3.1 Hz, 1H, -*CH_2_*-COH(CH_3_)), 1.68-1.62 (m, 1H), 1.56-1.51 (m, 2H), 1.42-1.32 (m, 4H), 1.27-1.14 (m, 2H), 1.09 (s, 3H, COH(*CH_3_*)), 0.93-0.90 (m, 2H), 0.85 (s, 3H, CH_3_), 0.75 (s, 3H, CH_3_), 0.73 (s, 3H, CH_3_). ^13^C NMR (100 MHz, CDCl_3_) δ (ppm): 175.4 (C = O), 161.6 (d, *J* = 244 ppm, *Cq*-F), 134.7 (*CqAr*), 130.3 (×2, *J* = 7.5 Hz, *CHAr*), 115.4 (×2, *J* = 21.2 Hz, *CHAr*), 73.1 (*Cq*-OH(CH_3_)), 57.9 (CH), 56.0 (CH), 44.3, 41.8, 40.7, 39.3, 38.7 (*Cq*-(CH_3_)_2_), 34.8, 33.3, 33.2, 32.6, 23.8, 21.4, 20.5, 18.3, 15.4. Anal. Calcd. for C_24_H_36_FNO_2_: C, 74.00; H, 9.32; N, 3.60. Found: C, 73.88; H, 9.36; N, 3.59.

##### *N*-(3-fluorophenethyl)-2-((1*R*,2*R*,4a*S*,8a*S*)-2-hydroxy-2,5,5,8a-tetramethyldecahydronaphthalen-1-yl)acetamide (**13**)

The product was purified by column chromatography using PE/EtOAc (1/1) as eluent to give the compound as an amorphous solid. Yield 72%. IR ν (cm^−1^): 3685, 3310, 2977, 1642, 1215, 1057, 747, 666. ^1^H NMR (300 MHz, CDCl_3_) δ (ppm): 7.27-7.18 (m, 1H, Ar), 7.00-6.84 (m, 2H, Ar), 6.66 (t, *J* = 5.4 Hz, 1H, Ar), 3.53-3.32 (m, 2H, *CH_2_*NH), 2.77 (t, *J* = 6.9 Hz, 2H, CH_2_), 2.34 (dd, *J*_1_ = 15.3 Hz, *J*_2_ = 4.6 Hz, 1H, CH_2_), 2.06 (dd, *J*_1_ = 15.3 Hz, *J*_2_ = 5.3 Hz, 1H, CH_2_), 1.91-1.86 (m, 1H, CH), 1.65-1.10 (m, 10H), 1.07 (s, 3H, CH_3_), 0.95-0.87 (m, 1H, CH), 0.85 (s, 3H, CH_3_), 0.73 (s, 6H, CH_3_). ^13^C NMR (75 MHz, CDCl_3_) δ (ppm): 175.8, 164.5, 161.2, 141.6, 128.9, 125.6, 123.5, 114.8, 112.3, 72.9, 57.2, 56.6, 55.1, 40.5, 38.7, 35.2, 32.6, 31.7, 23.3, 20.9, 18.2, 16.2, 14.9, 14.5. Anal. Calcd. for C_24_H_36_FNO_2_: C, 74.00; H, 9.32; N, 3.60. Found: C, 73.90; H, 9.34; N, 3.61.

##### *N*-(furan-2-ylmethyl)-2-((1*R*,2*R*,4a*S*,8a*S*)-2-hydroxy-2,5,5,8a-tetramethyldecahydronaphthalen-1-yl)acetamide (**14**)

The product was purified by column chromatography using PE/EtOAc (1/1) as eluent to give the compound as a yellow oil. Yield 95%. IR ν (cm^−1^): 3306, 2926, 1648, 1214, 746, 666. ^1^H NMR (400 MHz, CDCl_3_) δ (ppm): 7.31 (s, 1H, Fur), 6.32 (brs, 1H, NH), 6.28 (t, *J* = 1.3 Hz, 1H, Fur), 6.18 (d, *J* = 2.7 Hz, 1H, Fur), 4.40 (dd, *J*_1_ = 15.5 Hz, *J*_2_ = 5.5 Hz, 1H, *CH_2_*NH), 4.36 (dd, *J*_1_ = 15.5 Hz, *J*_2_ = 5.4 Hz, 1H, *CH_2_*NH), 2.92 (brs, 1H, OH), 2.38 (dd, *J*_1_ = 15.4 Hz, *J*_2_ = 5.1 Hz, 1H, *CH_2_*CO), 2.12 (dd, *J*_1_ = 15.4 Hz, *J*_2_ = 4.2 Hz, 1H, *CH_2_*CO), 1.90 (d, *J* = 12.5 Hz, 1H, -*CH_2_*-COH(CH_3_)), 1.76 (t, *J* = 4.5 Hz, 1H, -*CH*-COH(CH_3_)), 1.67-1.51 (m, 2H), 1.49-1.31 (m, 4H), 1.29-1.19 (m, 2H), 1.10 (s, 3H, COH(*CH_3_*)), 0.98-0.89 (m, 2H), 0.84 (s, 3H, CH_3_), 0.76 (s, 6H, CH_3_). ^13^C NMR (100 MHz, CDCl_3_) δ (ppm): 175.1 (C=O), 151.5 (*CqAr*), 142.1 (*CHFur*), 110.4 (*CHFur*), 107.2 (*CHFur*), 73.2 (*Cq*-OH(CH_3_)), 57.8 (CH), 55.9 (CH), 44.3, 41.8, 39.3, 38.7 (*Cq*-(CH_3_)_2_), 36.8, 33.3, 33.2, 32.5, 23.7, 21.4, 20.5, 18.4, 15.5. Anal. Calcd. for C_21_H_33_NO_3_: C, 72.58; H, 9.57; N, 4.03. Found: C, 72.70; H, 9.61; N, 4.03.

##### *N*-((1,1′-biphenyl)-4-ylmethyl)-2-((1*R*,2*R*,4a*S*,8a*S*)-2-hydroxy-2,5,5,8a-tetramethyldecahydronaphthalen-1-yl)acetamide (**15**)

The product was purified by column chromatography using PE/EtOAc (2/1) as eluent to give compound as an amorphous solid. Yield 40%. IR ν (cm^−1^): 3288, 2924, 1637, 1548, 1386, 1123, 938, 759, 696. ^1^H NMR (300 MHz, CDCl_3_) δ (ppm): 7.58-7.52 (m, 4H, Ar), 7.46-7.41 (m, 2H, Ar), 7.37-7.31 (m, 2H, Ar), 6.60 (t, *J* = 5.2 Hz, 1H, Ar), 4.5-4.36 (m, 2H, *CH_2_*NH), 3.15 (brs, 1H, OH), 2.46 (dd, *J*_1_ = 15.4 Hz, *J*_2_ = 4.9 Hz, 1H, CH_2_CO), 2.17 (dd, *J*_1_ = 15.4 Hz, *J*_2_ = 4.3 Hz, 1H, CH_2_CO), 1.94-1.89 (m, 1H), 1.81 (t, *J* = 4.6 Hz, 1H), 1.73-1.21 (m, 9H, CH_2_), 1.12 (s, 3H, CH_3_), 1.00-0.96 (m, 1H), 0.86 (s, 3H, CH_3_), 0.77 (s, 6H, CH_3_). ^13^C NMR (75 MHz, CDCl_3_) δ (ppm): 175.4, 140.7, 140.2, 137.5, 128.8 (×2), 128.1, 127.3 (×2), 127.0, 73.1, 57.8, 55.9, 44.2, 43.4, 41.7, 39.3, 38.7, 33.3, 33.2, 32.6, 31.6, 23.7, 22.7, 21.4, 20.5, 18.4, 15.5, 14.2. Anal. Calcd. for C_29_H_39_NO_2_: C, 80.33; H, 9.07; N, 3.23. Found: C, 80.55; H, 9.11; N, 3.24.

##### *N*-(3-(1*H*-imidazol-1-yl)propyl)-2-((1*R*,2*R*,4a*S*,8a*S*)-2-hydroxy-2,5,5,8a-tetramethyldecahydronaphthalen-1-yl)acetamide (**16**)

Without any further purification, the product was obtained as a light yellow solid, mp 83-84 °C (G). Yield 93%. IR ν (cm^−1^): 3291, 2926, 1644, 1390, 1214, 1082, 747, 666. ^1^H NMR (400 MHz, CDCl_3_) δ (ppm): 7.47 (s, 1H, imid), 7.00 (s, 1H, imid), 6.90 (s, 1H, imid), 6.79 (brt, *J* = 5.3 Hz, 1H, NH), 3.95 (t, *J* = 6.9 Hz, 2H, *CH_2_*-Nimid), 3.23-3.11 (m, 2H, *CH_2_*-NH), 2.72 (brs, 1H, OH), 2.34 (dd, *J*_1_ = 15.2 Hz, *J*_2_ = 5.3 Hz, 1H, *CH_2_*CO), 2.12 (dd, *J*_1_ = 15.2 Hz, *J*_2_ = 4.0 Hz, 1H, *CH_2_*CO), 1.97-1.88 (m, 3H), 1.69 (t, *J* = 4.6 Hz, 1H, -*CH*-COH(CH_3_)), 1.66-1.32 (mm, 6H), 1.28-1.18 (m, 2H), 1.12 (s, 3H, COH(*CH_3_*)), 0.95-0.89 (m, 2H), 0.84 (s, 3H, CH_3_), 0.75 (s, 3H, CH_3_), 0.74 (s, 3H, CH_3_). ^13^C NMR (100 MHz, CDCl_3_) δ (ppm): 175.9 (C=O), 137.2 (*CHimid*), 129.2 (*CHimid*), 119.0 (*CHimid*), 73.3 (*Cq*-OH(CH_3_)), 58.2 (CH), 56.1 (CH), 44.6, 44.3, 41.8, 39.5, 38.8 (*Cq*-(CH_3_)_2_), 36.6, 33.3, 33.2, 32.7, 31.0, 23.8, 21.4, 20.5, 18.4, 15.4. Anal. Calcd. for C_22_H_37_N_3_O_2_: C, 70.36; H, 9.93; N, 11.19. Found: C, 70.24; H, 9.97; N, 11.24.

#### 4.2.3. General Procedure for the Synthesis of Homodrimanyl Acid Ester (**18**) and (**19**)

(+)-Sclareolide (100 mg, 0.4 mmol, 1.0 eq.) was dissolved in hot (60 °C) MeOH (1 mL), and NaOH (64 mg, 1.6 mmol, 4 eq.) was added under stirring. The resulting mixture was stirred for 2 h at 60 °C and then cooled to rt. Diluted HCl was then added until pH 5–6 and the formed precipitate was filtered under vacuum [25]. For the next esterification reaction, the obtained intermediate was dissolved in anhydrous DMF (2 mL); then, the appropriate benzyl bromide (0.4 mmol, 1.0 eq.) and solid K_2_CO_3_ (0.4 mmol, 1 eq.) were added. The reaction mixture was stirred at rt for 24 h and then quenched by addition of water (5 mL). The inorganic phase was extracted with EtOAc (3 × 10 mL) and the combined organic layers were washed with water (20 mL) and brine (20 mL). The whole organic phase was dried over anhydrous Na_2_SO_4_, filtered, and evaporated to dryness. The crude product was purified by flash chromatography on silica gel using the mixture PE/EtOAc as eluent to give the corresponding homodrimanyl acid ester in good yield.

##### Benzyl 2-((1*R*,2*R*,4a*S*,8a*S*)-2-hydroxy-2,5,5,8a-tetramethyldecahydronaphthalen-1-yl)acetate (**18**)

The product was purified by column chromatography using PE/EtOAc (6/1) as eluent to give the compound as a colorless oil. Yield 55%. IR ν (cm^−1^): 3014, 2939, 1718, 1214, 907, 748, 730, 666. ^1^H NMR (300 MHz, CDCl_3_) δ (ppm): 7.45-7.30 (m, 5H, Ar), 5.20-5.10 (m, 2H, CH_2_O), 4.72 (brs, 1H, OH), 2.60 (dd, *J*_1_ = 15.2 Hz, *J*_2_ = 4.4 Hz, 1H, CH_2_CO), 2.38 (dd, *J*_1_ = 15.4 Hz, *J*_2_ = 4.6 Hz, 1H, CH_2_CO), 1.99 (m, 2H), 1.62-1.08 (m, 8H, CH_2_), 1.18 (s, 3H, CH_3_), 1.09-0.98 (m, 1H, CH), 0.90 (s, 3H, CH_3_), 0.81 (s, 6H, CH_3_). ^13^C NMR (75 MHz, CDCl_3_) δ (ppm): 175.5, 136.0, 129.3, 129.2 (×2), 127.4 (×2), 73.1, 66.5, 57.7, 54.9, 43.1, 41.6, 38.5, 33.2, 32.5, 30.6, 29.7, 23.7, 22.3, 20.6, 18.3, 14.5. Anal. Calcd. for C_23_H_34_O_3_: C, 77.05; H, 9.56. Found: C, 77.16; H, 9.60.

##### 3,4-dichlorobenzyl 2-((1*R*,2*R*,4a*S*,8a*S*)-2-hydroxy-2,5,5,8a-tetramethyldecahydronaphthalen-1-yl)acetate (**19**)

The product was purified by column chromatography using PE/EtOAc (6/1) as eluent to give the compound as a colorless oil. Yield 50%. ^1^H NMR (400 MHz, CDCl_3_) δ (ppm): 7.44 (s, 1H, Ar), 7.40 (d, *J* = 8.2 Hz, 1H, Ar), 7.17 (d, *J* = 8.2 Hz, 1H, Ar), 5.02 (ABq, *J* = 12.8 Hz, 2H, CH_2_O), 2.52 (dd, *J* = 16.2 Hz, *J* = 5.8 Hz, 1H, *CH_2_*CO), 2.32 (dd, *J* = 16.2 Hz, *J* = 5.1 Hz, 1H, *CH_2_*CO), 1.92 (dt, *J* = 12.4 Hz, *J* = 2.9 Hz, 1H, -*CH_2_*-COH(CH_3_)), 1.84 (t, *J* = 5.4 Hz, 1H, -*CH*-COH(CH_3_)), 1.69-1.52 (m, 2H), 1.48-1.23 (mm, 6H), 1.12 (s, 3H, COH(*CH_3_*)), 0.99-0.91 (m, 2H), 0.86 (s, 3H, CH_3_), 0.77 (s, 6H, CH_3_). ^13^C NMR (100 MHz, CDCl_3_) δ (ppm): 177.0 (C=O), 141.1 (*CqAr*), 132.6 (*CqAr*), 131.4 (*CqAr*), 130.5 (*CHAr*), 128.8 (*CHAr*), 126.0 (*CHAr*), 86.4 (*Cq*-OH(CH_3_)), 63.9 (O*CH_2_*-Ar), 59.1 (CH), 56.7 (CH), 42.2, 39.5, 38.7 (*Cq*-(CH_3_)_2_), 33.2 (×2), 28.7, 21.6 (×2), 20.9, 20.6, 18.1, 15.1.

#### 4.2.4. General Procedure for the Synthesis of Homodrimanyl Diol Esters (**22**–**24**)

According to a published procedure with some modifications, homodrimanyl diol 21 (80 mg, 0.31 mmol, 1.0 eq.) was dissolved in anhydrous DCM (2 mL) under an inert atmosphere. The appropriate carboxylic acid (0.34 mmol, 1.1 eq.), 1-etil-3-(3-dimetilaminopropil) carbodiimide (EDCI) (0.37 mmol, 1.2 eq.), and 1-ethyl-3-(3-dimethylaminopropyl)carbodiimide (DMAP) (0.031 mmol, 0.1 eq.) was added under stirring to the solution. The mixture was stirred at rt, checking the reaction by TLC, until the starting material disappeared (48–72 h). The reaction was quenched by the addition of water (5 mL) and the inorganic layer was extracted with DCM (2 × 10 mL). The combined organic layers were washed with water (20 mL) and brine (20 mL), dried over anhydrous Na_2_SO_4_, and filtered. The solvent was removed under reduced pressure, and the resulting residue was purified by flash chromatography on silica gel using the mixture PE/EtOAc as eluent to give final ester in good yield [26].

##### 2-((1*R*,2*R*,4a*S*,8a*S*)-2-Hydroxy-2,5,5,8a-tetramethyldecahydronaphthalen-1-yl)ethyl Benzo[*d*][1,3]dioxole-5-carboxylate (**22**)

The product was purified by column chromatography using PE/EtOAc (4/1) as eluent to give the compound as a white solid, mp 155-156 °C (G). Yield 59%. IR ν (cm^−1^): 3675, 2977, 2901, 1705, 1441, 1258, 1214, 1076, 1041, 750, 666. ^1^H NMR (400 MHz, CDCl_3_) δ (ppm): 7.63 (d, *J* = 8.0 Hz, 1H, Ar), 7.45 (s, 1H, Ar), 6.81 (d, *J* = 8.1 Hz, 1H, Ar), 6.01 (s, 2H, OCH_2_O), 4.36-4.29 (m, 2H, CH_2_-OCO), 1.91-1.81 (m, 2H), 1.77-1.59 (m, 4H), 1.45-1.34 (m, 3H), 1.31-1.10 (mm, 7H), 0.97-0.90 (m, 2H), 0.85 (s, 3H, CH3), 0.79 (s, 3H, CH_3_), 0.78 (s, 3H, CH_3_). ^13^C NMR (100 MHz, CDCl_3_) δ (ppm): 166.1 (C=O), 151.5 (*CqAr*), 147.7 (*CqAr*), 125.3 (*CHAr*), 124.6 (*CqAr*), 109.6 (*CHAr*), 108.0 (*CHAr*), 101.8 (OCH_2_O), 73.7 (*Cq*-OH(CH_3_)), 67.1 (CH_2_-O), 58.1 (CH), 56.1 (CH), 44.5, 41.9, 39.8, 38.8 (*Cq*-(CH_3_)_2_), 33.4, 33.3, 24.7, 24.0, 21.5, 20.5, 18.4, 15.3. Anal. Calcd for C_24_H_34_O_5_: C, 71.61; H, 8.51. Found: C, 71.87; H, 8.55.

##### 2-((1*R*,2*R*,4a*S*,8a*S*)-2-hydroxy-2,5,5,8a-tetramethyldecahydronaphthalen-1-yl)ethyl (*E*)-3-(benzo[*d*][1,3]dioxol-5-yl)acrylate (**23**)

The product was purified by column chromatography using PE/EtOAc (4/1) as eluent to give the compound as a white solid, mp 115–116 °C (G). Yield 65%. IR ν (cm^−1^): 2983, 2901, 1214, 1057, 744, 668. ^1^H NMR (400 MHz, CDCl_3_) δ (ppm): 7.57 (d, *J* = 15.9 Hz, 1H, CH=*CH*-Ar), 7.00 (s, 1H, Ar), 6.98 (d, *J* = 8.0 Hz, 1H, Ar), 6.78 (d, *J* = 8.0 Hz, 1H, Ar), 6.22 (d, *J* = 15.9 Hz, 1H, *CH*=CH-Ar), 5.98 (s, 2H, OCH_2_O), 4.28-4.17 (m, 2H, CH_2_-OCO), 1.88 (dt, *J* = 12.3 Hz, *J* = 2.9 Hz, 1H, -*CH_2_*-COH(CH_3_)), 1.83-1.53 (m, 4H), 1.46-1.34 (m, 4H), 1.31-1.20 (m, 2H), 1.16 (s, 3H, COH(*CH_3_*)), 1.14-1.09 (m, 2H), 0.95-0.90 (m, 2H), 0.85 (s, 3H, CH_3_), 0.79 (s, 3H, CH_3_), 0.77 (s, 3H, CH_3_). ^13^C NMR (100 MHz, CDCl_3_) δ (ppm): 167.3 (C=O), 149.6 (*CqAr*), 148.4 (*CqAr*), 144.5 (=*CH*-Ar), 128.9 (*CqAr*), 124.4 (*CHAr*), 116.2 (*CH*=CHAr), 108.6 (*CHAr*), 106.6 (*CHAr*), 101.6 (OCH_2_O), 73.6 (*Cq*-OH(CH_3_)), 66.6 (CH_2_-O), 58.1 (CH), 56.1 (CH), 44.4, 41.9, 39.7, 38.8 (*Cq*-(CH_3_)_2_), 33.4, 33.3, 24.7, 24.0, 21.5, 20.5, 18.4, 15.3. Anal. Calcd for C_26_H_36_O_5_: C, 72.87; H, 8.47. Found: C, 73.01; H, 8.49.

##### 2-((1*R*,2*R*,4a*S*,8a*S*)-2-hydroxy-2,5,5,8a-tetramethyldecahydronaphthalen-1-yl)ethyl 4-(thiophen-2-yl)butanoate (**24**)

The product was purified by column chromatography using PE/EtOAc (3/1) as eluent to give the compound as a colorless oil. Yield 70%. IR ν (cm^−1^): 3675, 2958, 1724, 1390, 1214, 1082, 748, 692, 667. ^1^H NMR (400 MHz, CDCl_3_) δ (ppm): 7.10 (d, *J* = 5.0 Hz, 1H, *thienyl*), 6.90 (t, *J* = 4.2 Hz, 1H, *thienyl*), 6.78 (d, *J* = 2.4 Hz, 1H, *thienyl*), 4.17-4.06 (m, 2H, CH_2_-OCO), 2.86 (t, *J* = 7.5 Hz, 2H, COCH_2_-CH_2_-*CH_2_*), 2.34 (t, *J* = 7.5 Hz, 2H, CO*CH_2_*-CH_2_-CH_2_), 2.03-1.95 (m, 2H, COCH_2_-*CH_2_*-CH_2_), 1.87 (d, *J* = 12.3 Hz, 1H, -*CH_2_*-COH(CH_3_)), 1.77-1.69 (m, 1H), 1.67-1.52 (mm, 6H), 1.43-1.35 (m, 3H), 1.30-1.19 (m, 1H), 1.14 (s, 3H, COH(*CH_3_*)), 1.12-1.07 (m, 1H), 0.92-0.88 (m, 2H), 0.86 (s, 3H, CH_3_), 0.77 (s, 6H, CH_3_). ^13^C NMR (100 MHz, CDCl_3_) δ (ppm): 173.4 (C=O), 144.1 (*Cqthienyl*), 126.8 (*CHthienyl*), 124.5 (*CHthienyl*), 123.2 (*CHthienyl*), 73.6 (*Cq*-OH(CH_3_)), 66.6 (CH_2_-O), 58.0 (CH), 56.1 (CH), 44.4, 41.9, 39.7, 38.8 (*Cq*-(CH_3_)_2_), 33.5, 33.4, 33.3, 29.2, 26.8, 24.5, 24.0, 21.5, 20.5, 18.4, 15.3. Anal. Calcd for C_24_H_38_O_3_S: C, 70.89; H, 9.42. Found: C, 70.75; H, 9.44.

##### Synthesis of (1*R*,2*R*,4a*S*,8a*S*)-1-(2-((3-Chlorobenzyl)oxy)ethyl)-2,5,5,8a-tetramethyldecahydronaphthalen-2-ol (homodrimanyl diol ether) (**25**)

Homodrimanyl diol **20** (90 mg, 0.35 mmol, 1.0 eq.) was dissolved in anhydrous THF (10 mL) under argon atmosphere and NaH, previously purified, (67.0 mg, 0.4 mmol, 1.2eq) was added. The reaction mixture was refluxed for 30 min and then, after cooling at rt, 3-chlorobenzyl bromide (67.6 mg, 0.42 mmol, 1.2 eq.) was added. The mixture was still heated to reflux for 48 h, cooled to rt and quenched with water and saturated NH_4_Cl solution (pH 7). Afterward, the aqueous layer was extracted with EtOAc (3 × 15 mL) and the combined organic phases were washed with water (30 mL) and brine (30 mL), dried over anhydrous Na_2_SO_4_, filtered, and evaporated to dryness to obtain the final homodrimanyl diol ether. Column chromatography with PE/EtOAc (8/1) as eluent gave the pure compound as a light yellow oil. Yield 27%. IR ν (cm^−1^): 2926, 1214, 1077, 750, 667. ^1^H NMR (300 MHz, CDCl_3_) δ (ppm): 7.39-7.18 (m, 4H), 4.49 (s, 2H, CH2-Ar), 3.68-3.57 (m, 1H), 3.41-3.30 (m, 1H), 3.19 (brs, 1H, OH), 1.96-1.85 (m, 1H), 1.83-1.70 (m, 1H), 1.69-1.48 (m, 3H), 1.44-1.17 (m, 8H), 1.14 (s, 3H, CH_3_), 0.96-0.90 (m, 1H), 0.88 (s, 3H, CH_3_), 0.78 (s, 6H, CH3). ^13^C NMR (75 MHz, CDCl_3_) δ (ppm): 139.9, 129.8, 127.8, 127.8, 126.5, 125.7, 72.5, 72.3, 72.2, 59.0, 56.0, 43.9, 41.8, 39.5, 38.9, 33.4, 33.2, 25.2, 24.3, 21.5, 20.4, 18.4, 15.3. Anal. Calcd for C_23_H_35_ClO_2_: C, 72.89; H, 8.31. Found: C, 72.56; H, 8.34.

### 4.3. TRPV1 and TRPV4 Channel Assays

Compound effects on intracellular Ca^2+^ concentration ([Ca^2+^]_i_) were determined using the selective intracellular fluorescent probe for Ca^2+^ Fluo-4 and assays were performed as described [27]. Briefly, human embryonic kidney (HEK-293) cells, stably transfected with recombinant rat TRPV4 or human TRPV1 (selected by Geneticin 600 μg/mL) or not transfected, were cultured in EMEM (Eagle’s Minimum Essential Medium) +2 mM Glutamine +1% Non-Essential Amino Acids +10% FBS (fetal bovine serum) and maintained at 37 °C with 5% CO_2_. On the day of the experiment the cells were loaded in the dark at rt for 1 h with Fluo-4 AM (4 μM in DMSO containing 0.02% Pluronic F-127). After that the cells were rinsed and resuspended in Tyrode’s solution (145 mM NaCl, 2.5 mM KCl, 1.5 mM CaCl_2_, 1.2 mM MgCl_2_, 10 mM D-glucose, and 10 mM HEPES (4-(2-hydroxyethyl)-1-piperazine ethane sulfonic acid), pH 7.4) then transferred to a quartz cuvette of a spectrofluorimeter (Perkin-Elmer LS50B Beaconsfield UK; λEX = 488 nm, λEM = 516 nm) under continuous stirring. Cell fluorescence before and after the addition of various concentrations of test compounds was measured normalizing the effects against the response to ionomycin (4 μM). The values of the effect on [Ca^2+^]_i_ in not-transfected HEK-293 cells are used as a baseline and subtracted from the values obtained from transfected cells. The potency of the compounds (EC_50_ values) is determined as the concentration required to produce half-maximal increases in [Ca^2+^]_i_. Antagonist behavior is evaluated against the GSK1016790A agonist of TRPV4 (10 nM) [28] and analyzed by adding the compounds directly in the quartz cuvette 5 min before stimulation of cells with the agonist. IC_50_ is expressed as the concentration exerting a half-maximal inhibition of agonist effect, taking as 100% the effect on [Ca^2+^]_i_ exerted by GSK1016790A (10 nM) alone. Similarly, for TRPV1 using agonist capsaicin 0.1 μM. Dose-response curve fitting (sigmoidal dose-response variable slope) and parameter estimation were performed with Graph-Pad Prism8^®^ (GraphPad Software Inc., San Diego, CA, USA). All determinations were performed at least in triplicate.

### 4.4. MTT Assay

HeLa and A549 cells were seeded at 2 × 10^3^ cells/cm^2^ density in 24-well plastic plates. One day after plating, compounds **6** and **18** were added to the culture medium for 24 h. Cell viability was evaluated with the 3-(4,5-dimethylthiazol-2-yl)-2,5-diphenyltetrazolium bromide (MTT; 0.5 mg/mL; Sigma-Aldrich, Milan, Italy) reduction assay, and formazan salt formation upon MTT reduction by the mitochondria of living cells was detected spectrophotometrically at 595 nm according to published procedures [29].

## 5. Conclusions

In this study, 22 novel trans-decalin-related homodrimane molecules were designed and synthesized, inspired by the natural diversity of labdane diterpenes, bioactive compounds largely occurring in terrestrial and marine sources. The validity of this idea was attested by the identification of a new class of TRPV4 antagonists, active at low micromolar concentration, and endowed with selectivity over TRPV1. The most interesting compounds belong to the homodrimanyl acid amide series, in particular, benzyl and phenylethyl amides. From the SAR study, several critical determinants for activity emerged: (1) the length of the spacer connecting the homodrimane skeleton to the aromatic moiety; (2) the nature of the functional group inside the spacer and, in particular, the presence and position of the carbonyl functionality; and (3) the size and electronic properties of substituents decorating the phenyl ring. The optimal spacer length ranged from four to five atoms, containing a carbonyl group. Thus, this feature represented a quite stringent structural requirement. The presence of an aromatic core is mandatory for activity since both natural (+)-sclareolide and compounds **20** and **21** were inactive. These results are consistent with the observed antagonism of onydecalin A, also featuring an aromatic-substituted *trans*-decalin scaffold and a carbonyl linker. Some of the newly synthesized derivatives exhibited a 9-fold potency increase on onydecalin A activity, an effect probably ascribable to the optimization of the spacer connecting the homodrimane skeleton to the aromatic moiety. In particular, compound **6**, the 3,4-dichlorobenzyl homodrimanyl acid amide, and compound **18**, the benzyl homodrimanyl acid ester, behaved as potent and selective TRPV4 antagonists. The biological results presented herein confirm that the bicyclic *trans*-decalin nucleus is a valuable scaffold for the design of new TRPV4 modulators and prompts us to further investigate this field with the aim to obtain new molecules potentially useful for the treatment of pain and pulmonary oedema linked to COVID-19 syndrome, and to search for other scaffolds in the available and continuously increasing libraries of natural labdane derivatives and related compounds.

## Figures and Tables

**Table 1:**
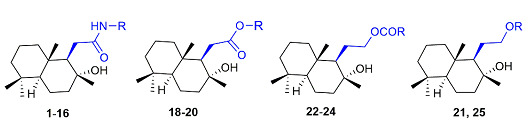
Results of TRPV4 assay of compounds **1**–**16**, **18**–**20**, **22**–**25**. ^a^

Cpd.	R	Efficacy ^b^ %	PotencyEC50 (µM)	IC_50_ (µM) ^c^ inh. TRPV4	Cpd.	R	Efficacy ^b^ %	Potency EC50 (µM)	IC_50_ (µM)^c^ inh. TRPV4
**1**	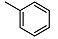	<10	NA ^d^	>100	**14**	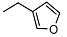	<10	NA	53.5 ± 1.8
**2**	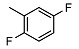	<10	NA	>100	**15**	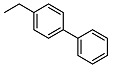	<10	NA	>100
**3**	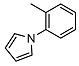	14.6 ± 1.5	1.1 ± 1.0	6.0 ± 0.1	**16**	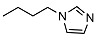	<10	NA	>100
**4**	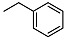	<10	NA	32.0 ± 0.8	**18**	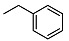	<10	NA	5.41 ± 0.07
**5**	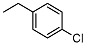	<10	NA	7.7 ± 0.3	**19**	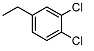	<10	NA	>100
**6**	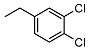	<10	NA	5.3 ± 0.3	**20**	Me	<10	NA	>100
**7**	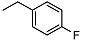	15.8 ± 0.8	13.4 ± 2.6	16.9 ± 0.8	**21**	H	<10	NA	>100
**8**	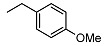	<10	NA	29.7 ± 0.7	**22**	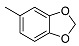	<10	NA	NA
**9**	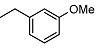	<10	NA	18.1 ± 0.2	**23**	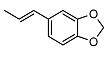	<10	NA	>100
**10**	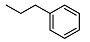	<10	NA	15.6 ± 0.3	**24**	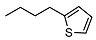	<10	NA	>100
**11**	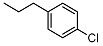	<10	NA	7.0 ± 0.1	**25**	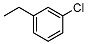	<10	NA	>100
**12**	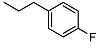	<10	NA	11.4 ± 0.1	**Scd** ^e^		11.3 ± 0.7	> 10	>100
**13**	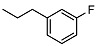	<10	NA	11.9 ± 0.4					

^a^ Data are means ± SEM (standard error of the mean) of at least *n* = 3 determinations. ^b^ As percent of the effect of ionomycin (4 µM). Inh = inhibitory activity. ^c^ Determined against the effect of GSK1016790A (10 nM) after a 5-min pre-incubation with each compound. ^d^ NA = not active, if the efficacy is lower than 10%, the potency is not calculated, ^e^ Scd = (+)-Sclareolide. GSK1016790A efficacy 88.5 ± 1.1 EC_50_ 3.5 ± 0.2 nM.

## Supplementary Materials

The following are available online at https://www.mdpi.com/1660-3397/18/10/519/s1. Procedure for the synthesis and, physical and chemical data of compounds **20** and **21**. Representations of the ^1^H-NMR and ^13^C-NMR spectra of compounds **1**–**16**, **18**–**19**, **22**–**25**. Biological data on TRPV1 assay.
