# Supplementary material for: Design, Synthesis and In Vitro Experimental Validation of Novel TRPV4 Antagonists Inspired by Labdane Diterpenes
Source: Mar Drugs. 2020 Oct 18;18(10):519. doi: 10.3390/md18100519 (PMC7594054; doi:10.3390/md18100519)
Supplement: Supplementary file 1 [file marinedrugs-18-00519-s001.pdf]

marine drugs

Supplementary Material for:

Design, Synthesis and In Vitro Experimental Validation of Novel TRPV4 Antagonists Inspired by Labdane Diterpenes

Sarah Mazzotta [1,2,†], Gabriele Carullo [1,3,†], Aniello Schiano Moriello [4,5], Pietro Amodeo [6], Vincenzo Di Marzo [4,7], Margarita Vega-Holm [2], Rosa Maria Vitale [ψ,*], Francesca Aiello [1,*], Antonella Brizzi [3,‡] and Luciano De Petrocellis [4,‡]

[1] Department of Pharmacy, Health and Nutritional Sciences, DoE 2018–2022, University of Calabria, Edificio Polifunzionale, 87036 Rende (CS), Italy; sarmaz1@alum.us.es (S.M.); gabriele.carullo@unisi.it (G.C.)
[2] Department of Organic and Medicinal Chemistry, Faculty of Pharmacy, University of Seville, Profesor García González 2, 41071 Seville, Spain; mvegaholm@us.es
[3] Department of Biotechnology, Chemistry and Pharmacy, DoE 2018–2022, University of Siena, Via Aldo Moro 2, 53100 Siena, Italy; brizzi3@unisi.it
[4] Endocannabinoid Research Group (ERG), Institute of Biomolecular Chemistry, National Research Council (ICB-CNR), Via Campi Flegrei 34, 80078 Pozzuoli (NA), Italy; aniello.schianomoriello@icb.cnr.it (A.S.M.); vincenzo.dimarzo@criucpq.ulaval.ca (V.D.M.); luciano.depetrocellis@icb.cnr.it (L.D.P.)
[5] Epitech Group SpA, Saccolongo, Padova, Italy
[6] Institute of Biomolecular Chemistry, National Research Council (ICB-CNR), Via Campi Flegrei 34, 80078 Pozzuoli, (NA), Italy; pamodeo@icb.cnr.it
[7] Canada Excellence Research Chair on the Microbiome-Endocannabinoidome Axis in Metabolic Health (CERC-MEND)-Université Laval, Quebec, QC, Canada
[*] Correspondence: rmvitale@icb.cnr.it (R.M.V.); francesca.aiello@unical.it (F.A.)
[†] These authors contributed equally.
[‡] Antonella Brizzi and Luciano De Petrocellis are joint senior authors.

Contents

Table S1. Results of TRPV1 assay of compounds 1-16, 18-20, 22-25……………………….**S3**

Synthesis, yield, melting points of compounds **20** and **21**. …………………………………**S3**

Representations of the ^{1}H-NMR and ^{13}C-NMR spectra of all final compounds. …..……...**S5**

Table S1. Results of TRPV1 assay of compounds **1-16, 18-20, 22-25.**[a]

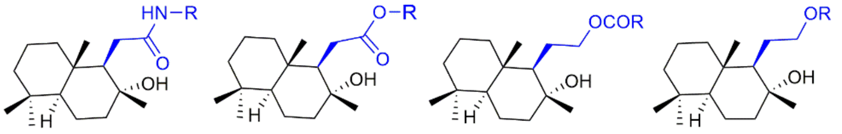

1-16 **18-20** **22-24** **21, 25**

Cpd.	R	Efficacy[b] %	Potency EC_{50} (μM)	IC_{50} (μM)[c] inh TRPV1	Cpd.	R	Efficacy[b] %	Potency EC_{50} (μM)	IC_{50} (μM)[c] inh TRPV1
**1**	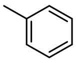	33.6 ± 0.8	>10	> 100	**14**	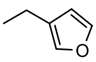	25.2 ± 2.0	> 10	> 100
**2**	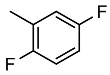	< 10	NA[d]	> 100	**15**	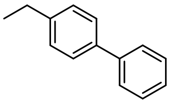	< 10	NA	> 100
**3**	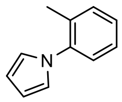	32.0 ± 1.2	4.7 ± 0.4	> 100	**16**	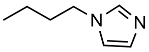	< 10	NA	> 100
**4**	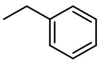	36.8 ± 0.2	> 10	> 10	**18**	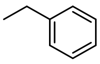	< 10	NA	>10
**5**	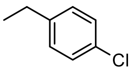	< 10	NA	> 100	**19**	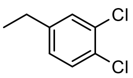	17.5 ± 2.7	>10	> 100
**6**	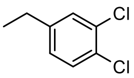	16.9 ± 0.3	> 10	> 100	**20**	Me	< 10	NA	> 100
**7**	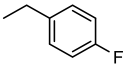	24.5± 0.2	9.9 ± 0.1	>50	**21**	H	< 10	NA	> 100
**8**	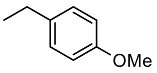	19.7 ± 1.1	> 10	> 100	**22**	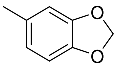	<10	NA	>100
**9**	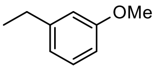	< 10	NA	> 100	**23**	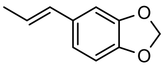	< 10	NA	> 100
**10**	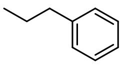	< 10	NA	>10	**24**	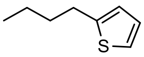	< 10	NA	> 100
**11**	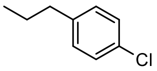	< 10	NA	> 100	**25**	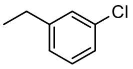	< 10	>10	> 100
**12**	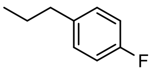	< 10	NA	> 100	**Scd**[e]	-	12.6 ± 0.5	> 10	> 100
**13**	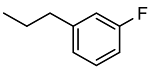	20.8 ± 1.6	> 10	>50					

[a] Data are means ± SEM of at least N = 3 determinations. [b] As percent of the effect of ionomycin (4 μM). Inh = inhibitory activity. [c] Determined against the effect of Capsaicin (100 nM) after a 5 min pre-incubation with each compound. [d]NA = not active, if the efficacy is lower than 10% the potency is not calculated, [e] Scd = (+)-Sclareolide. Capsaicin efficacy 78.6 +/- 0.6 EC50 5.3 +/- 0.4 nM [1]

[1] Del Prete D, Caprioglio D, Appendino G, Minassi A, Schiano-Moriello A, Di Marzo V, De Petrocellis L. Discovery of non-electrophilic capsaicinoid-type TRPA1 ligands. Bioorg Med Chem Lett. 2015 Mar 1;25(5):1009-11. doi: 10.1016/j.bmcl.2015.01.039. Epub 2015 Jan 28. PMID: 25666822.

Synthesis of methyl ester derivative (20).

A well stirred methanolic solution of (+)-sclareolide (100 mg, 3 mL) was heated at 45 ℃ for 72 h. After that, the mixture was evaporated to dryness and the pure compound obtained after flash column chromatography using a gradient of PE/EtOAc. The compound was isolated as a white solid. Mp 72-73 ℃ (G). NMR data are in agreement with those reported. [1] Anal. Calcd. for $C_{17}H_{30}O_3$: C, 72.30; H, 10.71. Found: C, 72.56; H, 10.75.

[1] Carmna, R.M. and Deeth, H.C. Diterpenoids XXVII. The Synthesis of α-Onoceradiene from Abienol. *Aust. J. Chem.* **1971**, *24*, 1099-1102.

Synthesis of (1*R*,2*R*,4a*S*,8a*S*)-1-(2-hydroxyethyl)-2,5,5,8a-tetramethyldecahydronaphthalen-2-ol (homodrimanyl diol) (21). (+)-Sclareolide (300 mg, 1.2 mmol, 1.0 eq.) was dissolved in dry THF (50 mL) under argon and cooled to 0 ℃. Then, $LiAlH_4$ (455.4 mg, 12.0 mmol, 10.0 eq.) was added to the solution. The reaction mixture was stirred at rt for 6 h, then quenched with EtOAc (30 mL) and evaporated to dryness. The residue was dissolved in CH_2Cl_2 (50 mL) and the organic phase washed twice with 1 N HCl (30 mL), with staturated aqueous $NaHCO_3$ (30 mL), and brine (30 mL). The organic phase was finally dried over anhydrous Na_2SO_4, filtered and concentrated under vacuum to give homodrimanyl diol **21** as a white crystalline solid in quantitative yield. Mp 129.5-130.5 °C. ^{1}H-NMR data are in agreement with those reported. [2] Anal. Calcd. for $C_{17}H_{30}O_3$: C, 72.30; H, 10.71. Found: C, 72.56; H, 10.75.

[2] Li, D.; Zhang, S.; Song, Z.; Wang, G.; Li, S. Bioactivity-Guided Mixed Synthesis Accelerate the Serendipity in Lead Optimization: Discovery of Fungicidal Homodrimanyl Amides. *Eur. J. Med. Chem.* **2017**, *136* (31401777), 114–121. https://doi.org/10.1016/j.ejmech.2017.04.073.

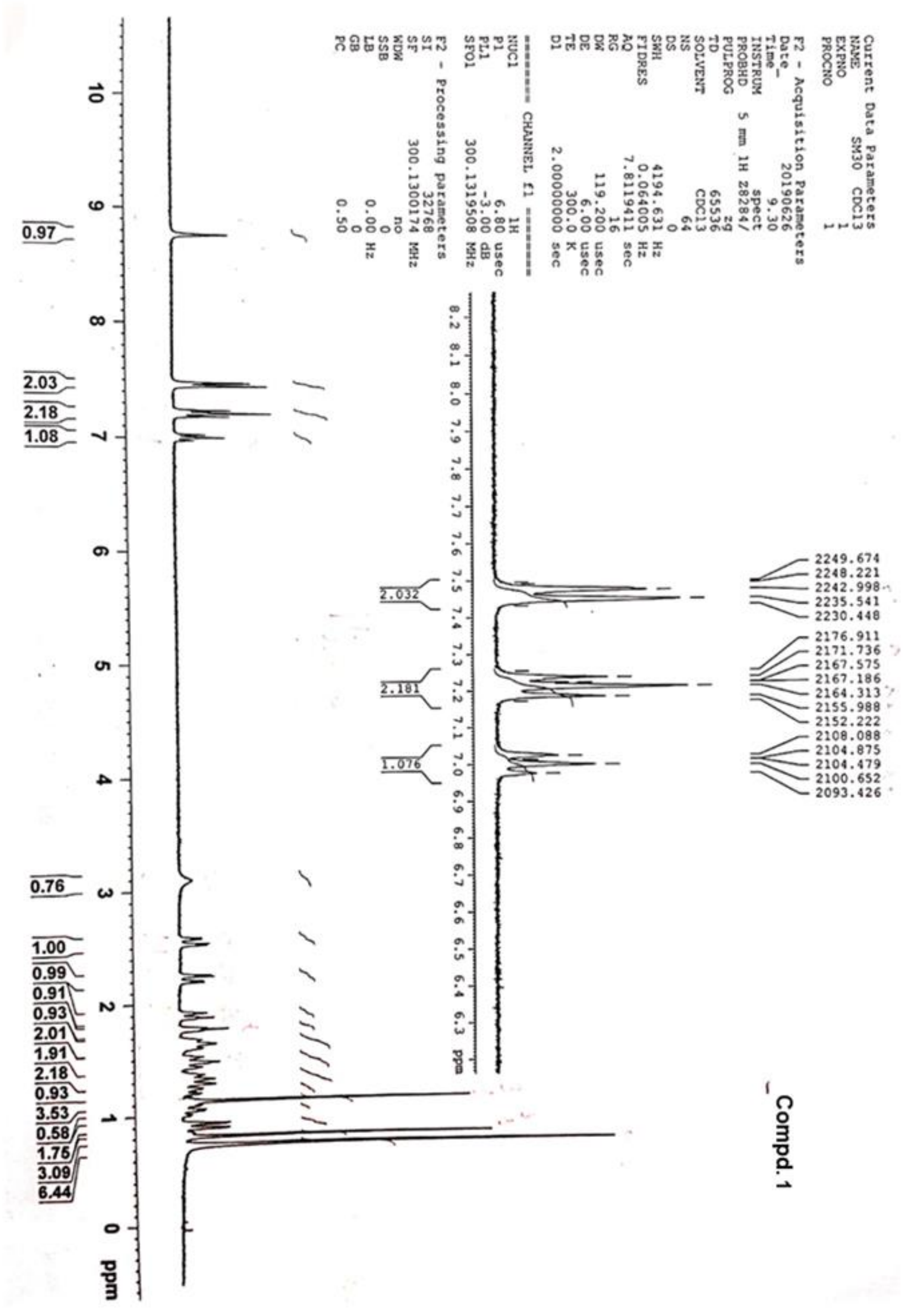
Compd. 1
Current Data Parameters
NAME SM30 CDCl3
EXPNO 1
PROCNO 1
F2 - Acquisition Parameters
Date_ 20190626
Time 9.30
INSTRUM spect
PROBHD 5 mm 1H Z8284/
PULPROG zg
TD 65536
SOLVENT CDCl3
NS 64
DS 0
SWH 4194.631 Hz
FIDRES 0.064005 Hz
AQ 7.8119411 sec
RG 16
DW 119.200 usec
DE 6.00 usec
TE 300.0 K
D1 2.00000000 sec
======== CHANNEL f1 ========
NUC1 1H
P1 6.80 usec
PL1 -3.00 dB
SFO1 300.1319508 MHz
F2 - Processing parameters
SI 32768
SF 300.1300174 MHz
WDW no
SSB 0
LB 0.00 Hz
GB 0
PC 0.50
2249.674
2248.221
2242.998
2235.541
2230.448
2176.911
2171.736
2167.575
2167.186
2164.313
2155.988
2152.222
2108.088
2104.875
2104.479
2100.652
2093.426
2.032
2.181
1.076
0.97
2.03
2.18
1.08
0.76
1.00
0.99
0.91
0.93
2.01
1.91
2.18
0.93
3.53
0.58
1.75
3.09
6.44
ppm

Compd. 1

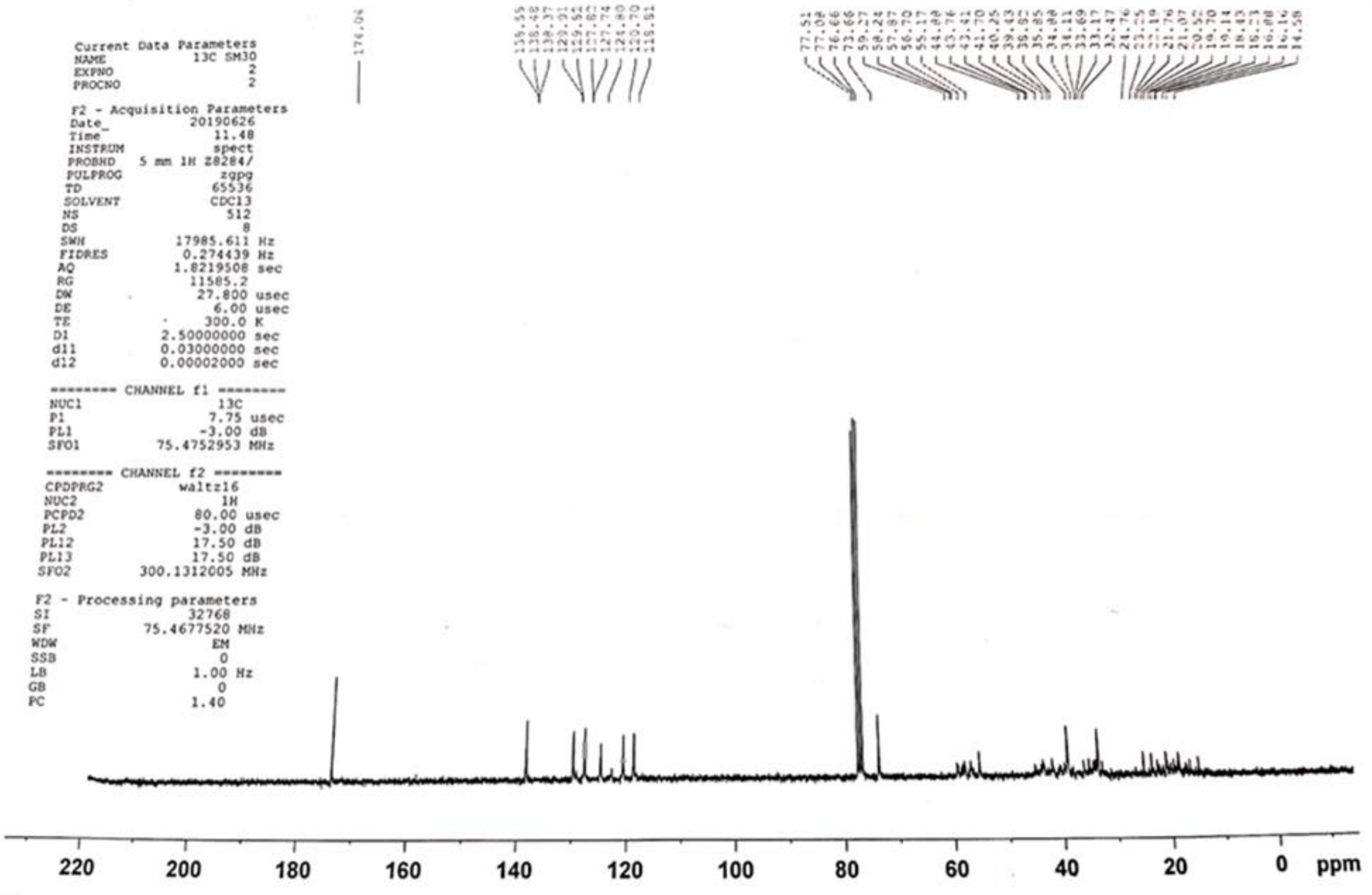
Current Data Parameters
NAME 13C SM30
EXPNO 2
PROCNO 2
F2 - Acquisition Parameters
Date_ 20190626
Time 11.48
INSTRUM spect
PROBHD 5 mm 1H Z8284/
PULPROG zgpg
TD 65536
SOLVENT CDCl3
NS 512
DS 8
SWH 17985.611 Hz
FIDRES 0.274439 Hz
AQ 1.8219508 sec
RG 11585.2
DW 27.800 usec
DE 6.00 usec
TE 300.0 K
D1 2.50000000 sec
d11 0.03000000 sec
d12 0.00002000 sec
======== CHANNEL f1 ========
NUC1 13C
P1 7.75 usec
PL1 -3.00 dB
SFO1 75.4752953 MHz
======== CHANNEL f2 ========
CPDPRG2 waltz16
NUC2 1H
PCPD2 80.00 usec
PL2 -3.00 dB
PL12 17.50 dB
PL13 17.50 dB
SFO2 300.1312005 MHz
F2 - Processing parameters
SI 32768
SF 75.4677520 MHz
WDW EM
SSB 0
LB 1.00 Hz
GB 0
PC 1.40
174.04
138.48
138.37
129.91
127.74
124.80
120.70
118.81
77.51
77.08
76.66
73.66
59.27
58.24
57.87
56.70
55.17
44.88
43.76
43.41
41.70
40.25
39.43
38.82
35.85
34.88
34.11
33.69
33.17
32.47
24.76
23.25
21.76
21.07
19.70
19.14
18.43
16.16
14.58
220
200
180
160
140
120
100
80
60
40
20
0
ppm

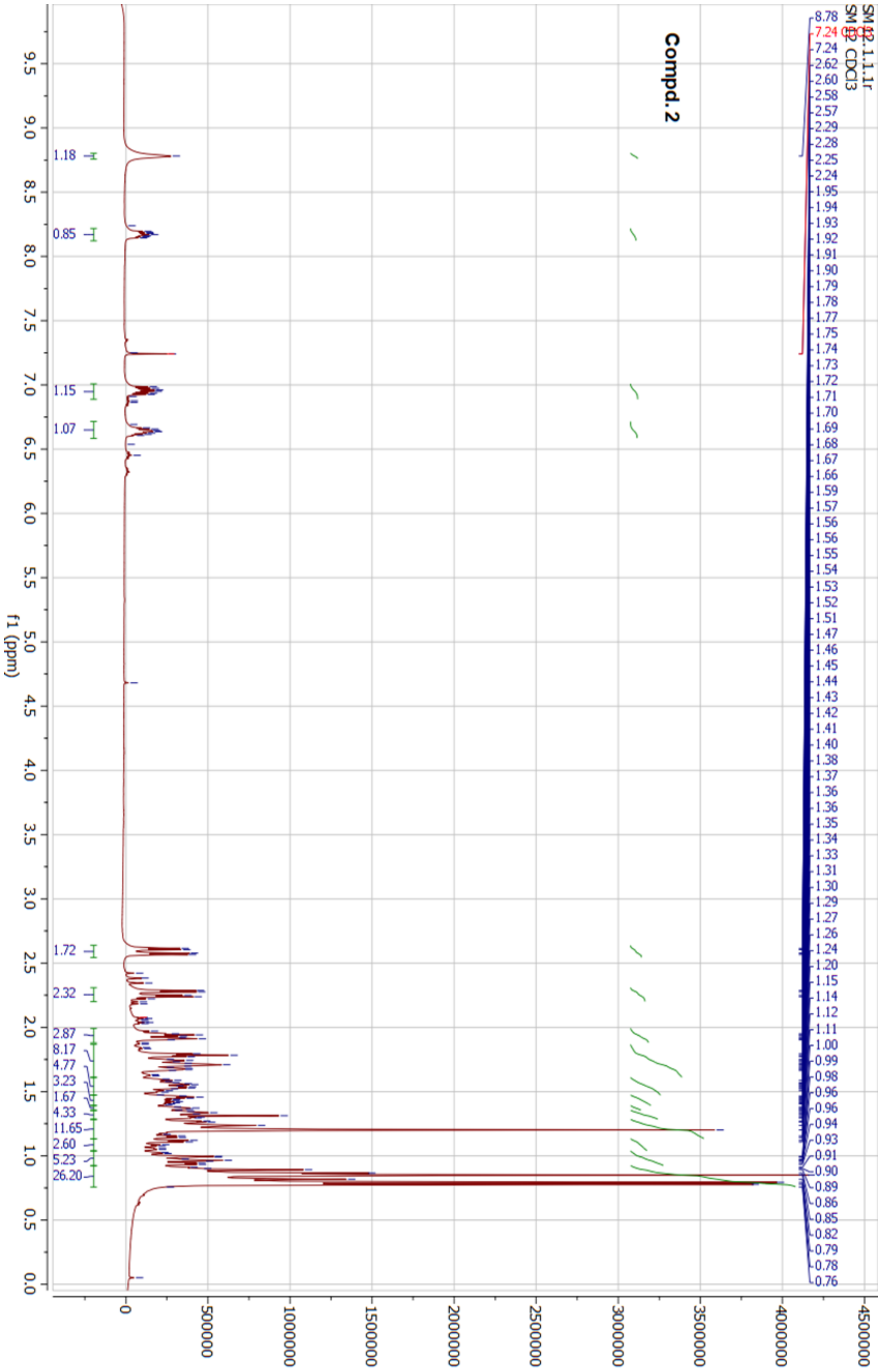
Compd. 2
f1 (ppm)

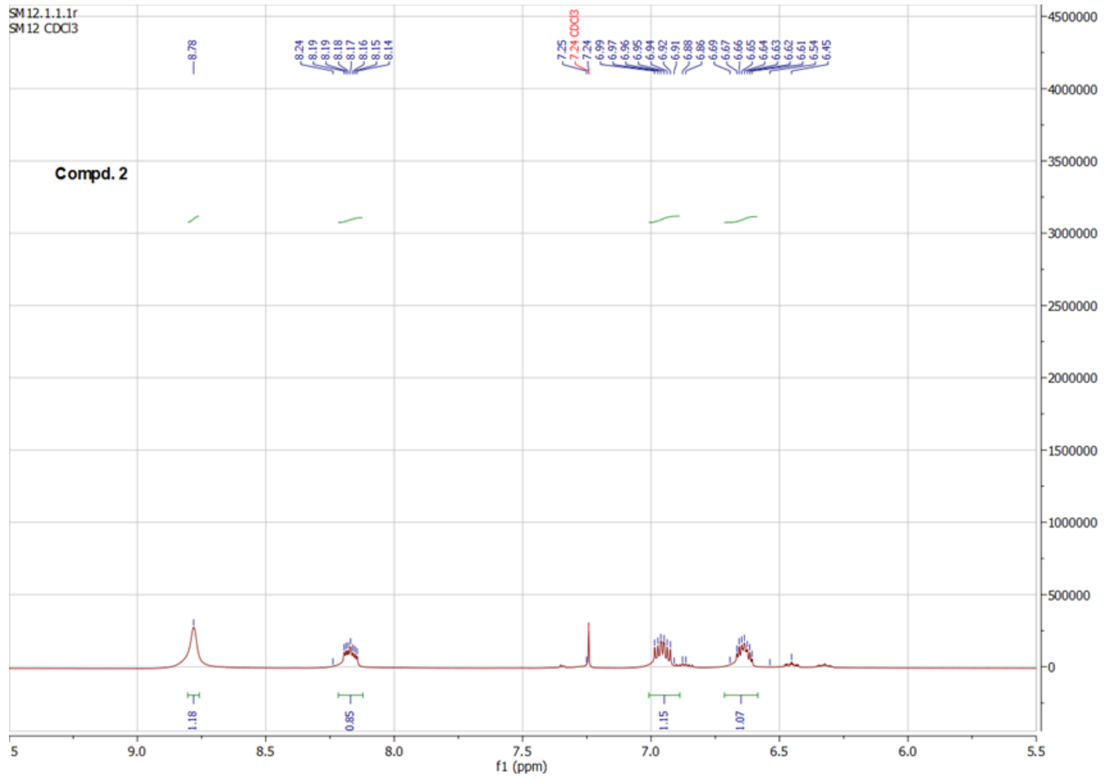
SM12.1.1.1r
SM12 CDCl3
Compd. 2
f1 (ppm)

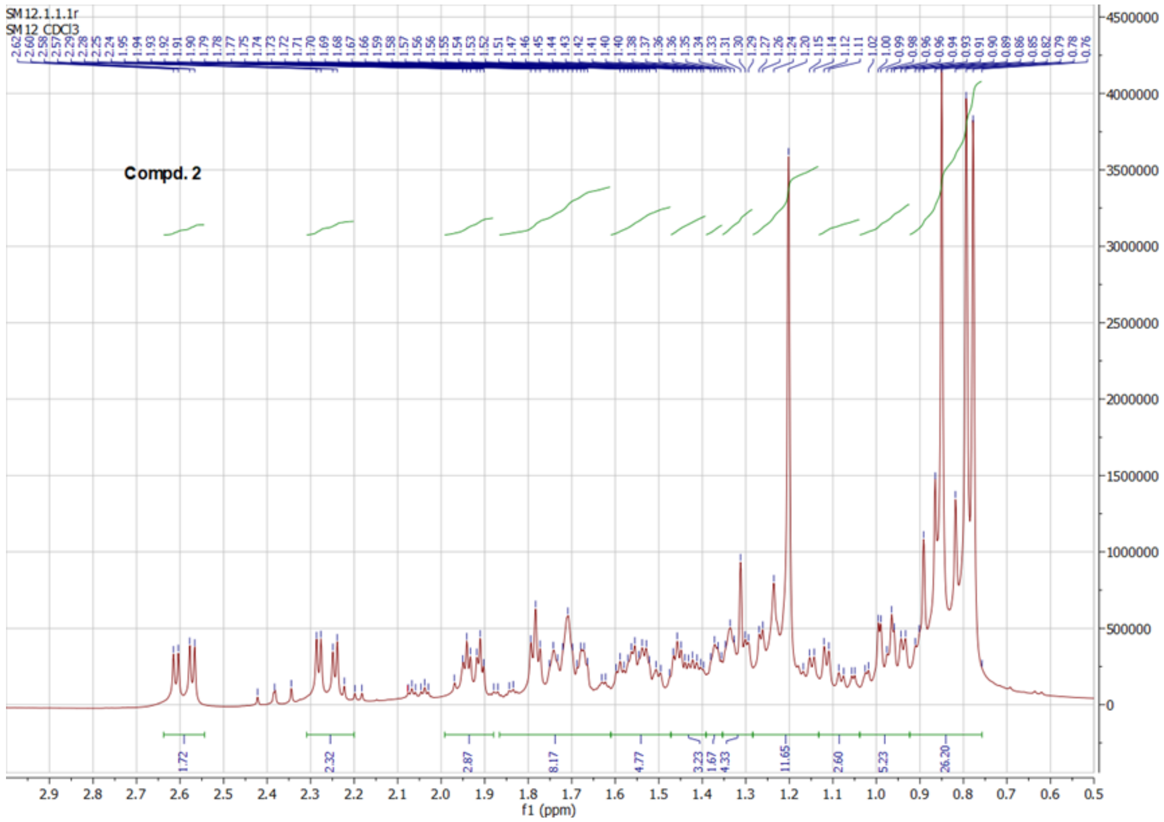
SM12.1.1.1r
SM12 CDCl3
Compd. 2
f1 (ppm)

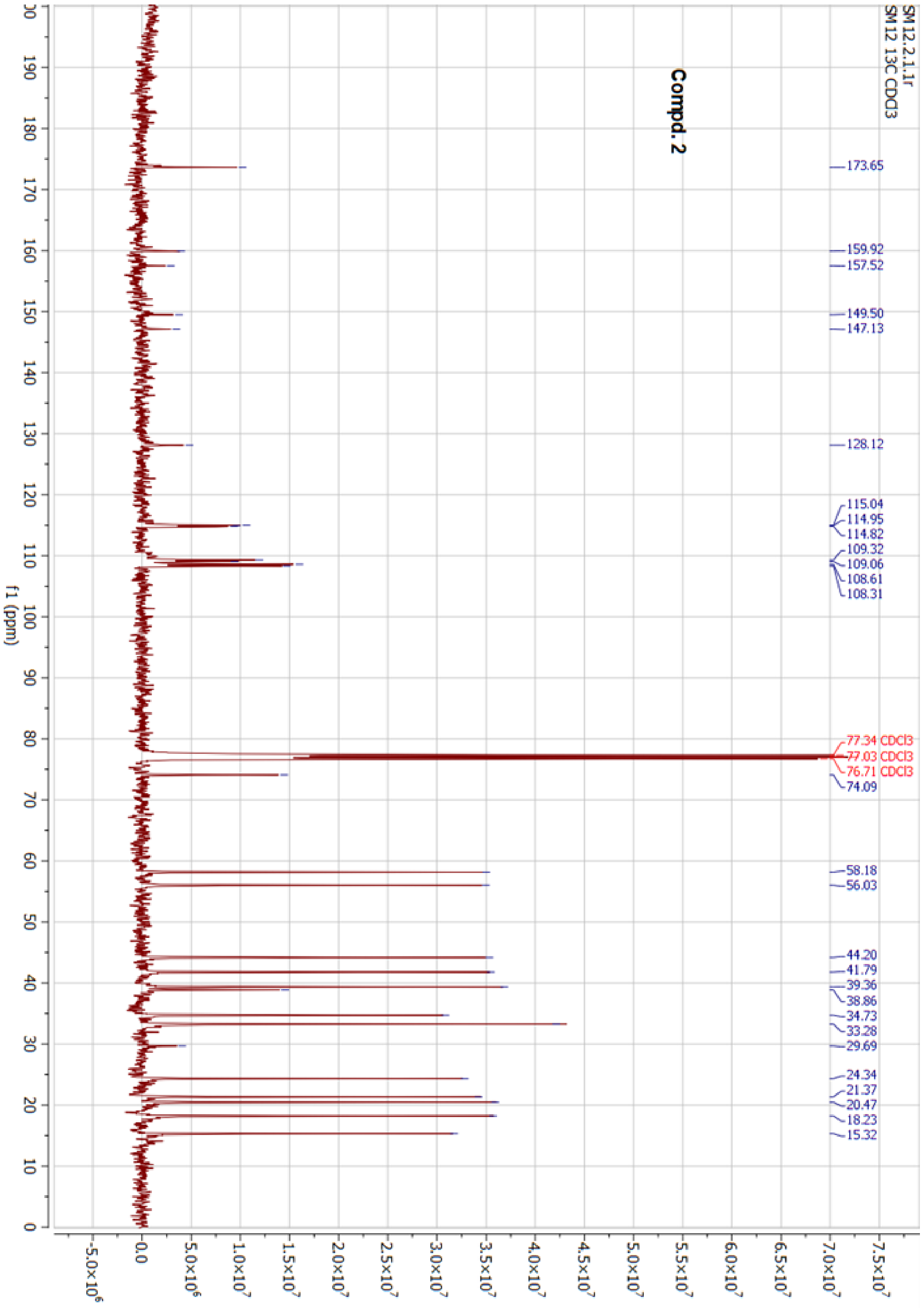

SM 12.2.1.1r
SM 12 13C CDCl3
Compd. 2
173.65
159.92
157.52
149.50
147.13
128.12
115.04
114.95
114.82
109.32
109.06
108.61
108.31
77.34 CDCl3
77.03 CDCl3
76.71 CDCl3
74.09
58.18
56.03
44.20
41.79
39.36
38.86
34.73
33.28
29.69
24.34
21.37
20.47
18.23
15.32
f1 (ppm)

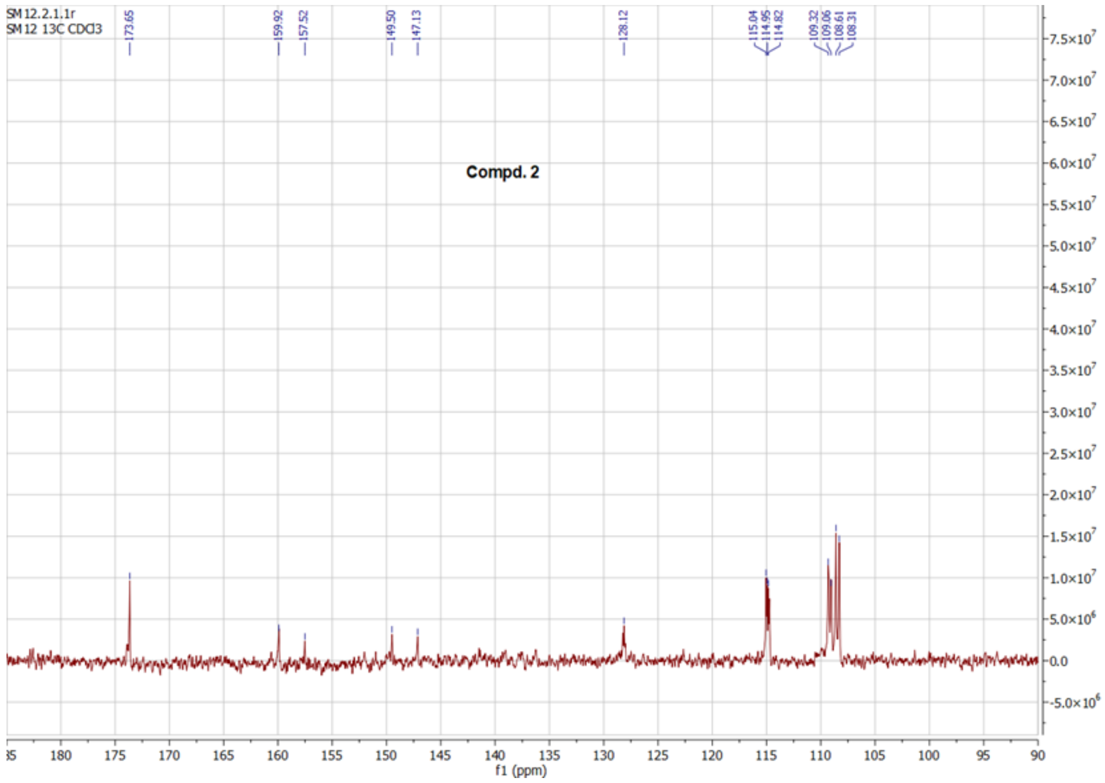
SM12.2.1.1r
SM12 13C CDCl3
173.65
159.92
157.52
149.50
147.13
128.12
115.04
114.95
114.82
109.32
109.06
108.61
108.31
Compd. 2
f1 (ppm)

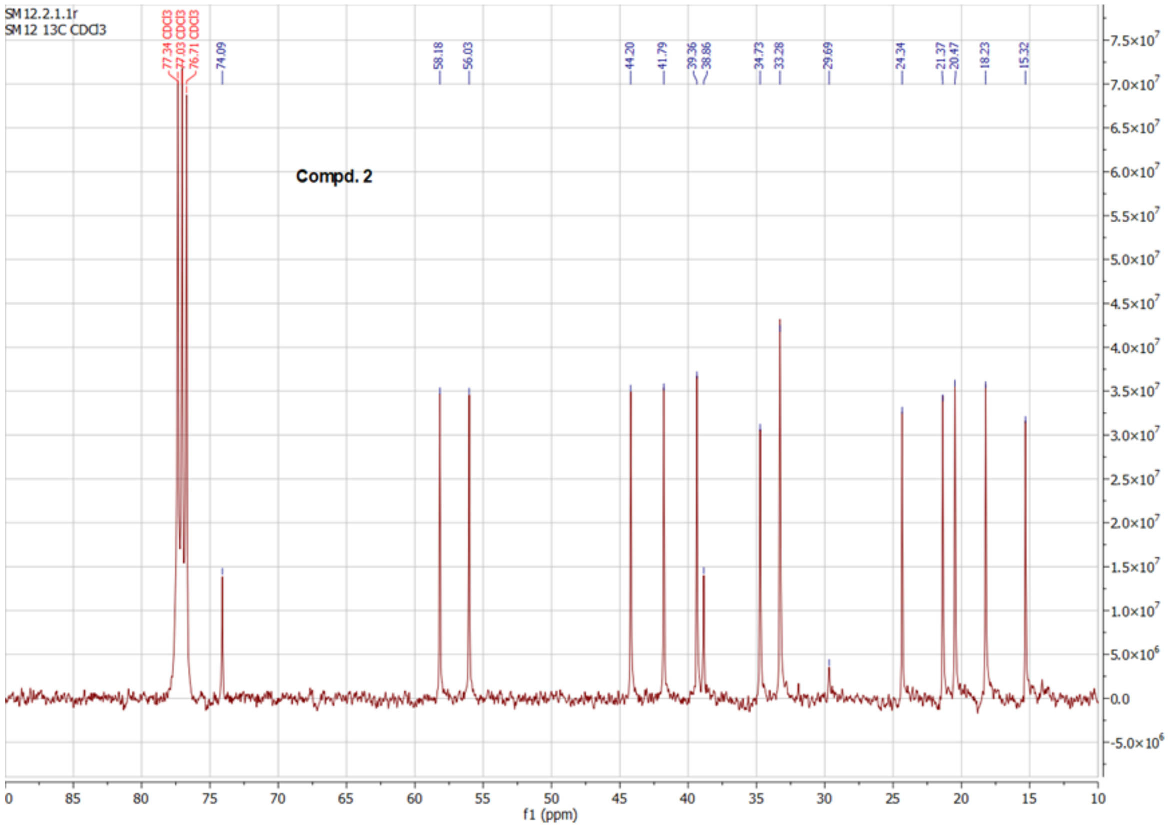
SM12.2.1.1r
SM12 13C CDCl3
77.34 CDCl3
77.03 CDCl3
76.71 CDCl3
74.09
58.18
56.03
44.20
41.79
39.36
38.86
34.73
33.28
29.69
24.34
21.37
20.47
18.23
15.32
Compd. 2
f1 (ppm)

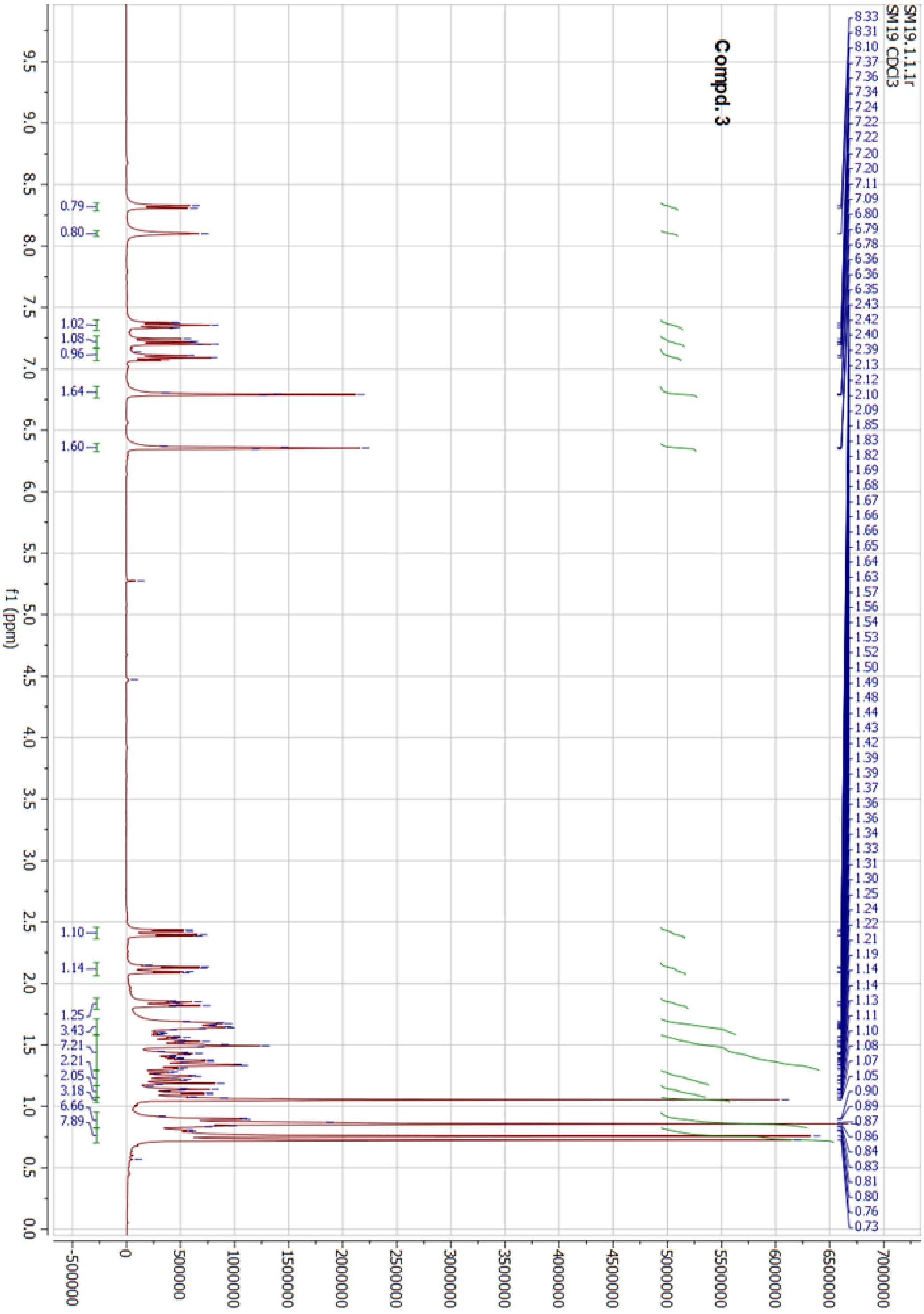
SM19.1.1.1r
SM19 CDCl3
Compd.3
f1 (ppm)

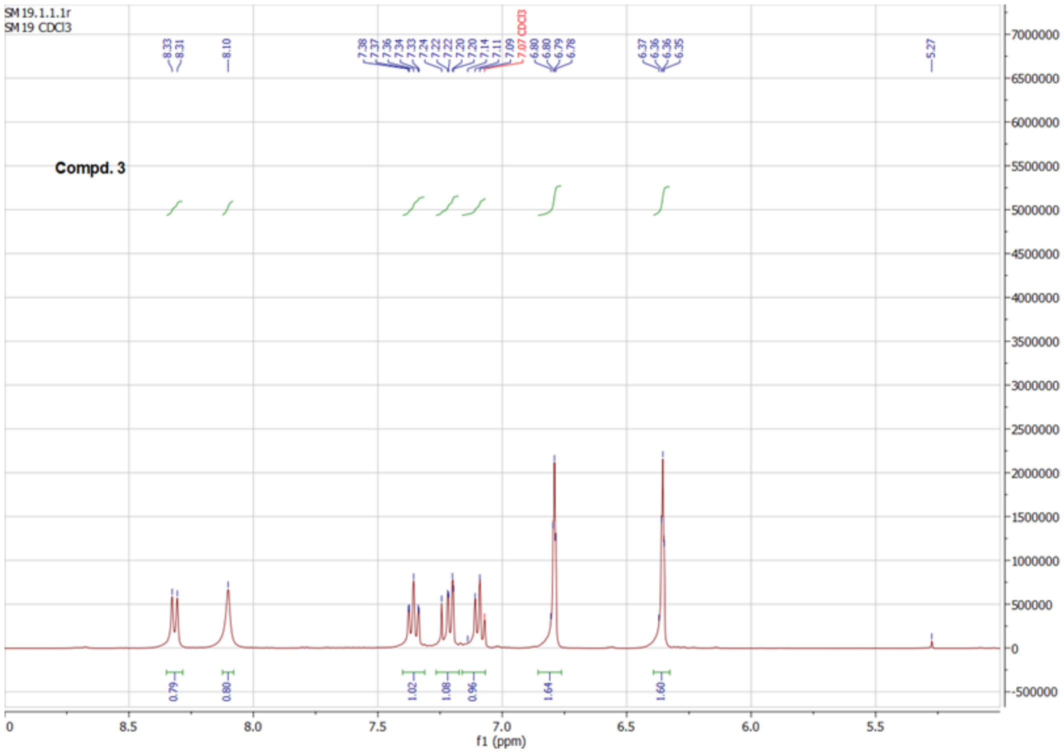
SM19.1.1.1r
SM19 CDCl3
Compd. 3
f1 (ppm)

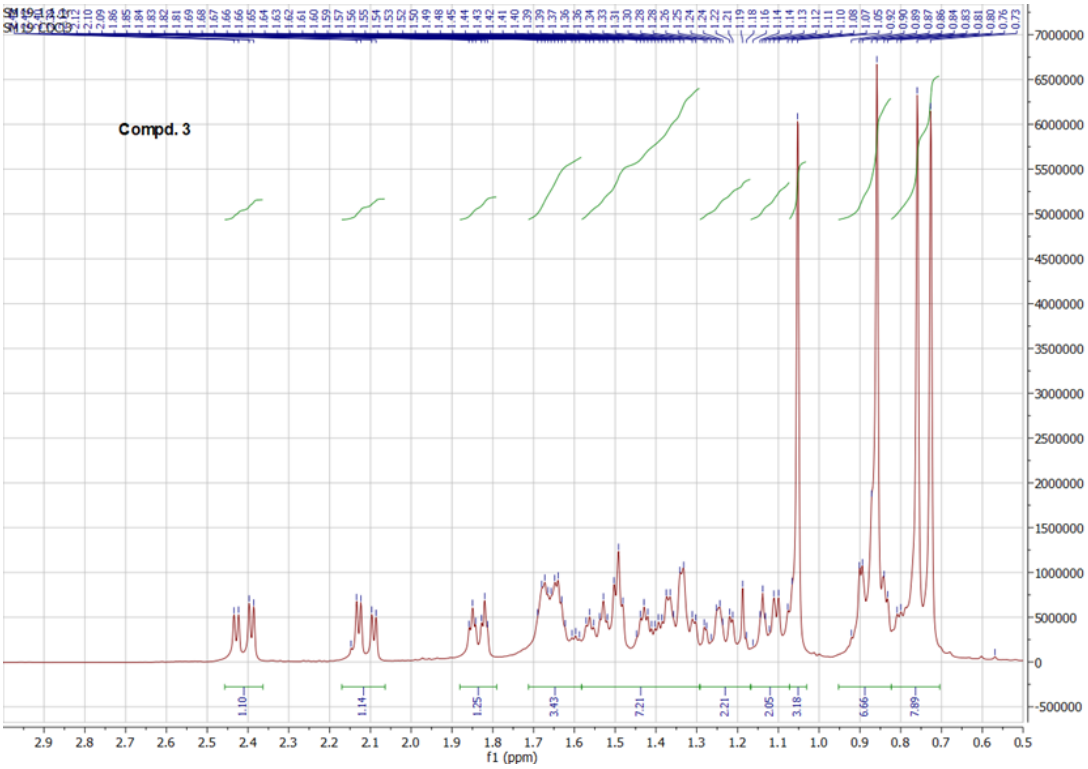
Compd. 3
f1 (ppm)

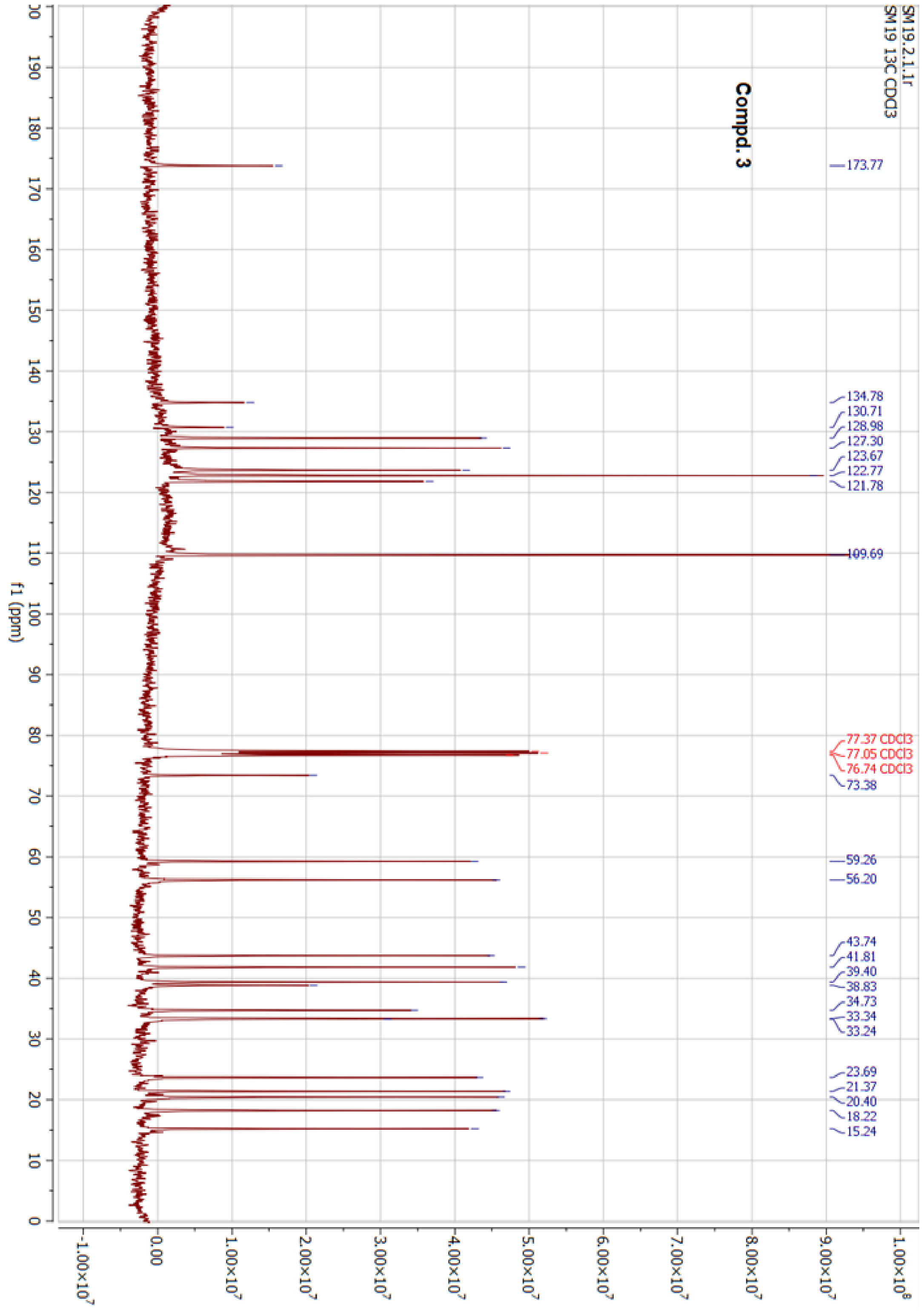
SM19.2.1.1r
SM19 13C CDCl3
Compd. 3
173.77
134.78
130.71
128.98
127.30
123.67
122.77
121.78
109.69
77.37 CDCl3
77.05 CDCl3
76.74 CDCl3
73.38
59.26
56.20
43.74
41.81
39.40
38.83
34.73
33.34
33.24
23.69
21.37
20.40
18.22
15.24
f1 (ppm)

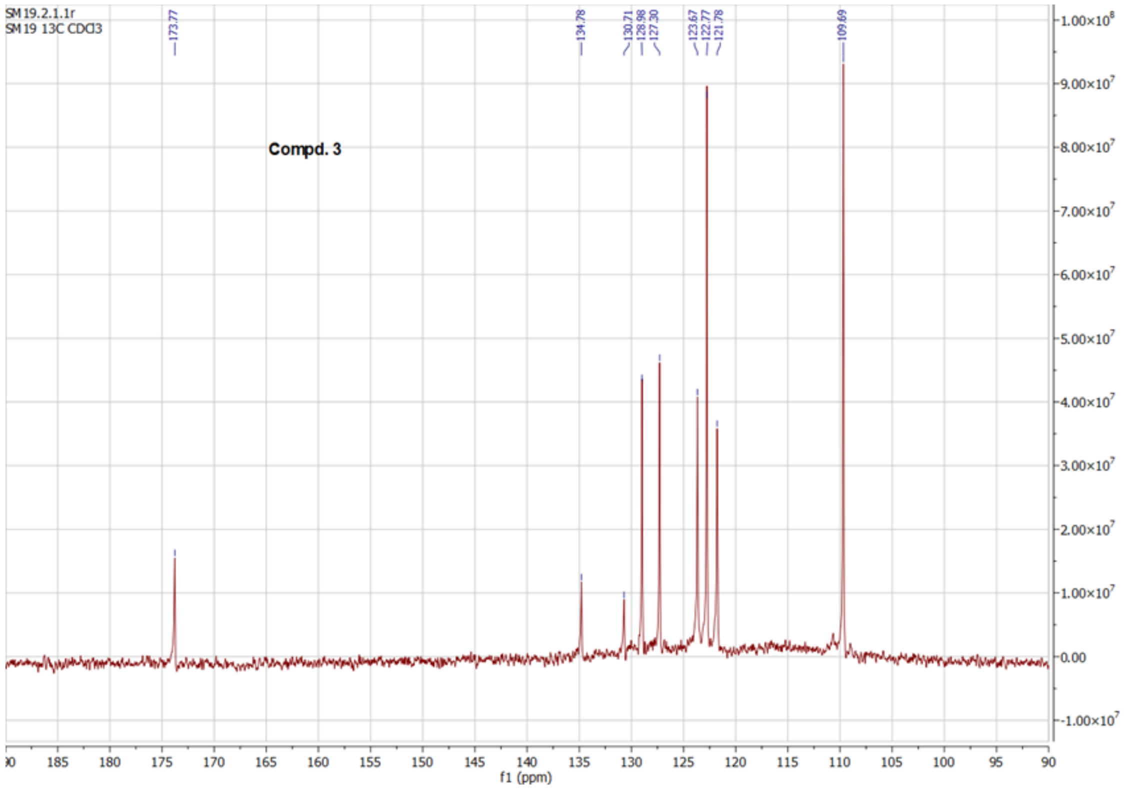
SM 19.2.1.1r
SM 19 13C CDCl3
173.77
134.78
130.71
128.98
127.30
123.67
122.77
121.78
109.69
Compd. 3
f1 (ppm)

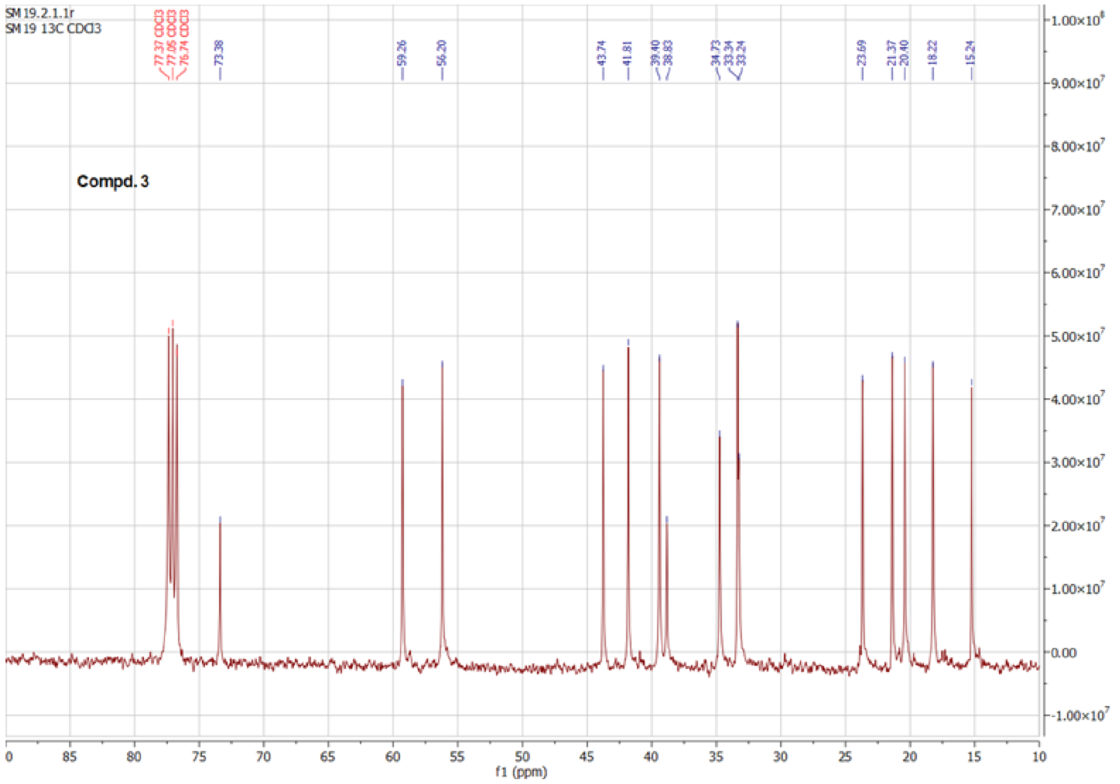
SM 19.2.1.1r
SM 19 13C CDCl3
77.37 CDCl3
77.05 CDCl3
76.74 CDCl3
73.38
59.26
56.20
43.74
41.81
39.40
38.83
34.73
33.34
33.24
23.69
21.37
20.40
18.22
15.24
Compd. 3
f1 (ppm)

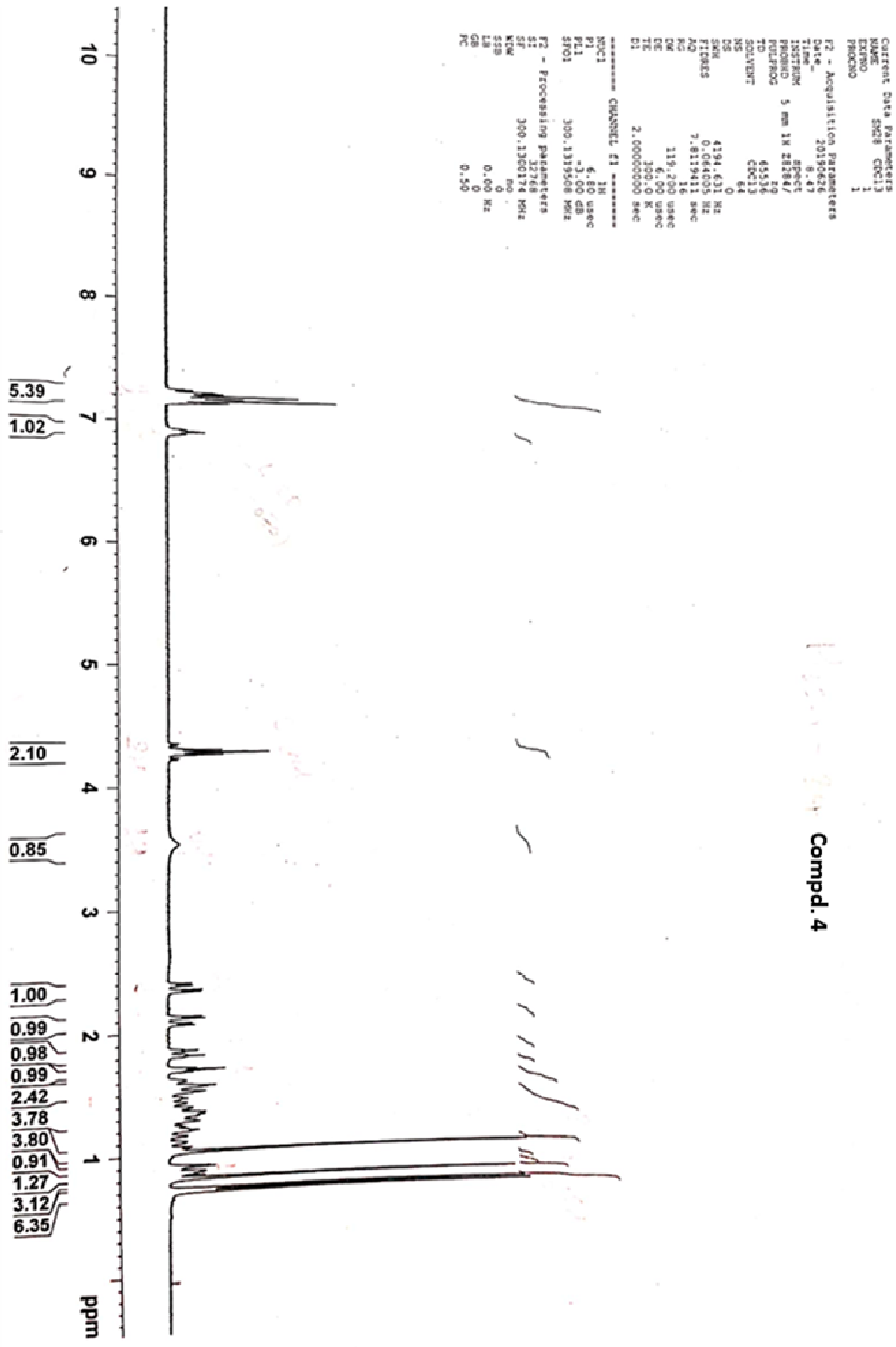
Compd. 4
ppm

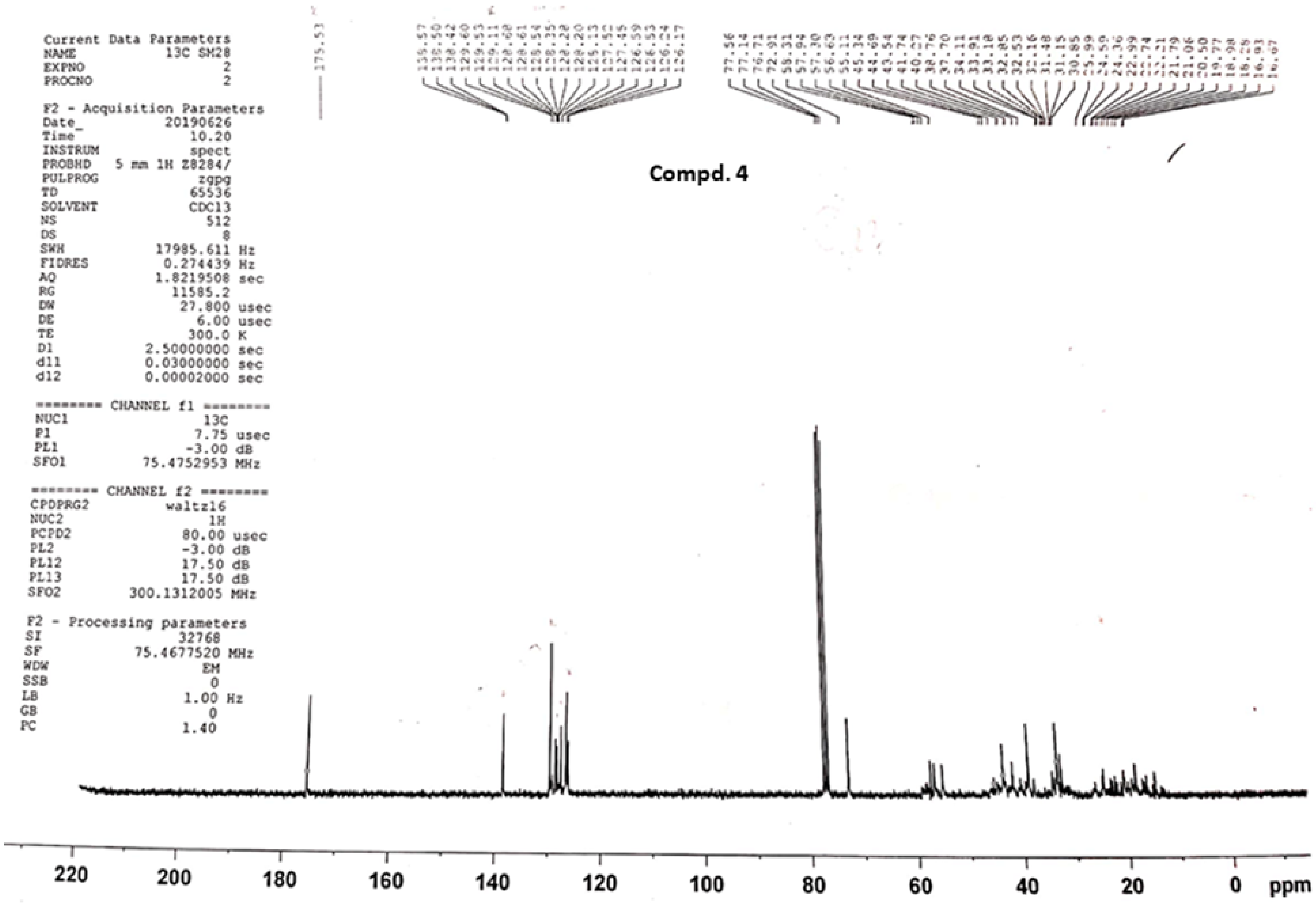
Current Data Parameters
NAME 13C SM28
EXPNO 2
PROCNO 2
F2 - Acquisition Parameters
Date_ 20190626
Time_ 10.20
INSTRUM spect
PROBHD 5 mm 1H Z8284/
PULPROG zgpg
TD 65536
SOLVENT CDCl3
NS 512
DS 8
SWH 17985.611 Hz
FIDRES 0.274439 Hz
AQ 1.8219508 sec
RG 11585.2
DW 27.800 usec
DE 6.00 usec
TE 300.0 K
D1 2.50000000 sec
d11 0.03000000 sec
d12 0.00002000 sec
======== CHANNEL f1 ========
NUC1 13C
P1 7.75 usec
PL1 -3.00 dB
SFO1 75.4752953 MHz
======== CHANNEL f2 ========
CPDPRG2 waltz16
NUC2 1H
PCPD2 80.00 usec
PL2 -3.00 dB
PL12 17.50 dB
PL13 17.50 dB
SFO2 300.1312005 MHz
F2 - Processing parameters
SI 32768
SF 75.4677520 MHz
WDW EM
SSB 0
LB 1.00 Hz
GB 0
PC 1.40
175.53
138.57
138.50
138.42
129.60
129.53
129.11
128.68
128.61
128.54
128.35
128.28
128.20
128.13
127.52
127.45
126.59
126.53
126.24
126.17
77.56
77.14
76.71
72.91
58.31
57.94
57.30
56.63
55.11
45.34
44.69
43.54
41.74
40.27
38.76
37.70
34.11
33.91
33.18
32.85
32.53
32.16
31.48
31.15
30.85
25.99
24.59
24.36
22.99
22.74
21.79
21.06
20.50
19.77
18.98
18.28
16.93
16.67
Compd. 4
220
200
180
160
140
120
100
80
60
40
20
0
ppm

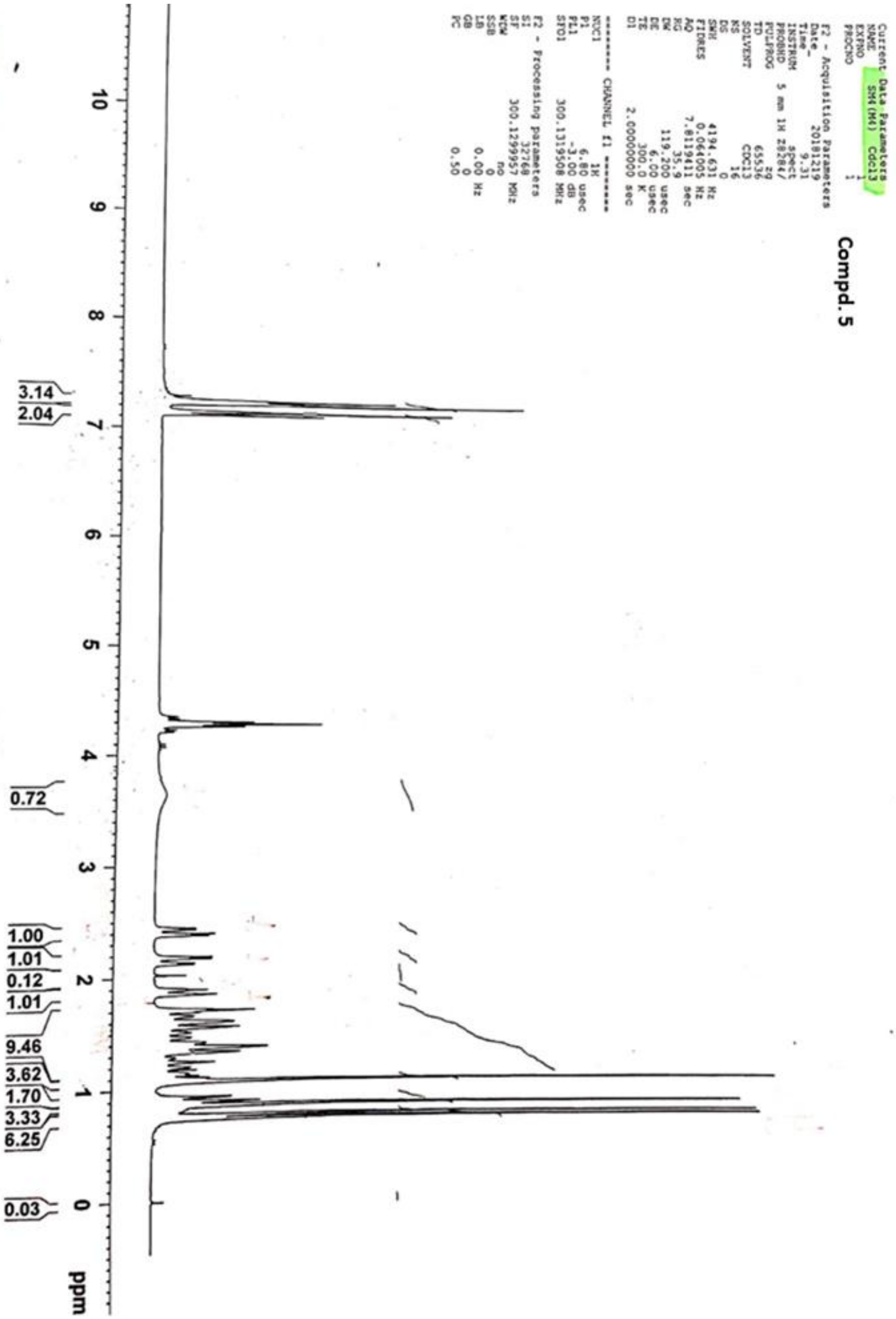
Compd. 5

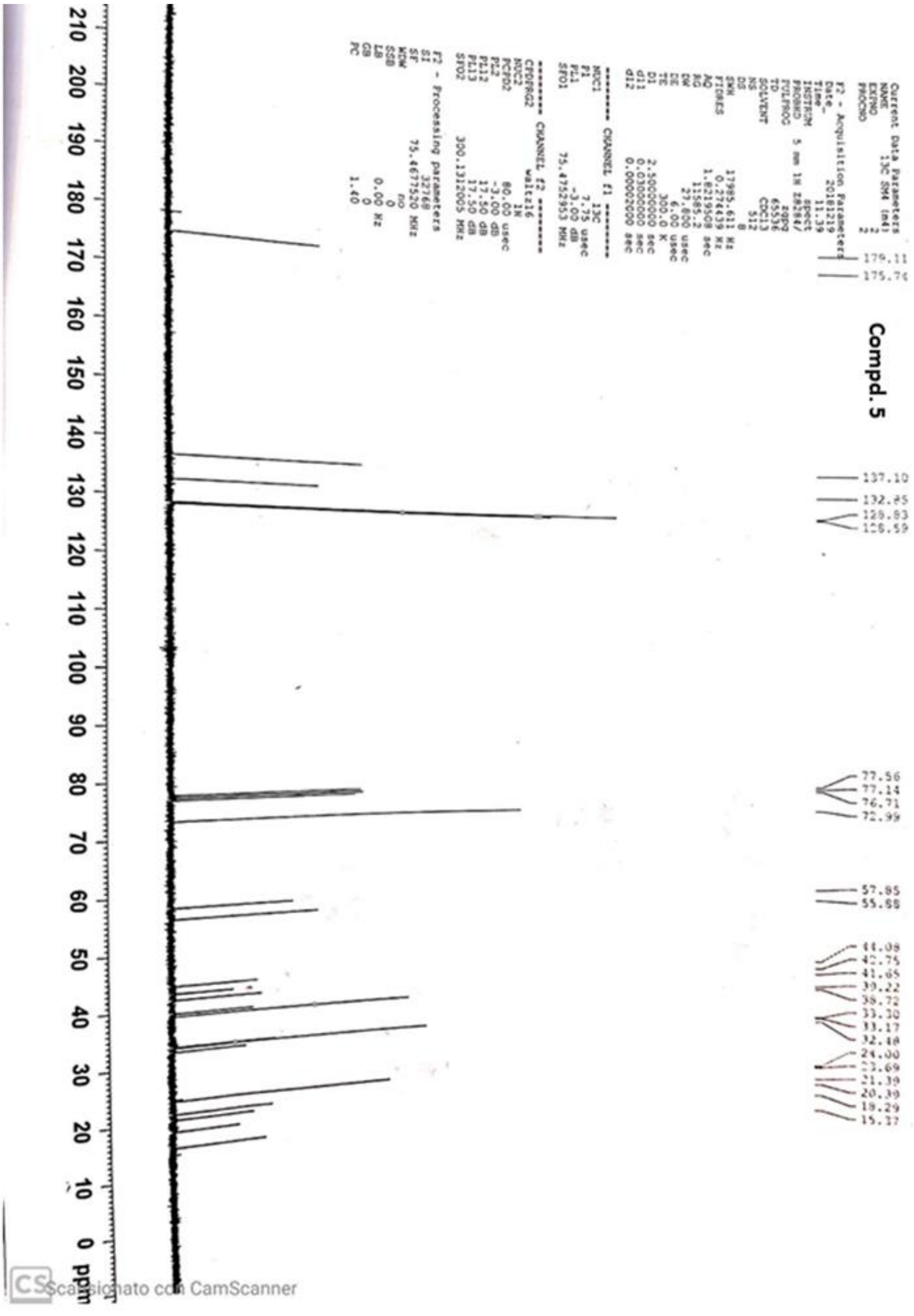
Compd. 5

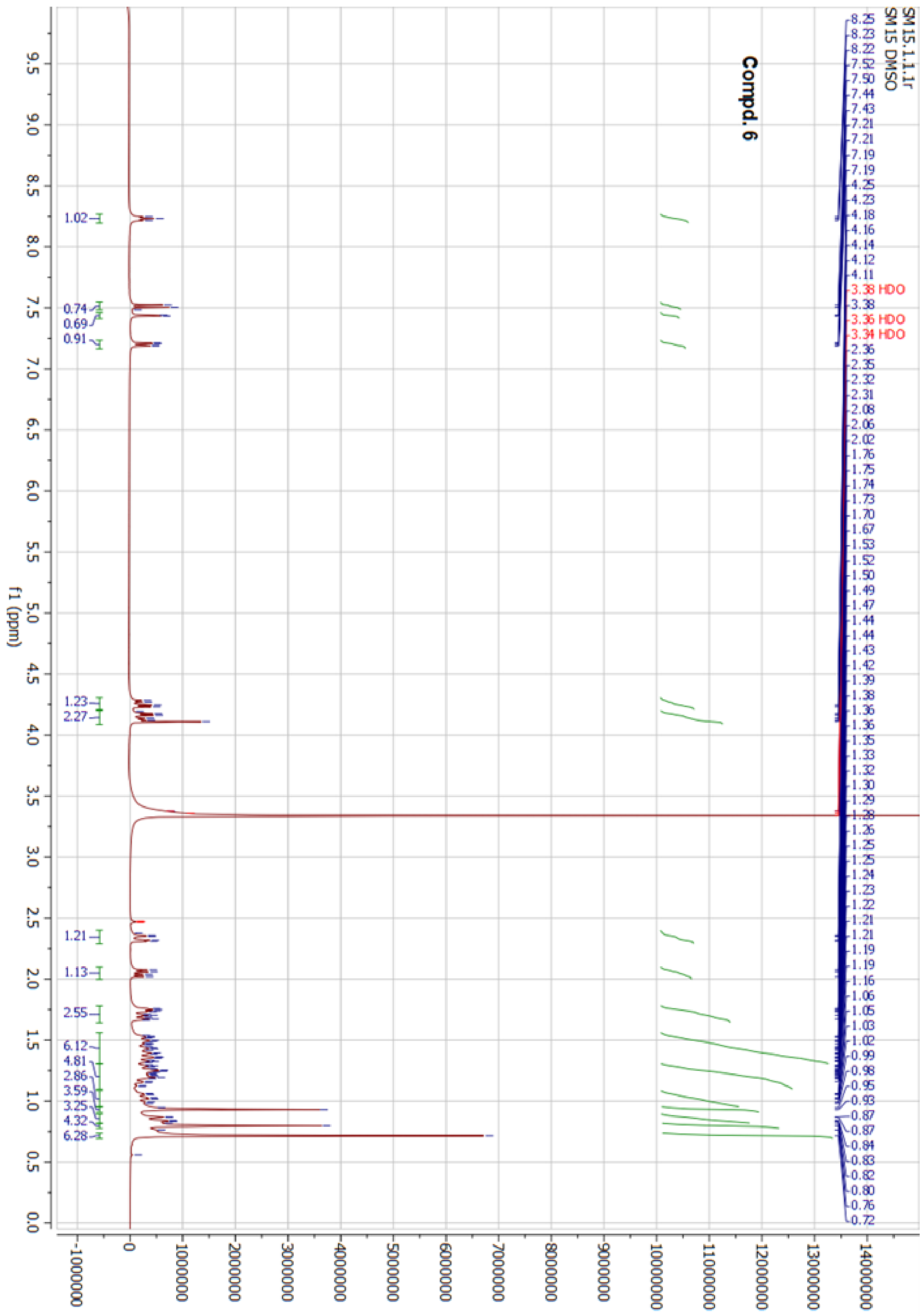
Compd. 6
SM15.1.1.fr
SM15 DMSO
f1 (ppm)

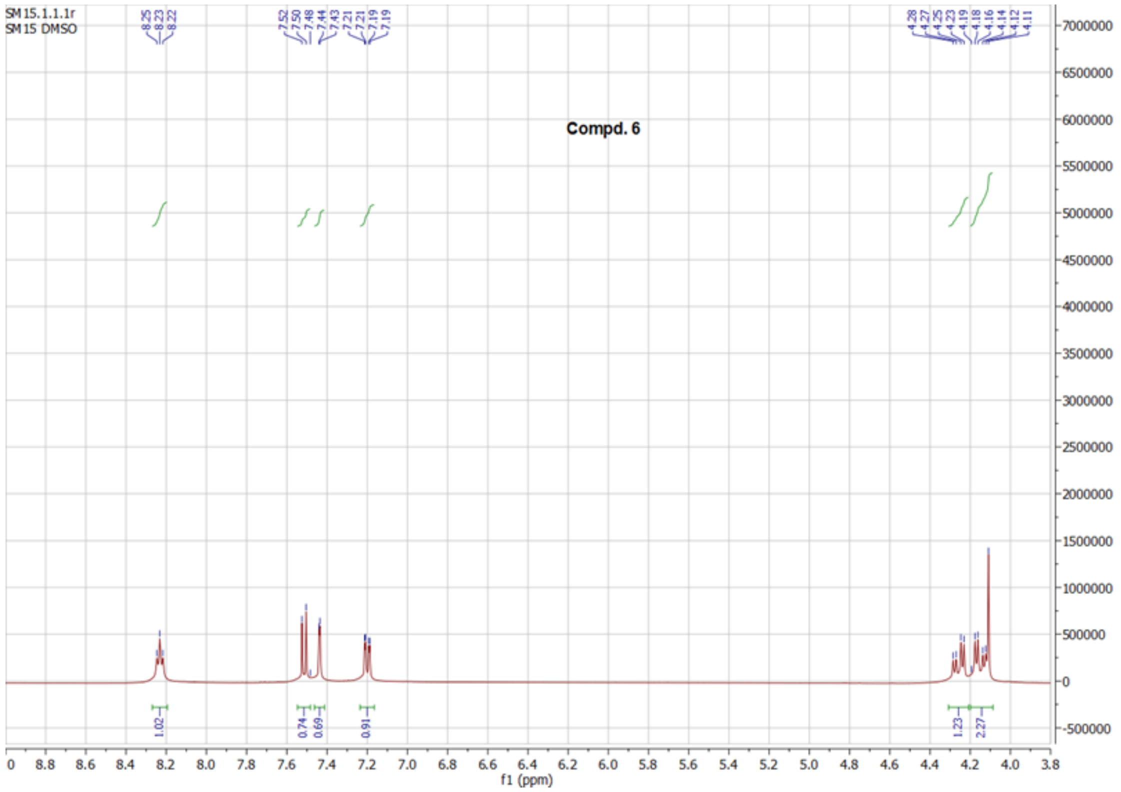
SM15.1.1.1r
SM15 DMSO
Compd. 6
f1 (ppm)

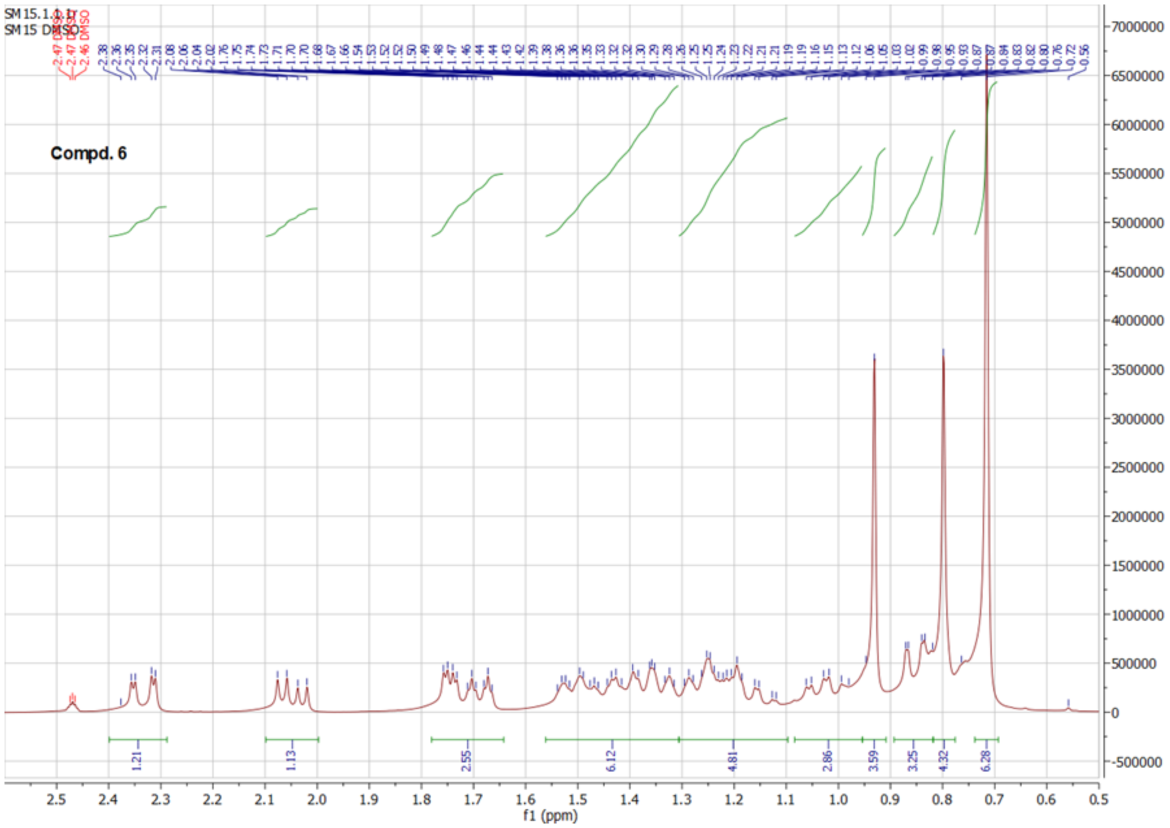
SM15.1.1.1r
SM15 DMSO
Compd. 6
f1 (ppm)

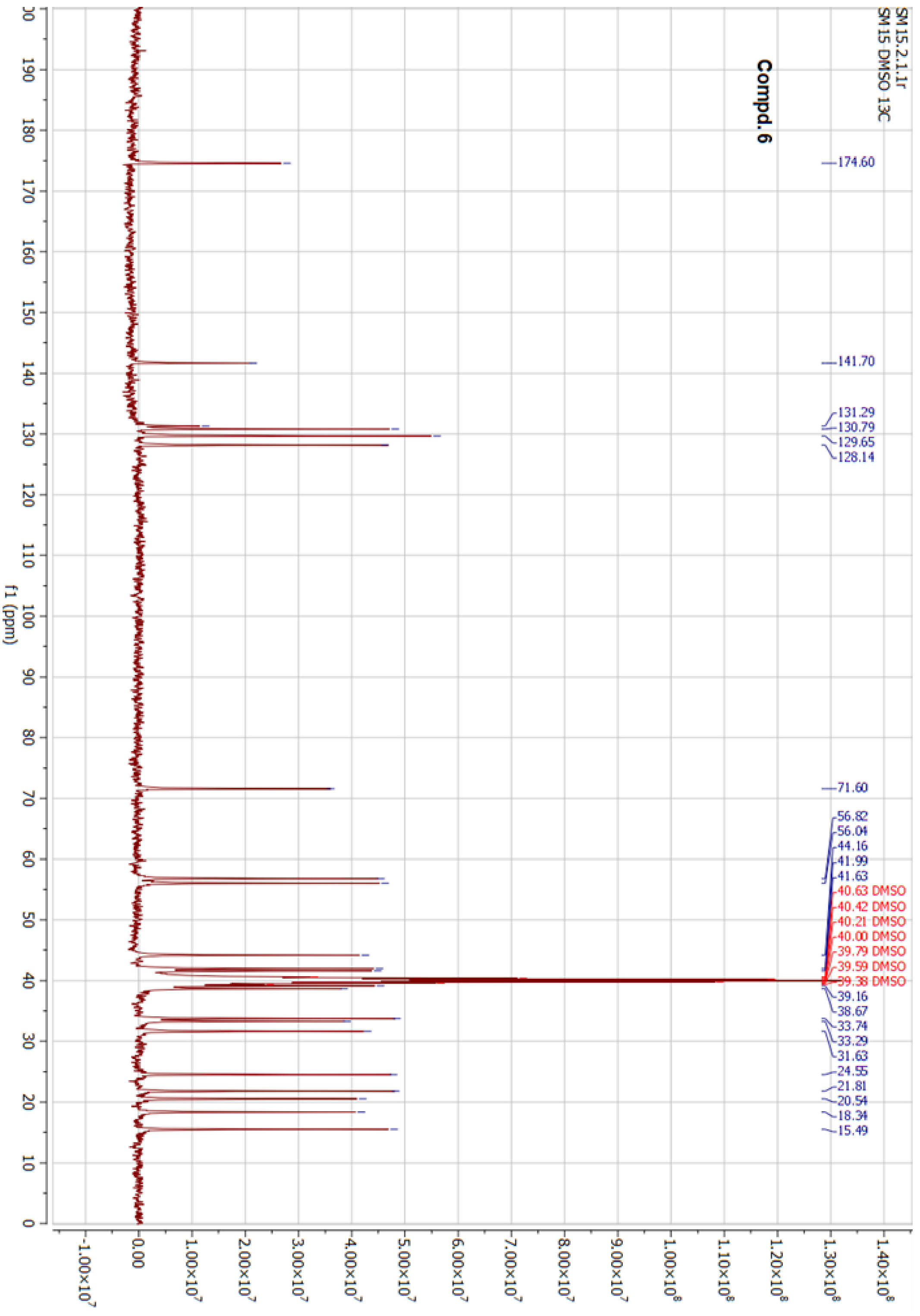
SM15.2.1.1r
SM15 DMSO 13C
Compd.6
174.60
141.70
131.29
130.79
129.65
128.14
71.60
56.82
56.04
44.16
41.99
41.63
40.63 DMSO
40.42 DMSO
40.21 DMSO
40.00 DMSO
39.79 DMSO
39.59 DMSO
39.38 DMSO
39.16
38.67
33.74
33.29
31.63
24.55
21.81
20.54
18.34
15.49
f1 (ppm)

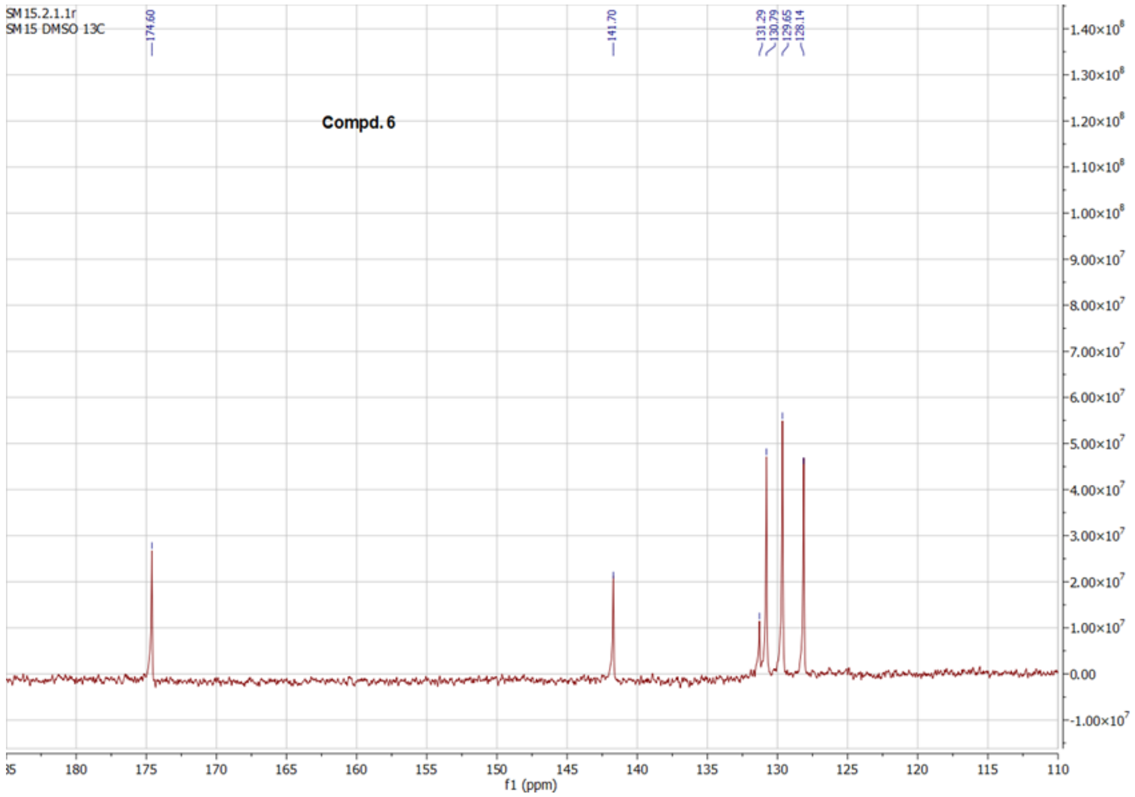
SM15.2.1.1r
SM15 DMSO 13C
Compd. 6
174.60
141.70
131.29
130.79
129.65
128.14
f1 (ppm)

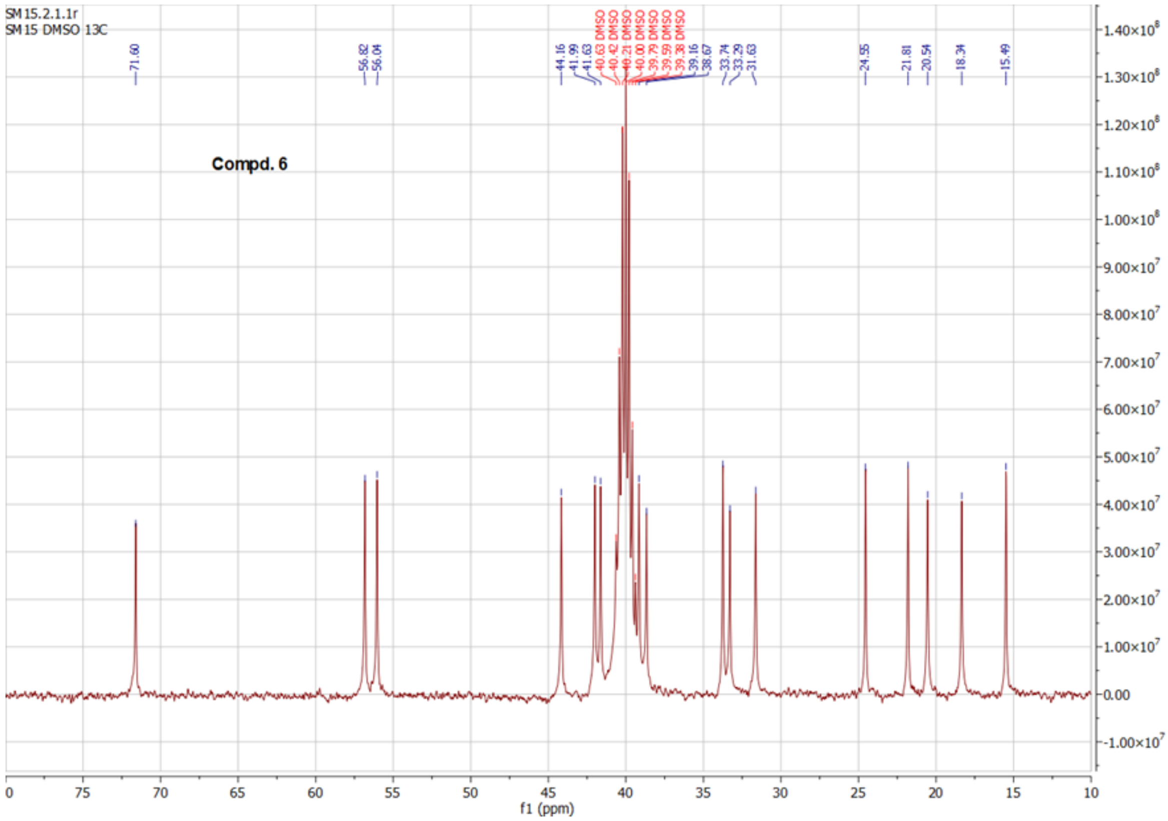
SM15.2.1.1r
SM15 DMSO 13C
Compd. 6
71.60
56.82
56.04
44.16
41.99
41.63
33.74
33.29
31.63
24.55
21.81
20.54
18.34
15.49
f1 (ppm)

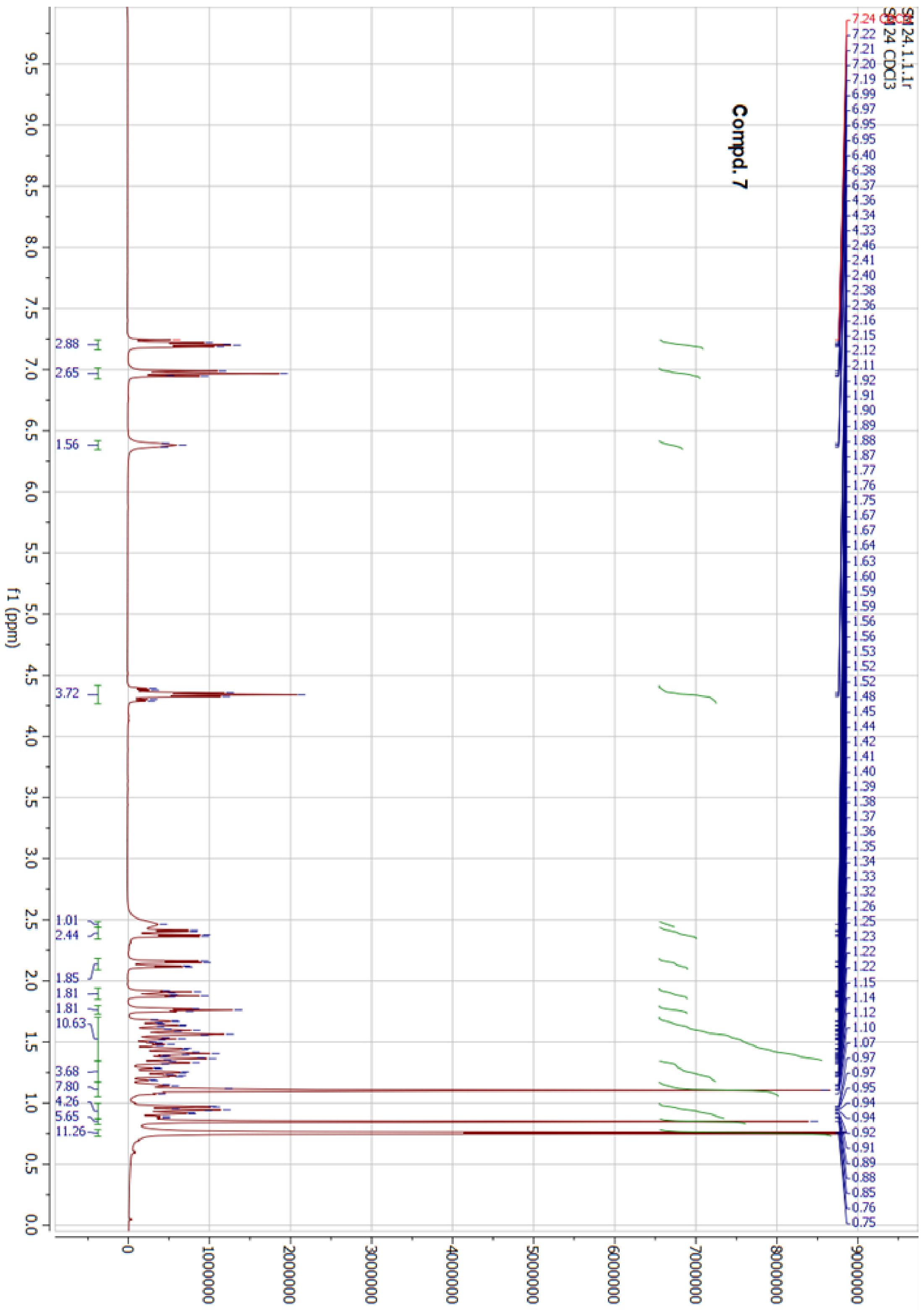
Compd. 7
f1 (ppm)

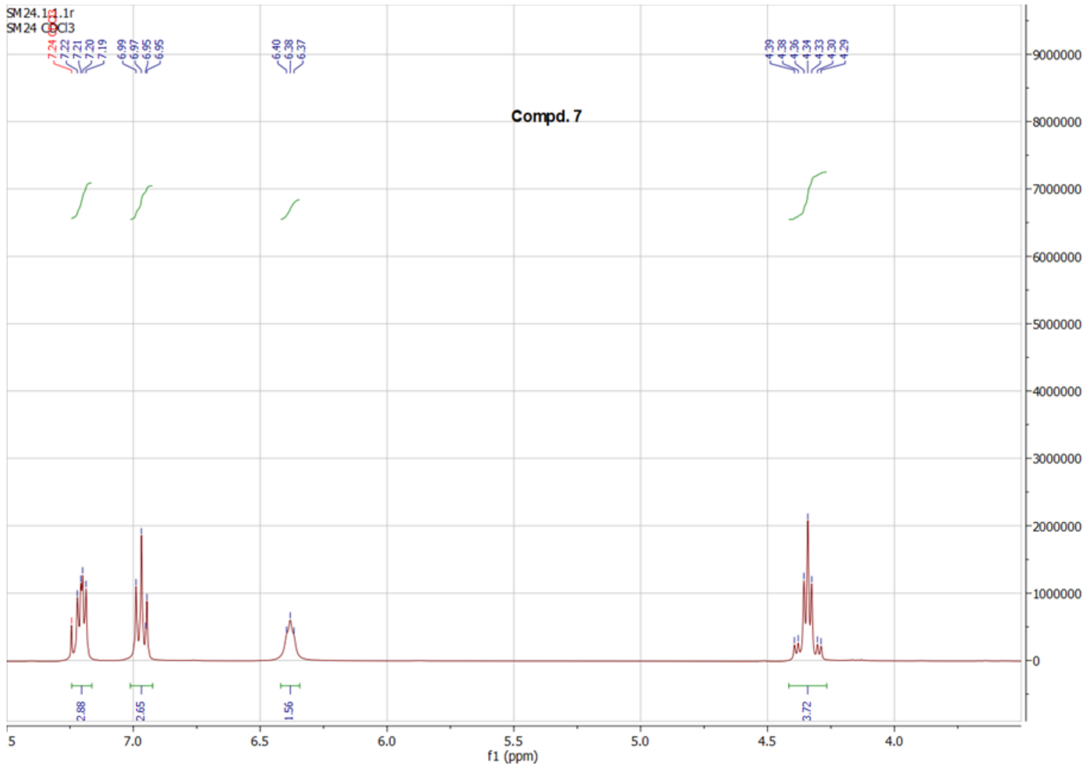
Compd. 7

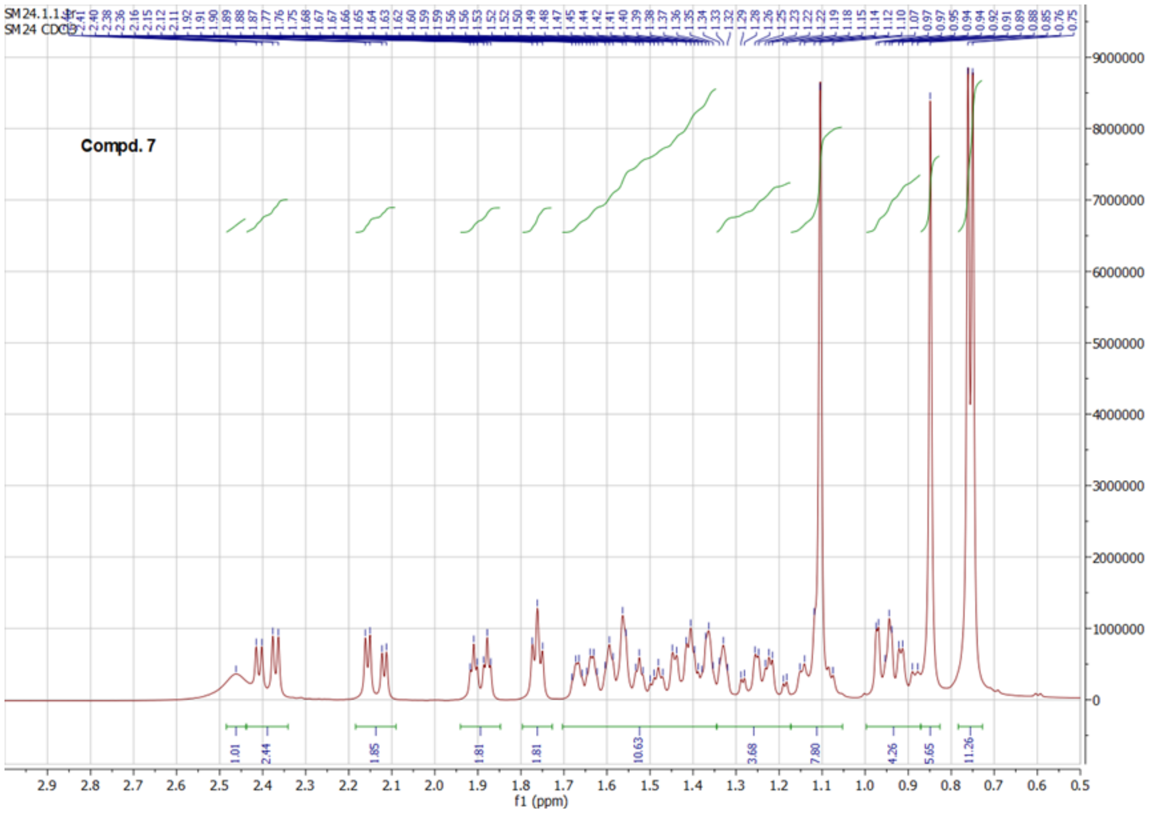
Compd. 7

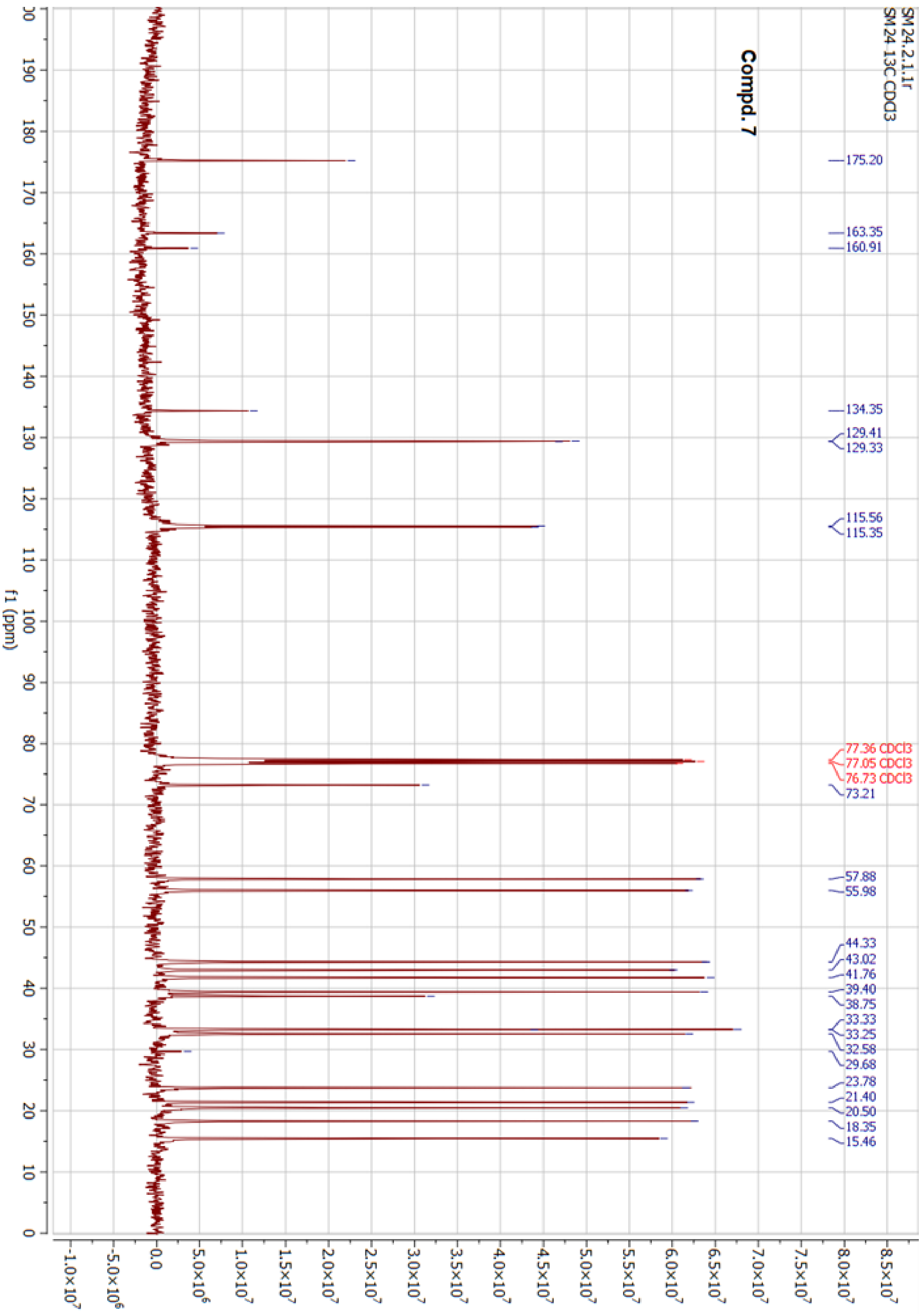
SM24.2.1.1r
SM24 13C CDCl3
Compd. 7
175.20
163.35
160.91
134.35
129.41
129.33
115.56
115.35
77.36 CDCl3
77.05 CDCl3
76.73 CDCl3
73.21
57.88
55.98
44.33
43.02
41.76
39.40
38.75
33.33
33.25
32.58
29.68
23.78
21.40
20.50
18.35
15.46
f1 (ppm)

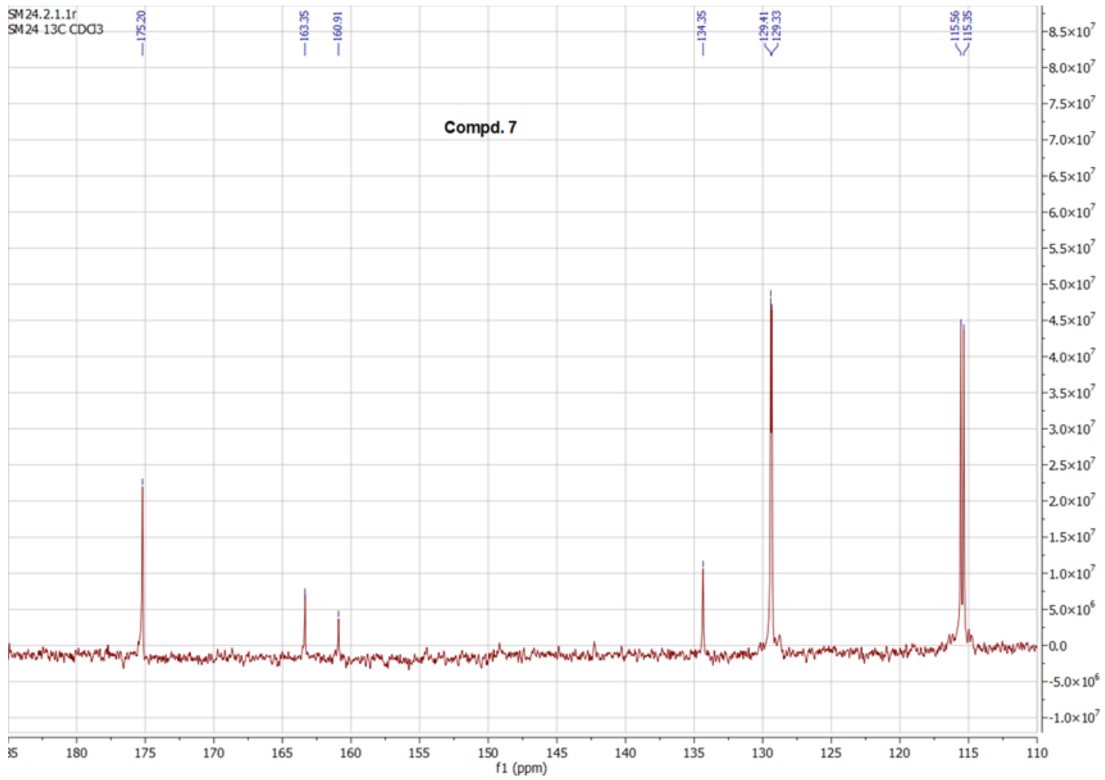
SM24.2.1.1r
SM24 13C CDCl3
Compd. 7
175.20
163.35
160.91
134.35
129.41
129.33
115.56
115.35
f1 (ppm)

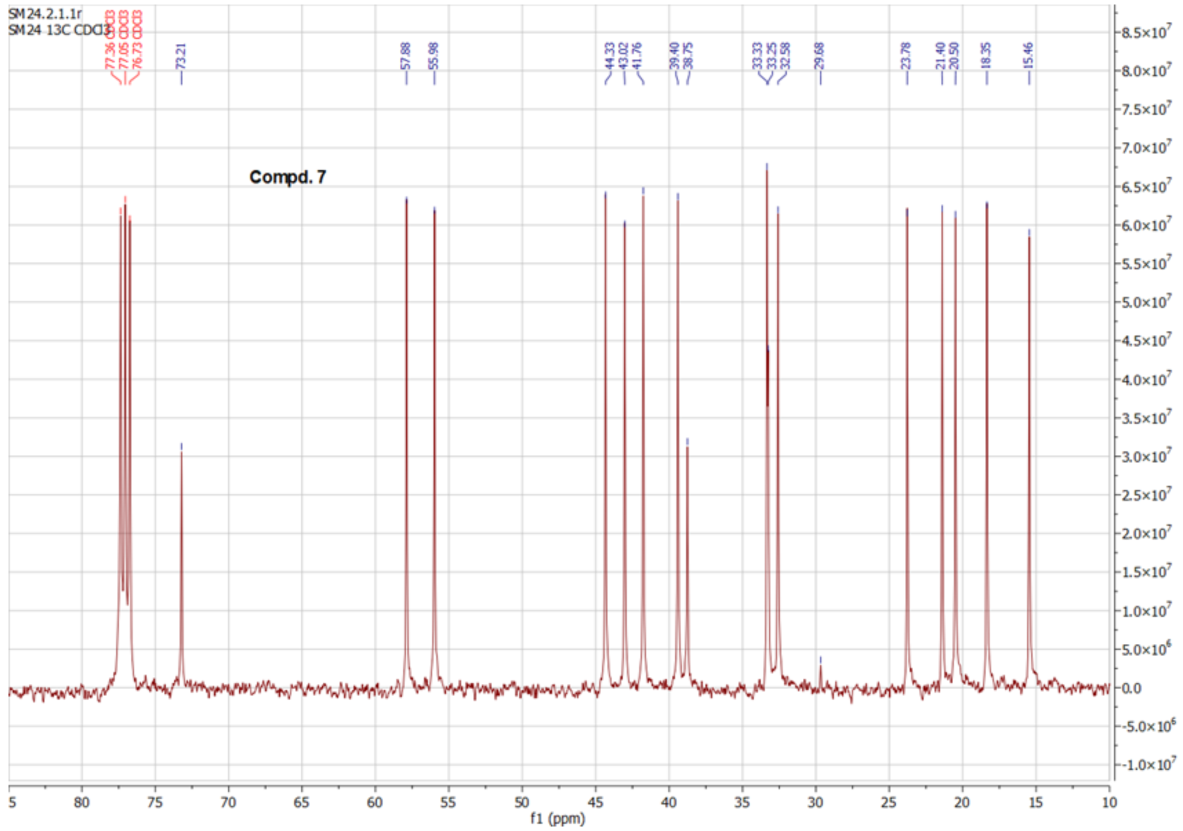
SM24.2.1.1r
SM24 13C CDCl3
Compd. 7
77.36 CDCl3
77.05 CDCl3
76.73 CDCl3
73.21
57.88
55.98
44.33
43.02
41.76
39.40
38.75
33.33
33.25
32.58
29.68
23.78
21.40
20.50
18.35
15.46
f1 (ppm)

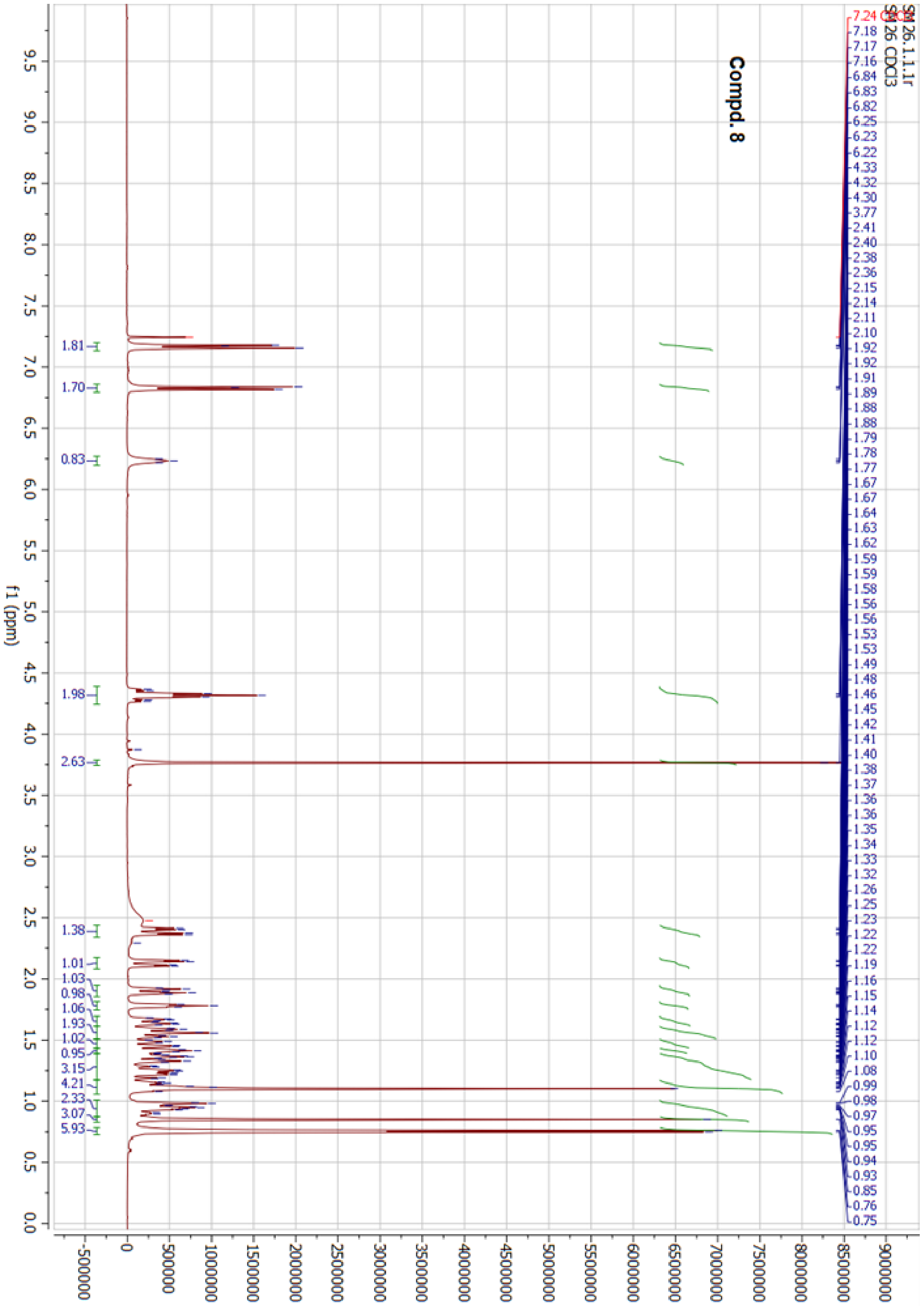

Compd. 8
SM 26.1.1.1r
SM 26 CDCl3
7.24 CDCl3
7.18
7.17
7.16
6.84
6.83
6.82
6.25
6.23
6.22
4.33
4.32
4.30
3.77
2.41
2.40
2.38
2.36
2.15
2.14
2.11
2.10
1.92
1.92
1.91
1.89
1.88
1.88
1.79
1.78
1.77
1.67
1.67
1.64
1.63
1.62
1.59
1.59
1.58
1.56
1.56
1.53
1.53
1.49
1.48
1.46
1.45
1.42
1.41
1.40
1.38
1.37
1.36
1.36
1.35
1.34
1.33
1.32
1.26
1.25
1.23
1.22
1.22
1.19
1.16
1.15
1.14
1.12
1.12
1.10
1.08
0.99
0.98
0.97
0.95
0.95
0.94
0.93
0.85
0.76
0.75
1.81
1.70
0.83
1.98
2.63
1.38
1.01
1.03
0.98
1.06
1.93
1.02
0.95
3.15
4.21
2.33
3.07
5.93
f1 (ppm)

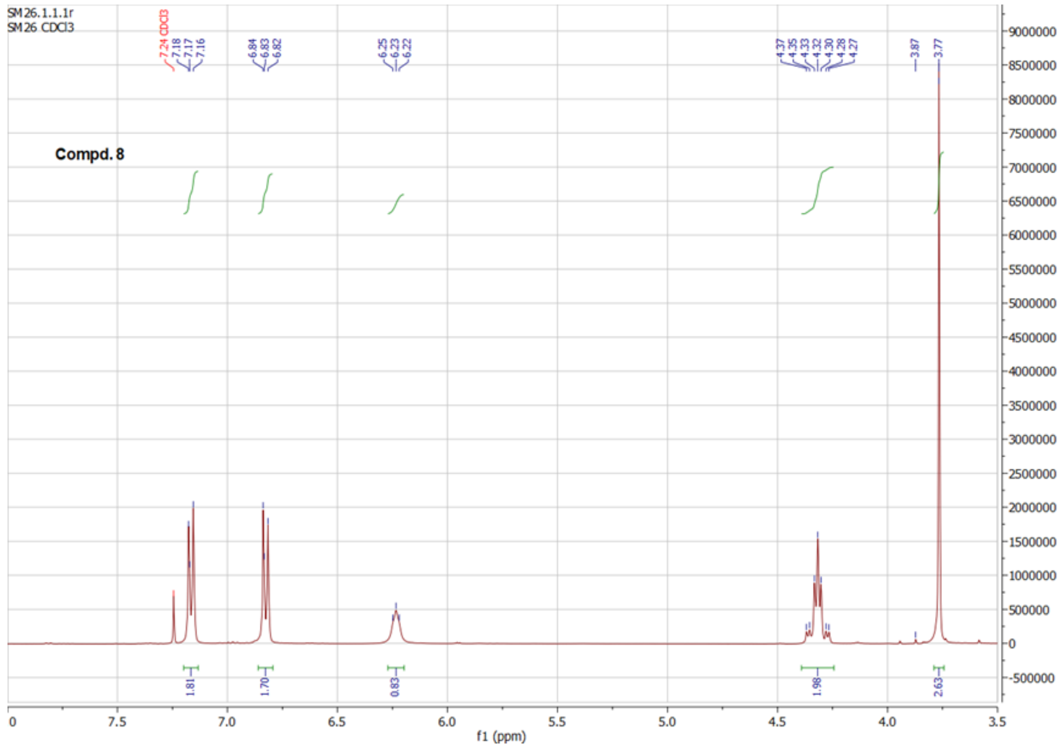
SM26.1.1.1r
SM26 CDCl3
Compd. 8
f1 (ppm)

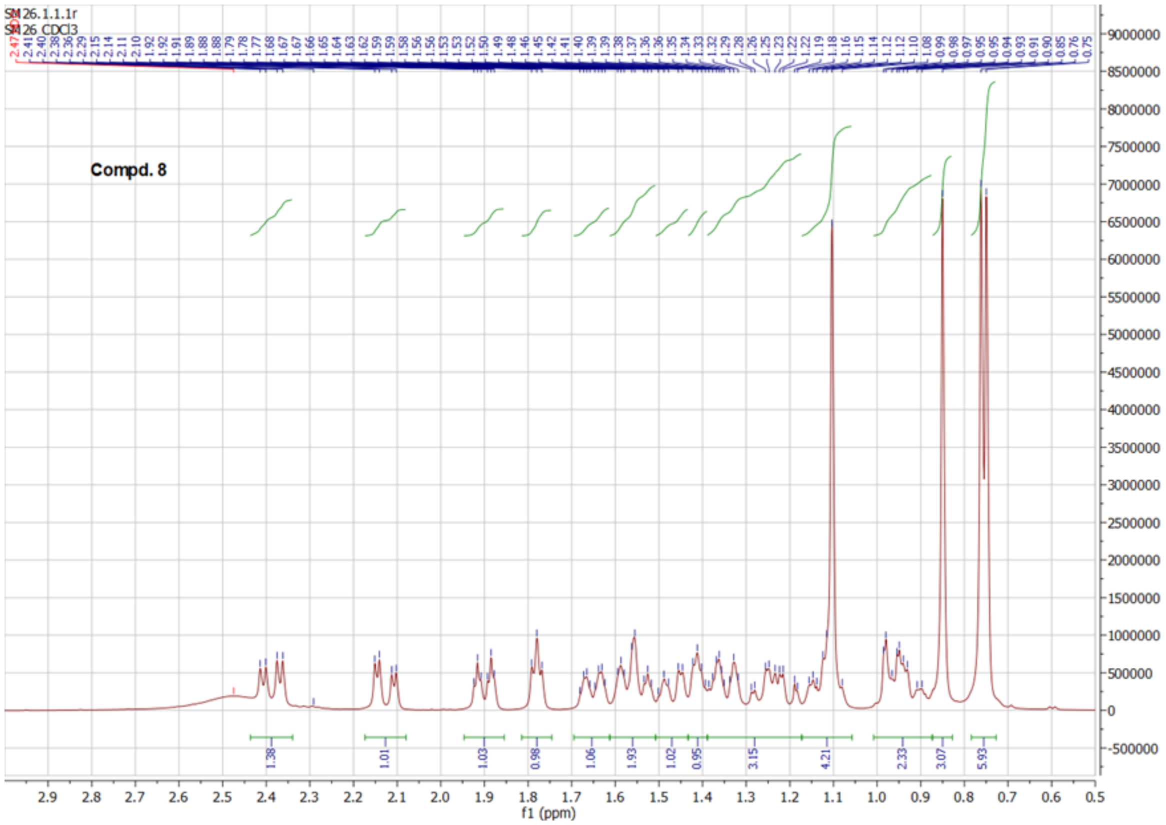
SM26.1.1.1r
SM26 CDCl3
Compd. 8
f1 (ppm)

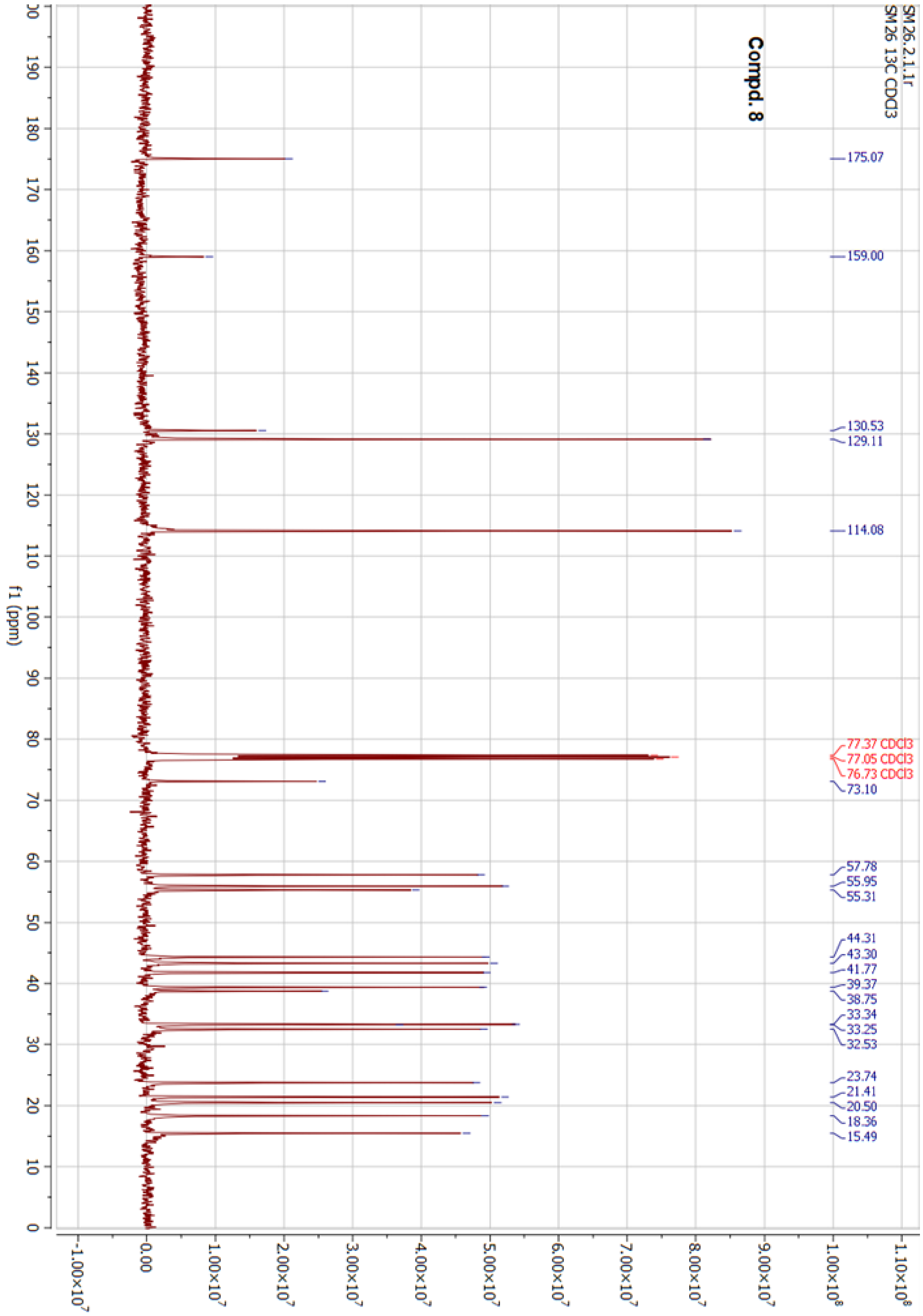
SM26.2.1.1r
SM26 13C CDCl3
Compd. 8
175.07
159.00
130.53
129.11
114.08
77.37 CDCl3
77.05 CDCl3
76.73 CDCl3
73.10
57.78
55.95
55.31
44.31
43.30
41.77
39.37
38.75
33.34
33.25
32.53
23.74
21.41
20.50
18.36
15.49
f1 (ppm)

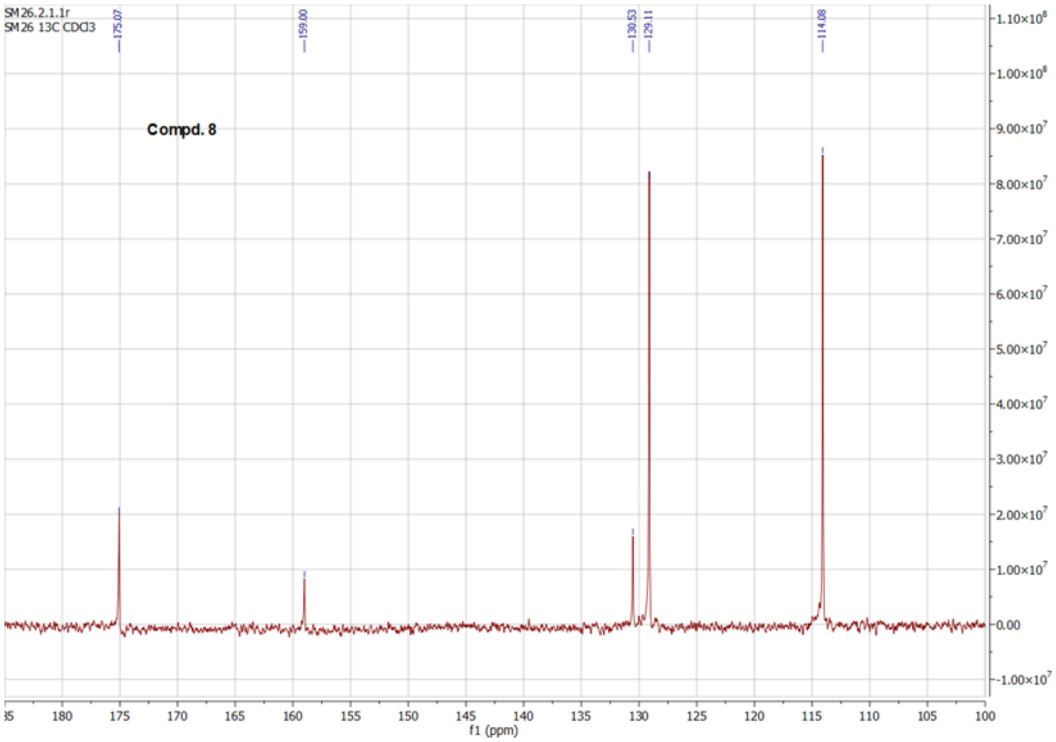
SM26.2.1.1r
SM26 13C CDCl3
Compd. 8
175.07
159.00
130.53
129.11
114.08
f1 (ppm)

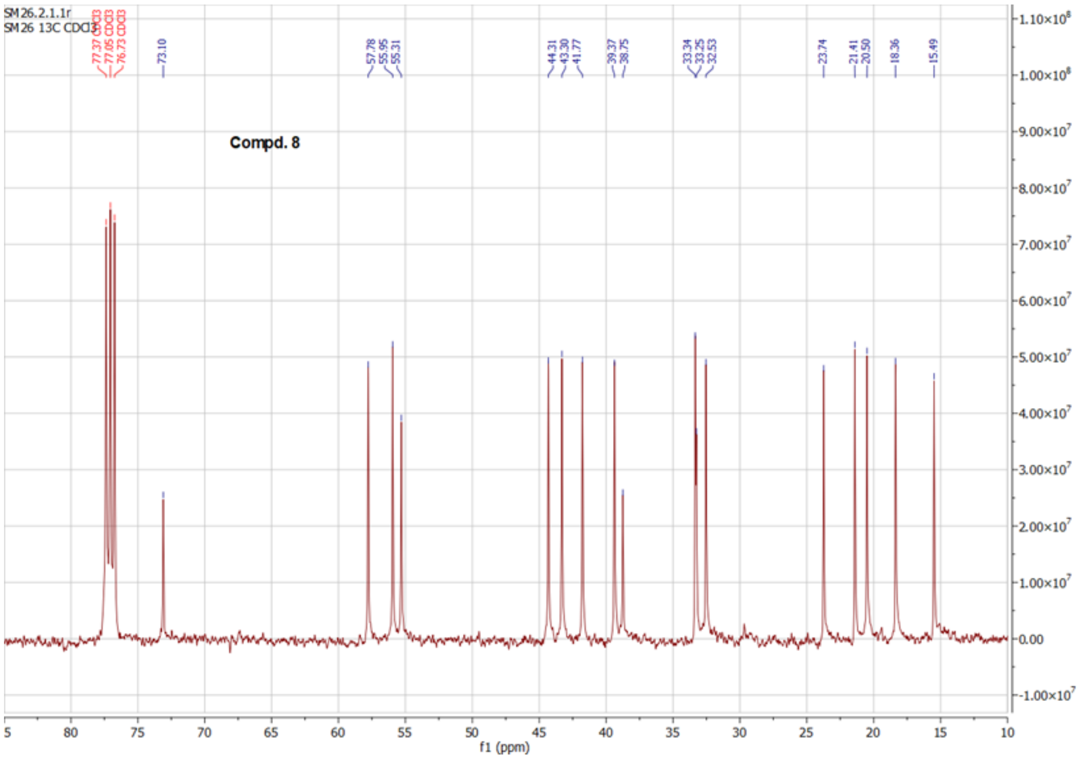
SM26.2.1.1r
SM26 13C CDCl3
Compd. 8
77.37 CDCl3
77.05 CDCl3
76.73 CDCl3
73.10
57.78
55.95
55.31
44.31
43.30
41.77
39.37
38.75
33.34
33.25
32.53
23.74
21.41
20.50
18.36
15.49
f1 (ppm)

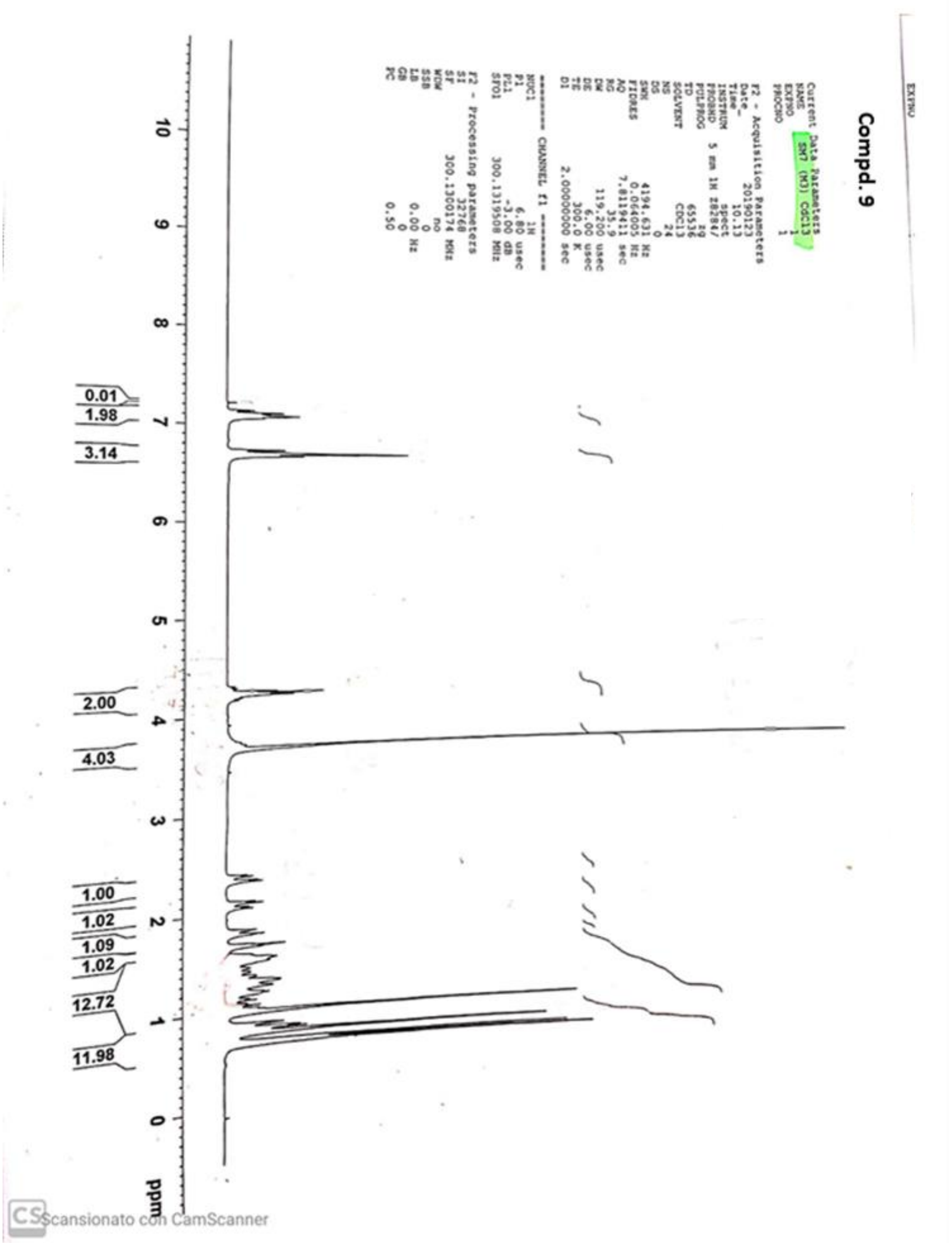
Compd. 9
ppm
Scansionato con CamScanner

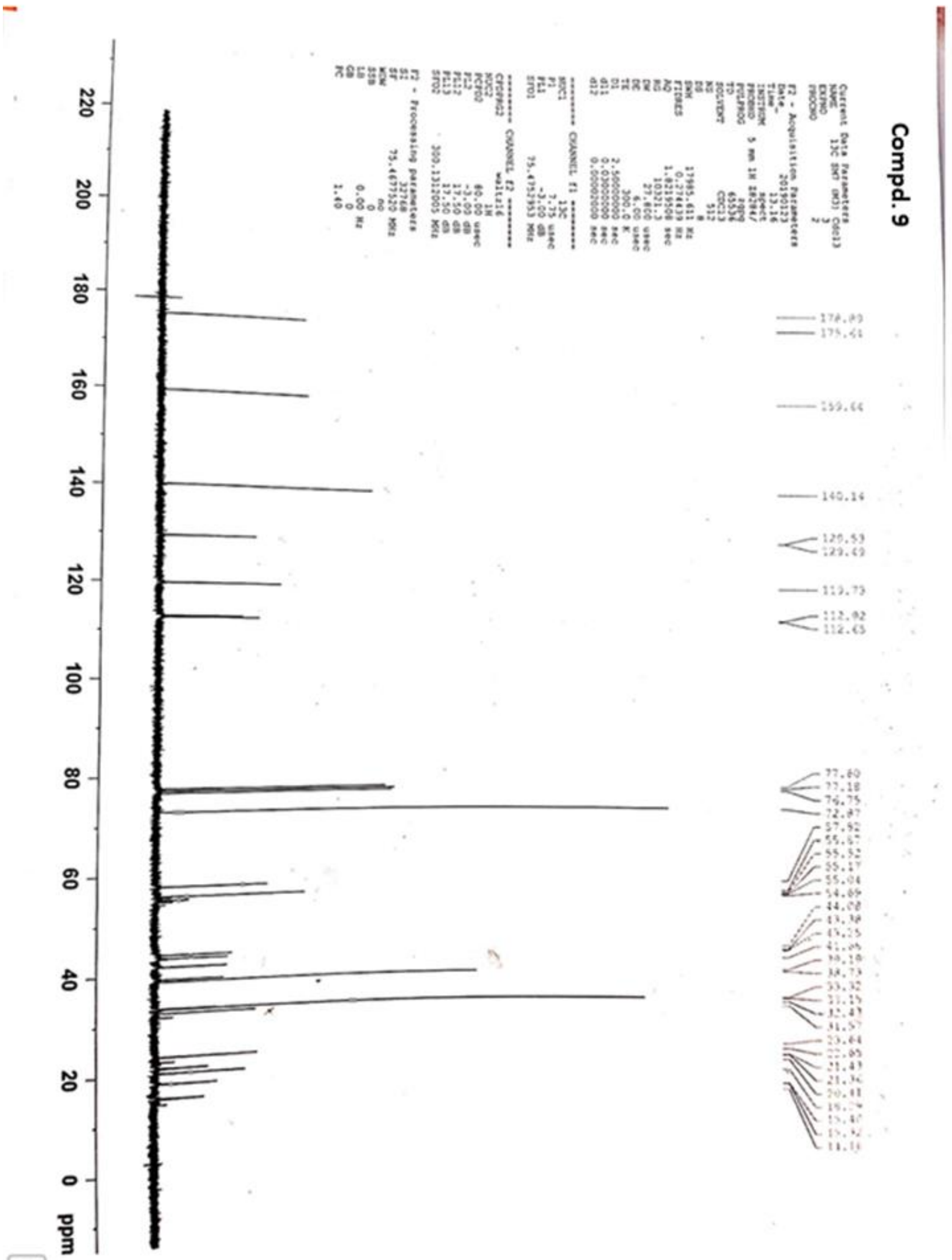

CS Scansionato con CamScanner

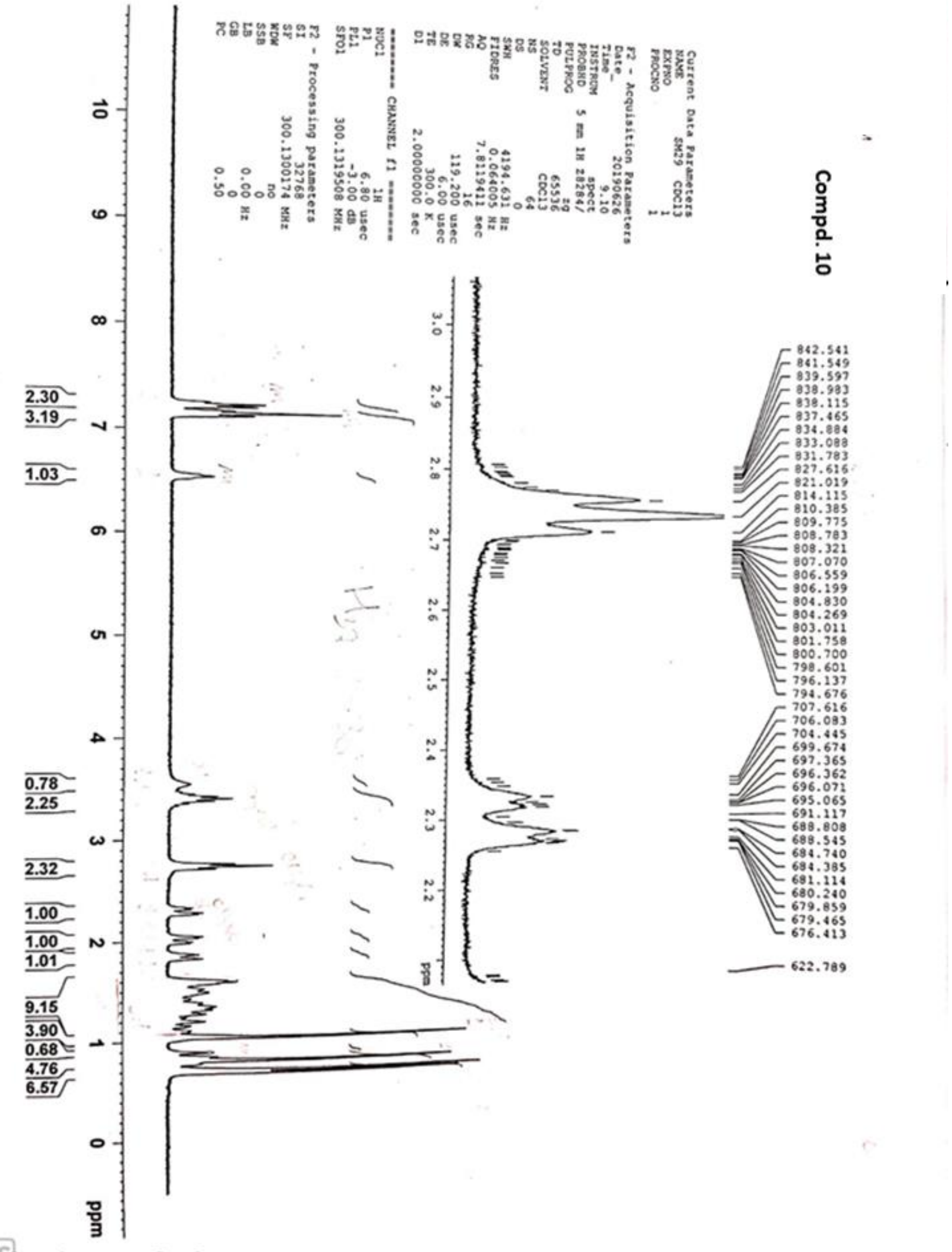

Scansionato con CamScanner

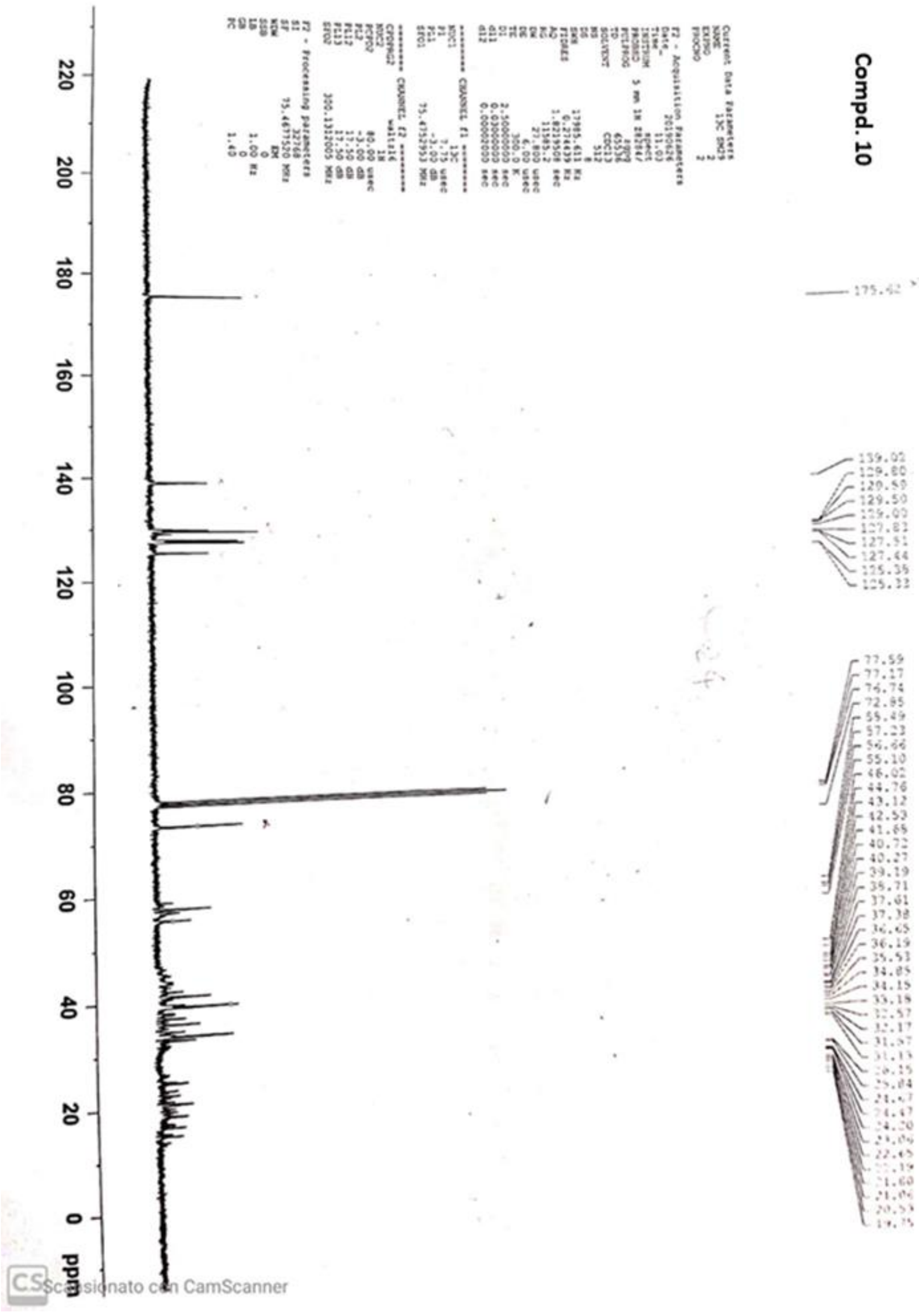

Compd. 10

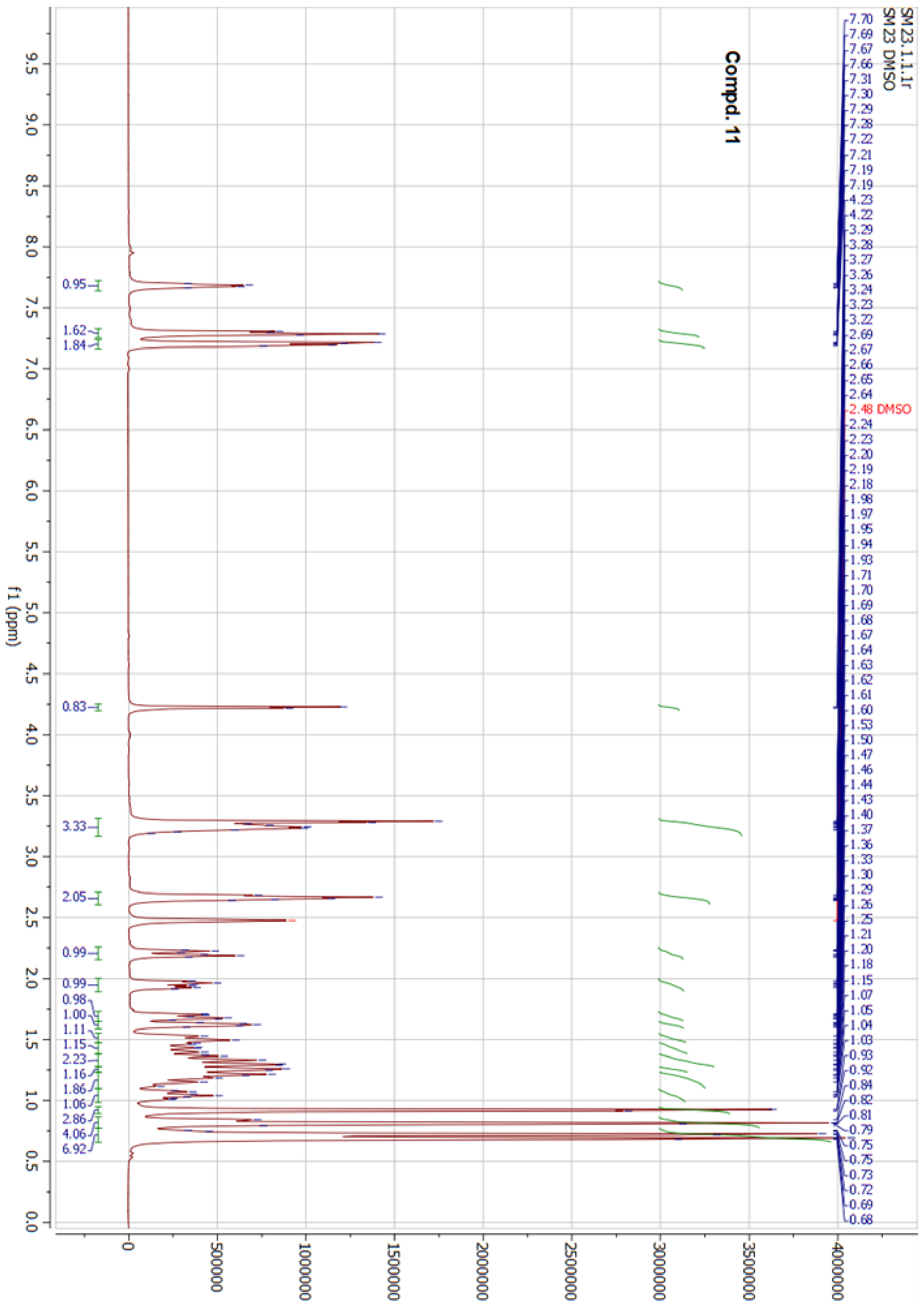
SM23.1.1.1r
SM23 DMSO
Compd. 11
7.70 7.69 7.67 7.66 7.31 7.30 7.29 7.28 7.22 7.21 7.19 7.19 4.23 4.22 3.29 3.28 3.27 3.26 3.24 3.23 3.22 2.69 2.67 2.66 2.65 2.64
2.48 DMSO
2.24 2.23 2.20 2.19 2.18 1.98 1.97 1.95 1.94 1.93 1.71 1.70 1.69 1.68 1.67 1.64 1.63 1.62 1.61 1.60 1.53 1.50 1.47 1.46 1.44 1.43 1.40 1.37 1.36 1.33 1.30 1.29 1.26 1.25 1.21 1.20 1.18 1.15 1.07 1.05 1.04 1.03 0.93 0.92 0.84 0.82 0.81 0.79 0.75 0.75 0.73 0.72 0.69 0.68
0.95 1.62 1.84 0.83 3.33 2.05 0.99 0.99 0.98 1.00 1.11 1.15 2.23 1.16 1.86 1.06 2.86 4.06 6.92
f1 (ppm)

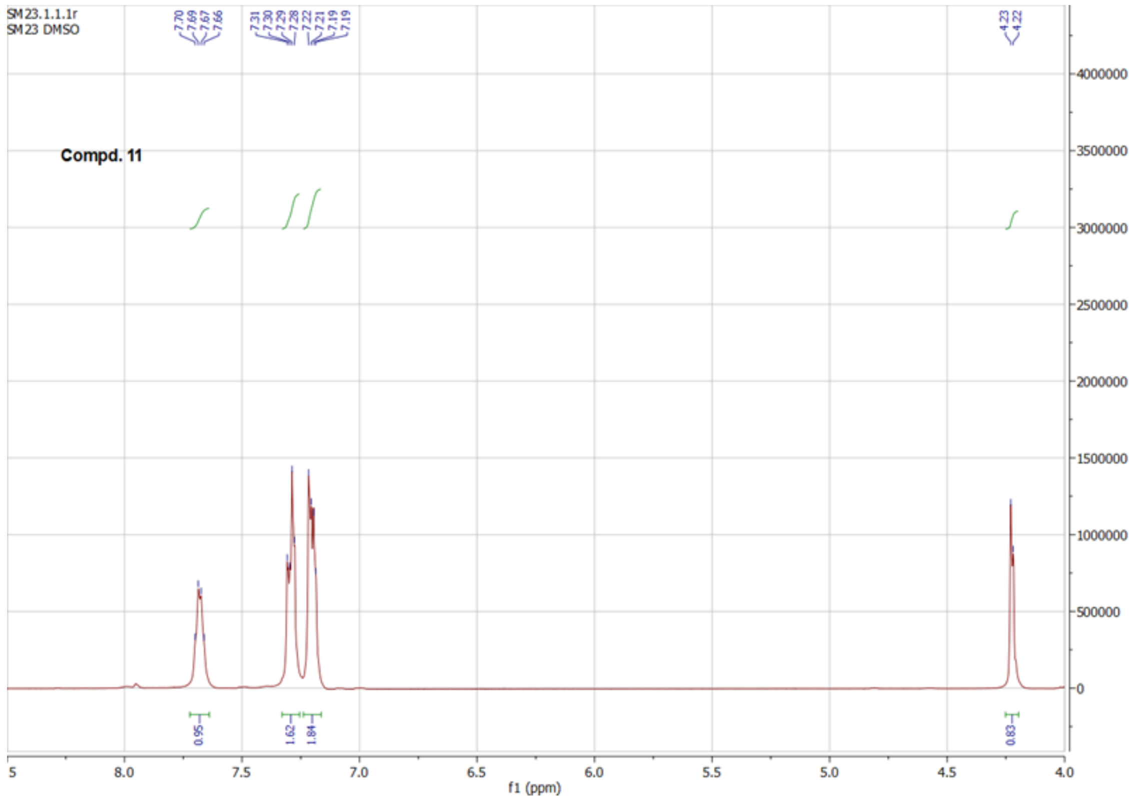
SM23.1.1.1r
SM23 DMSO
Compd. 11
f1 (ppm)

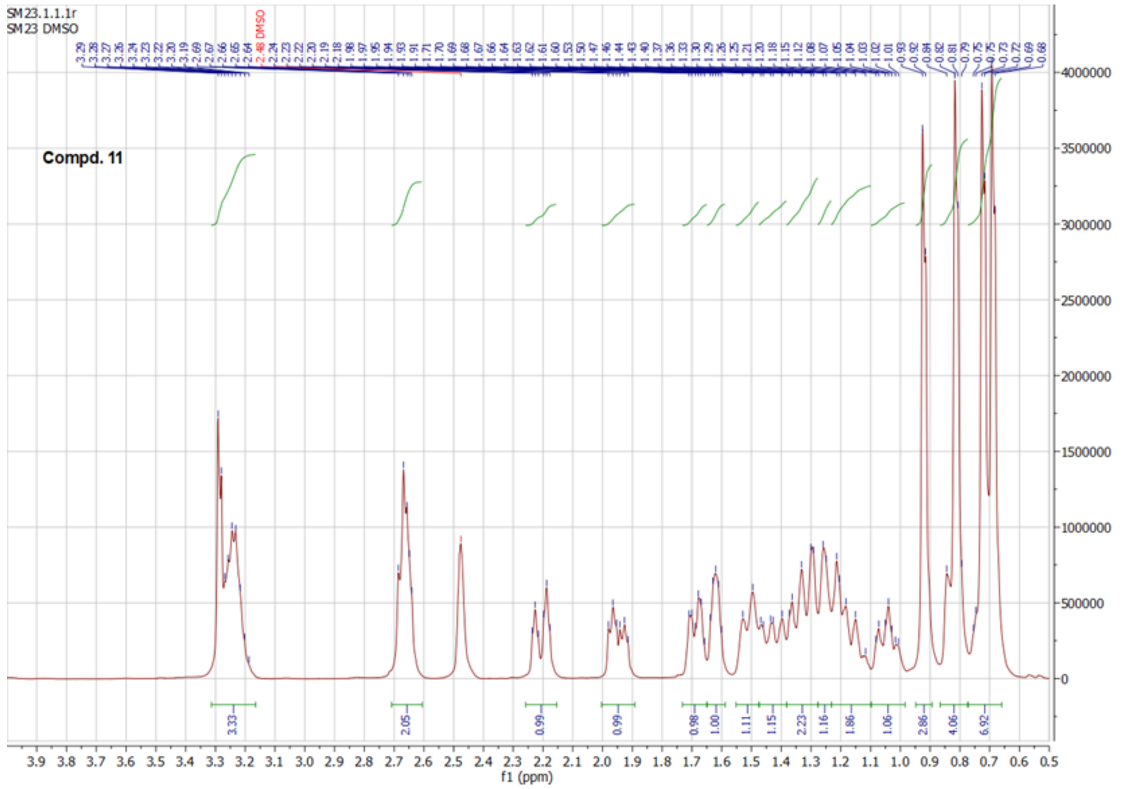
SM23.1.1.1r
SM23 DMSO
Compd. 11
2.48 DMSO
f1 (ppm)

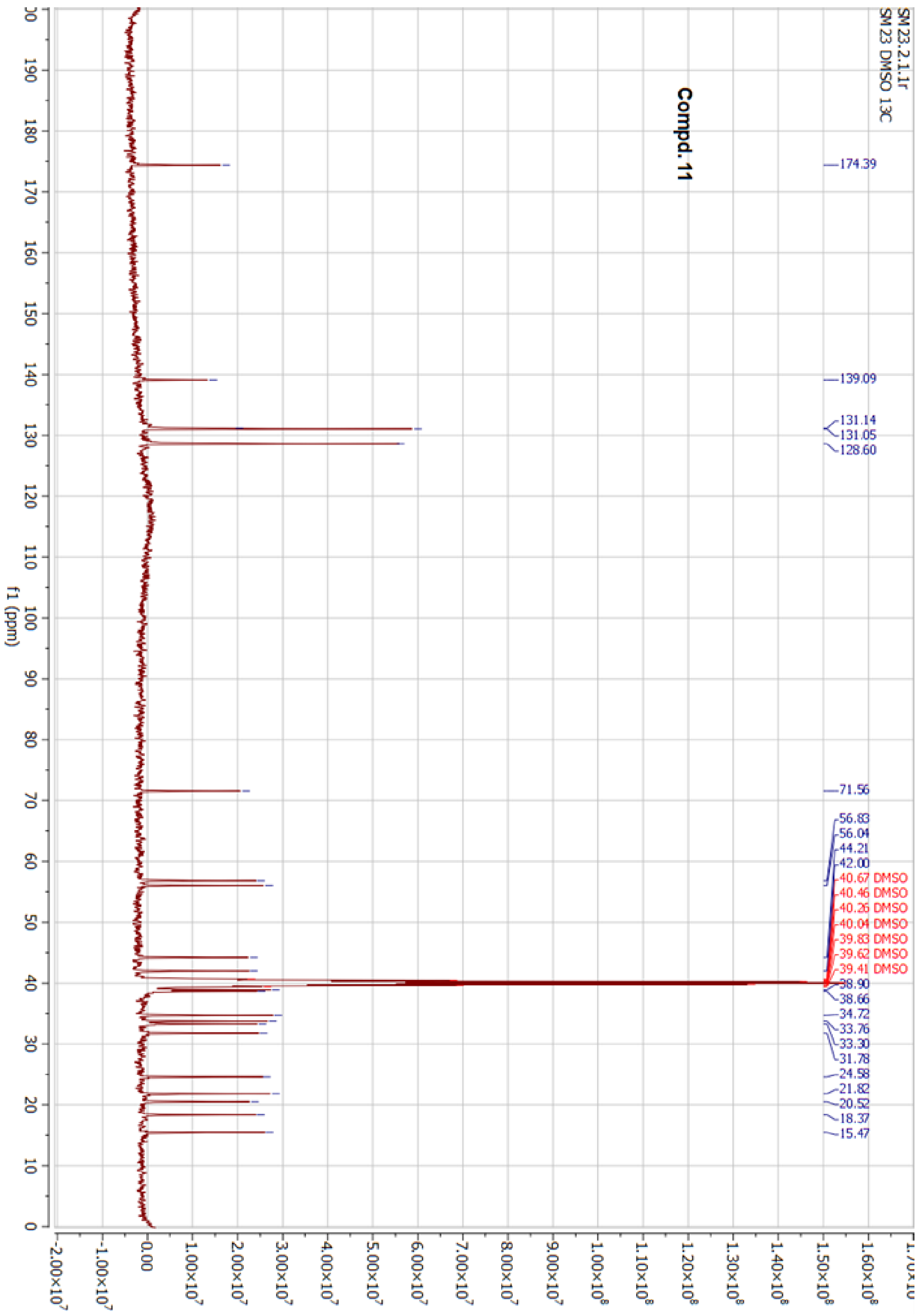
SM 23.2.1.1r
SM 23 DMSO 13C
Compd. 11
174.39
139.09
131.14
131.05
128.60
71.56
56.83
56.04
44.21
42.00
40.67 DMSO
40.46 DMSO
40.26 DMSO
40.04 DMSO
39.83 DMSO
39.62 DMSO
39.41 DMSO
38.90
38.66
34.72
33.76
33.30
31.78
24.58
21.82
20.52
18.37
15.47
f1 (ppm)

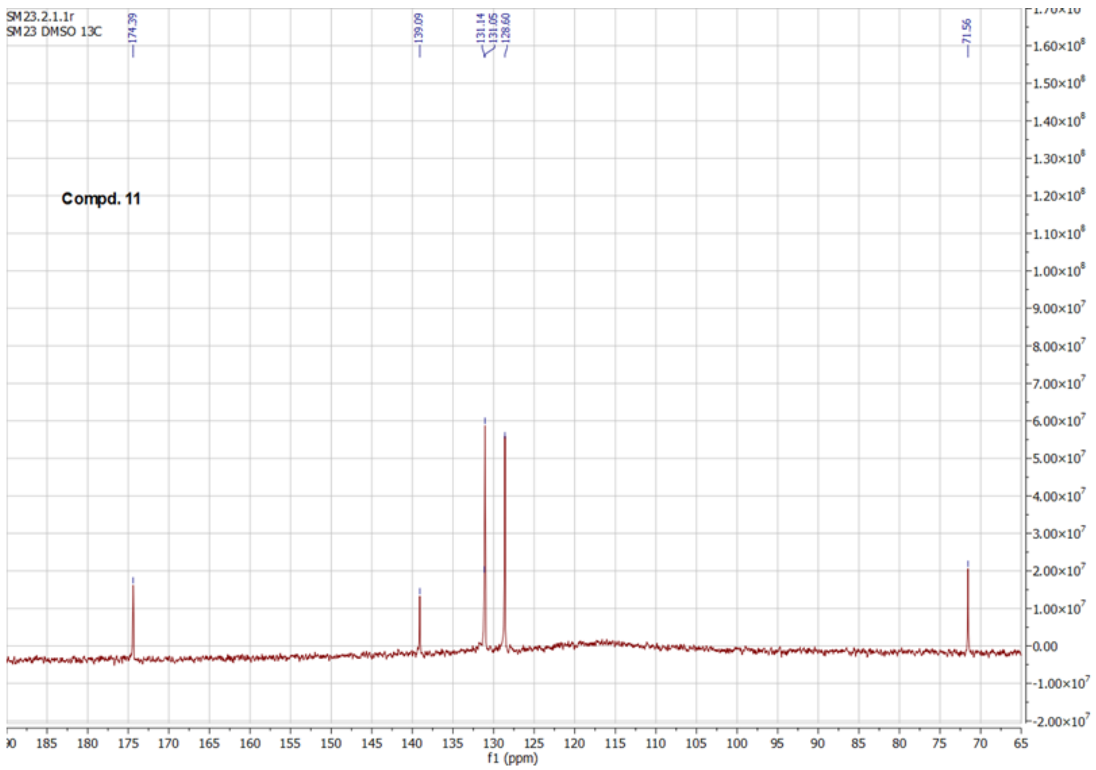
SM23.2.1.1r
SM23 DMSO 13C
Compd. 11
174.39
139.09
131.14
131.05
128.60
71.56
f1 (ppm)

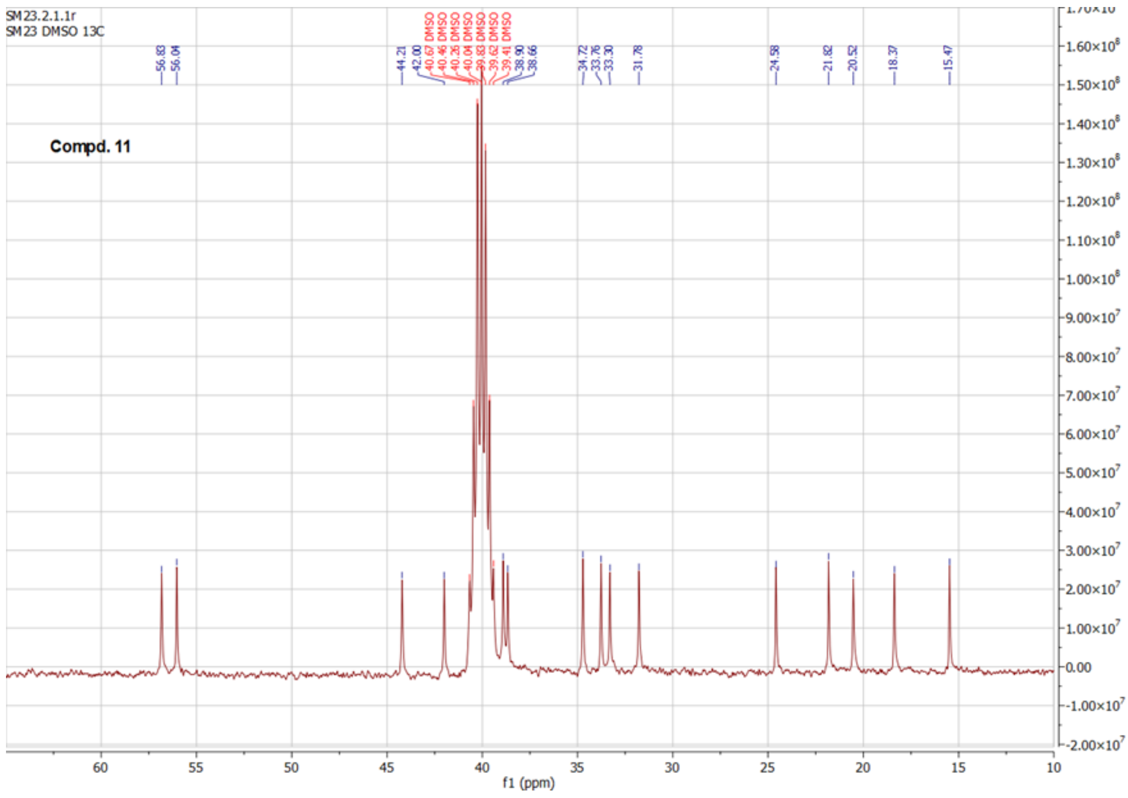
SM23.2.1.1r
SM23 DMSO 13C
Compd. 11
56.83
56.04
44.21
42.00
40.67 DMSO
40.46 DMSO
40.26 DMSO
40.04 DMSO
39.83 DMSO
39.62 DMSO
39.41 DMSO
38.90
38.66
34.72
33.76
33.30
31.78
24.58
21.82
20.52
18.37
15.47
f1 (ppm)

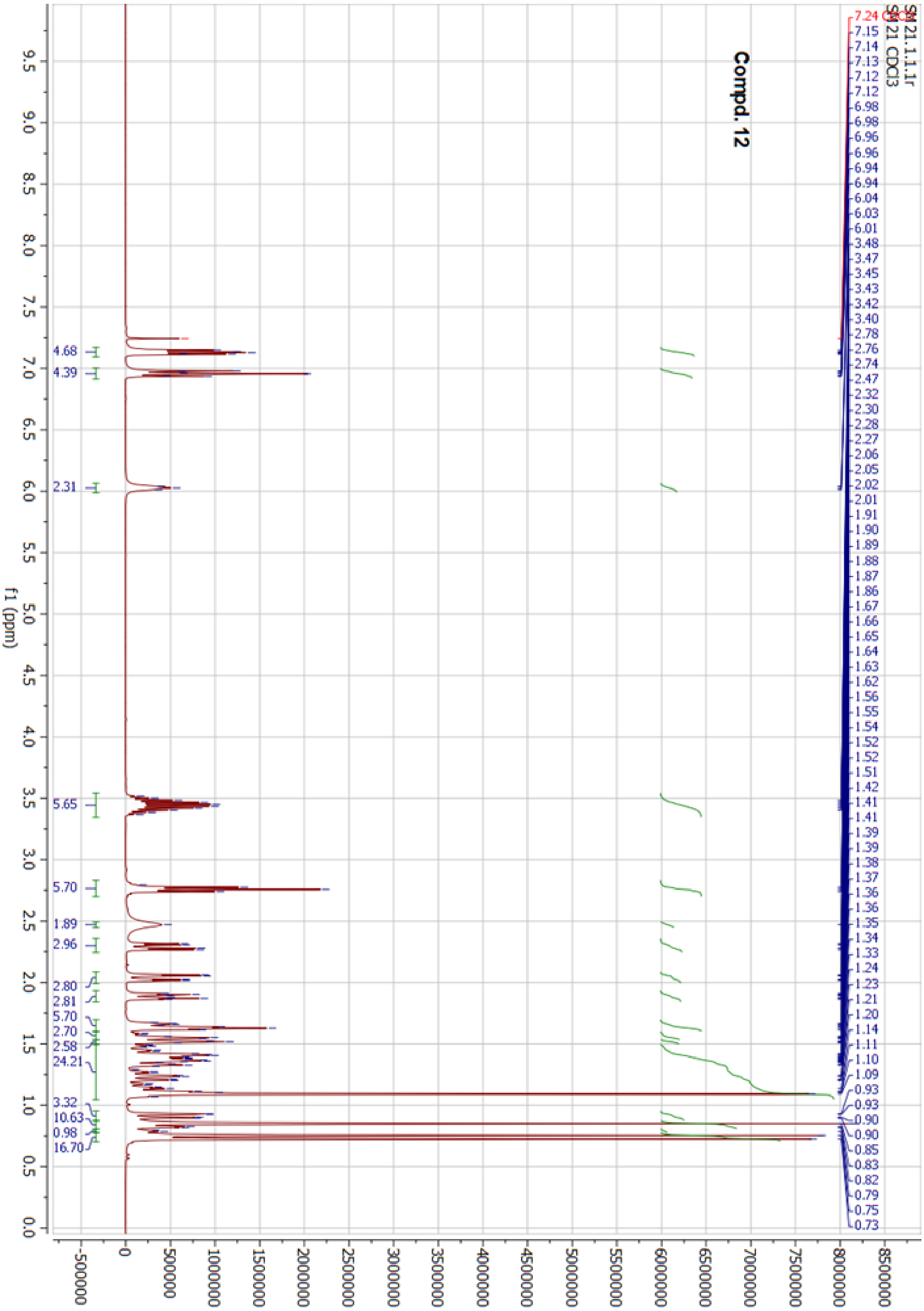
Compd. 12
f1 (ppm)

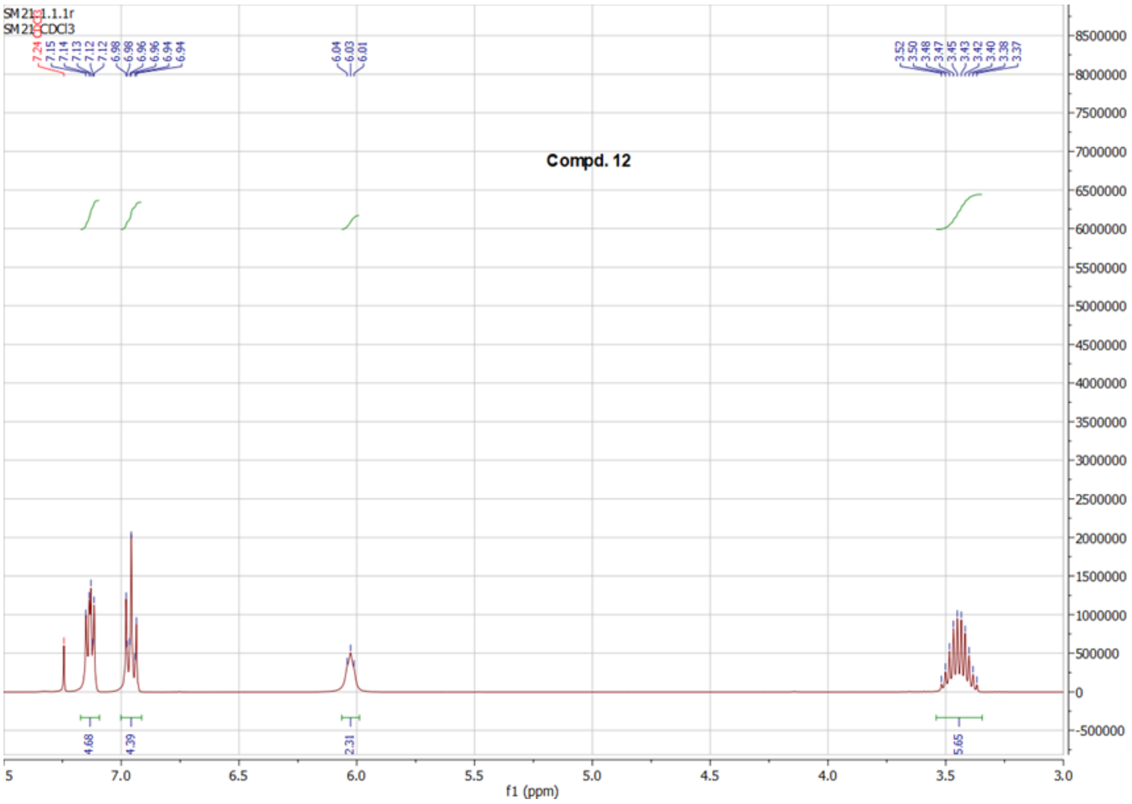
SM21-1.1.1r
SM21-CDCl3
Compd. 12
f1 (ppm)

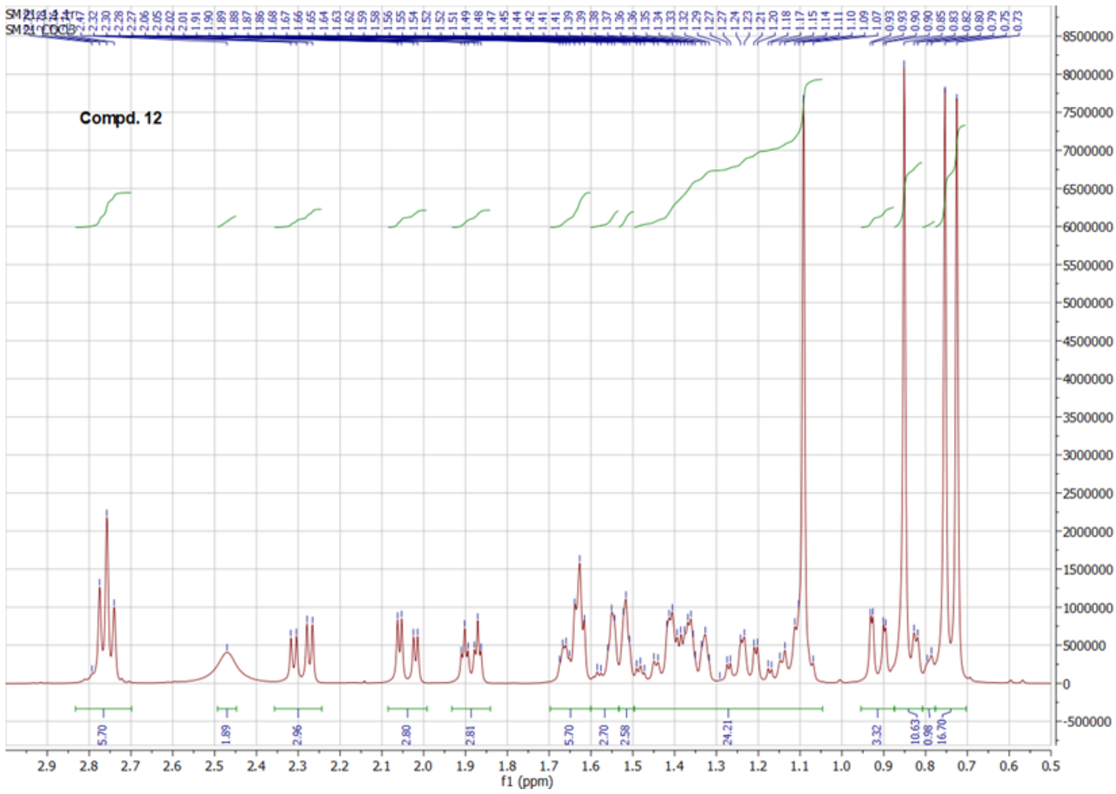
Compd. 12
f1 (ppm)

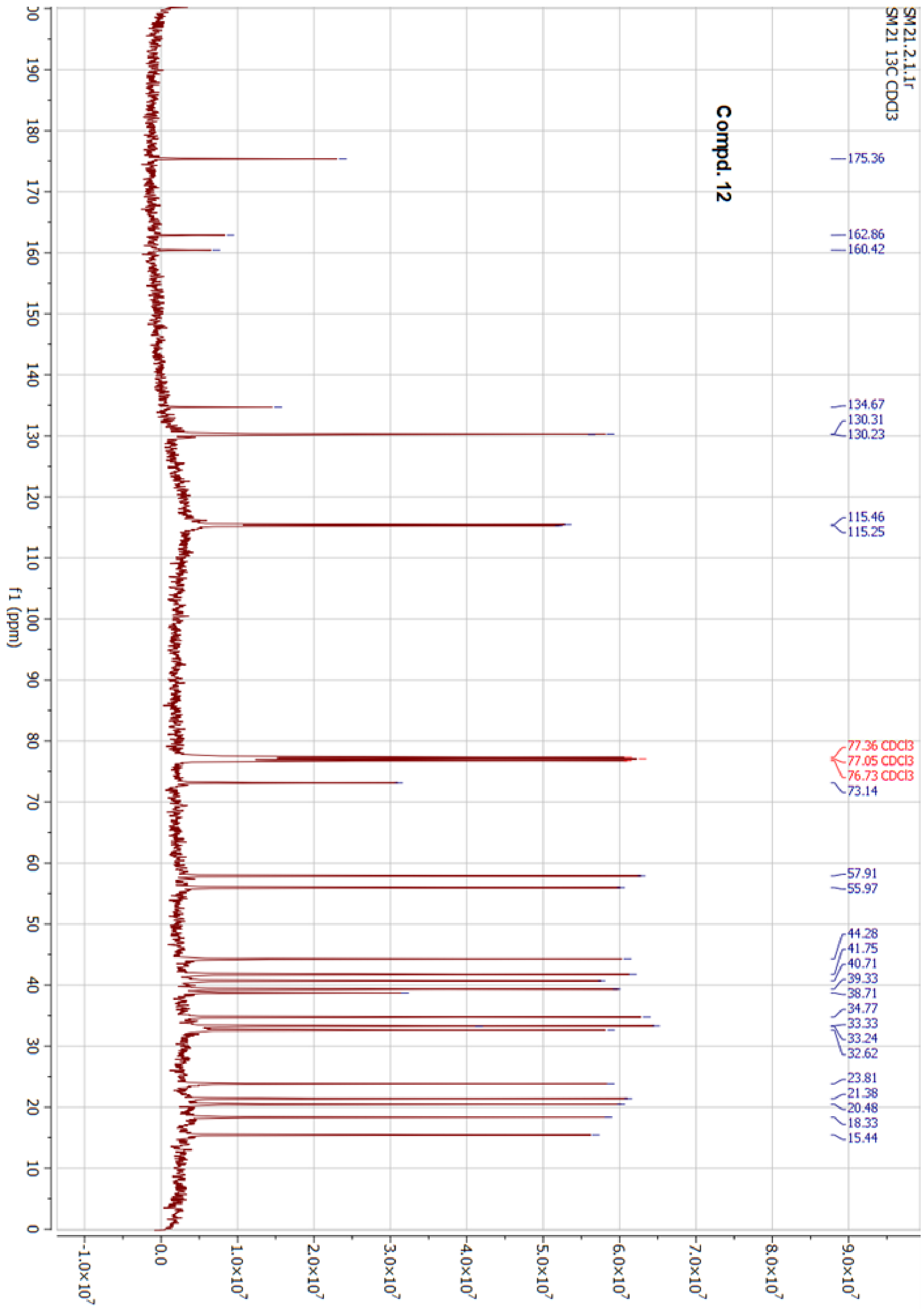
SM21.2.1.1r
SM21 13C CDCl3
Compd. 12
175.36
162.86
160.42
134.67
130.31
130.23
115.46
115.25
77.36 CDCl3
77.05 CDCl3
76.73 CDCl3
73.14
57.91
55.97
44.28
41.75
40.71
39.33
38.71
34.77
33.33
33.24
32.62
23.81
21.38
20.48
18.33
15.44
f1 (ppm)

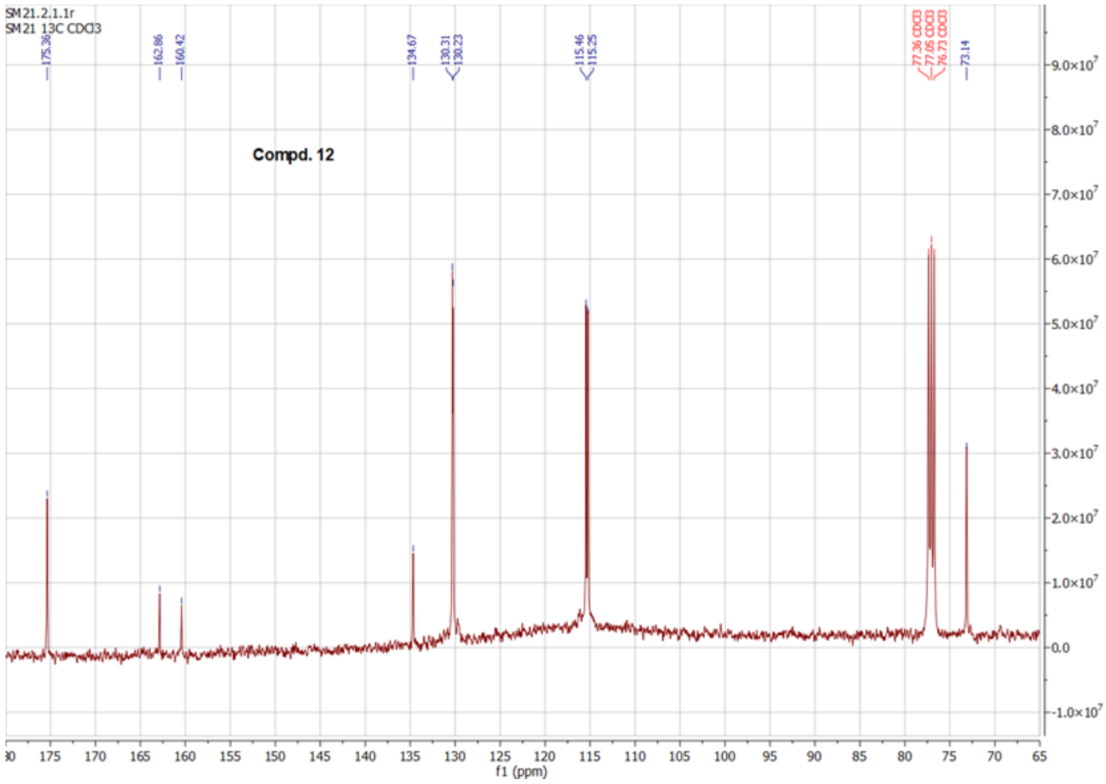
SM21.2.1.1r
SM21 13C CDCl3
175.36
162.86
160.42
134.67
130.31
130.23
115.46
115.25
77.36 CDCl3
77.05 CDCl3
76.73 CDCl3
73.14
Compd. 12
f1 (ppm)

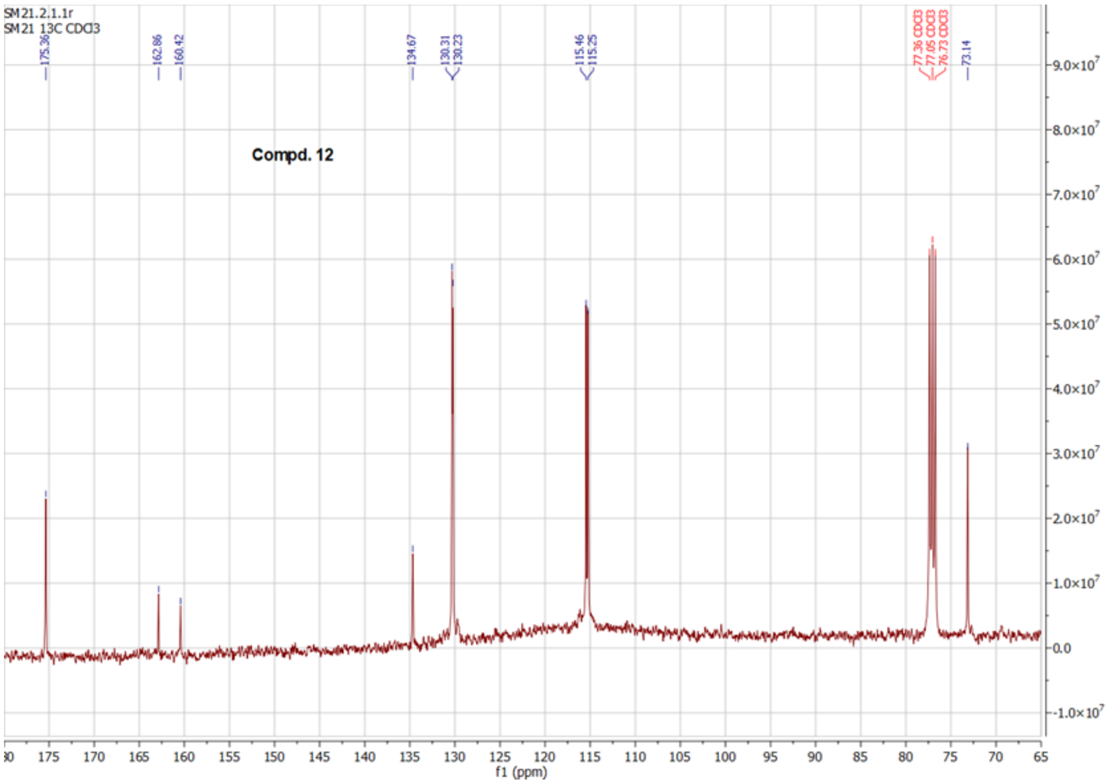
SM21.2.1.1r
SM21 13C CDCl3
175.36
162.86
160.42
134.67
130.31
130.23
115.46
115.25
77.36 CDCl3
77.05 CDCl3
76.73 CDCl3
73.14
Compd. 12
f1 (ppm)

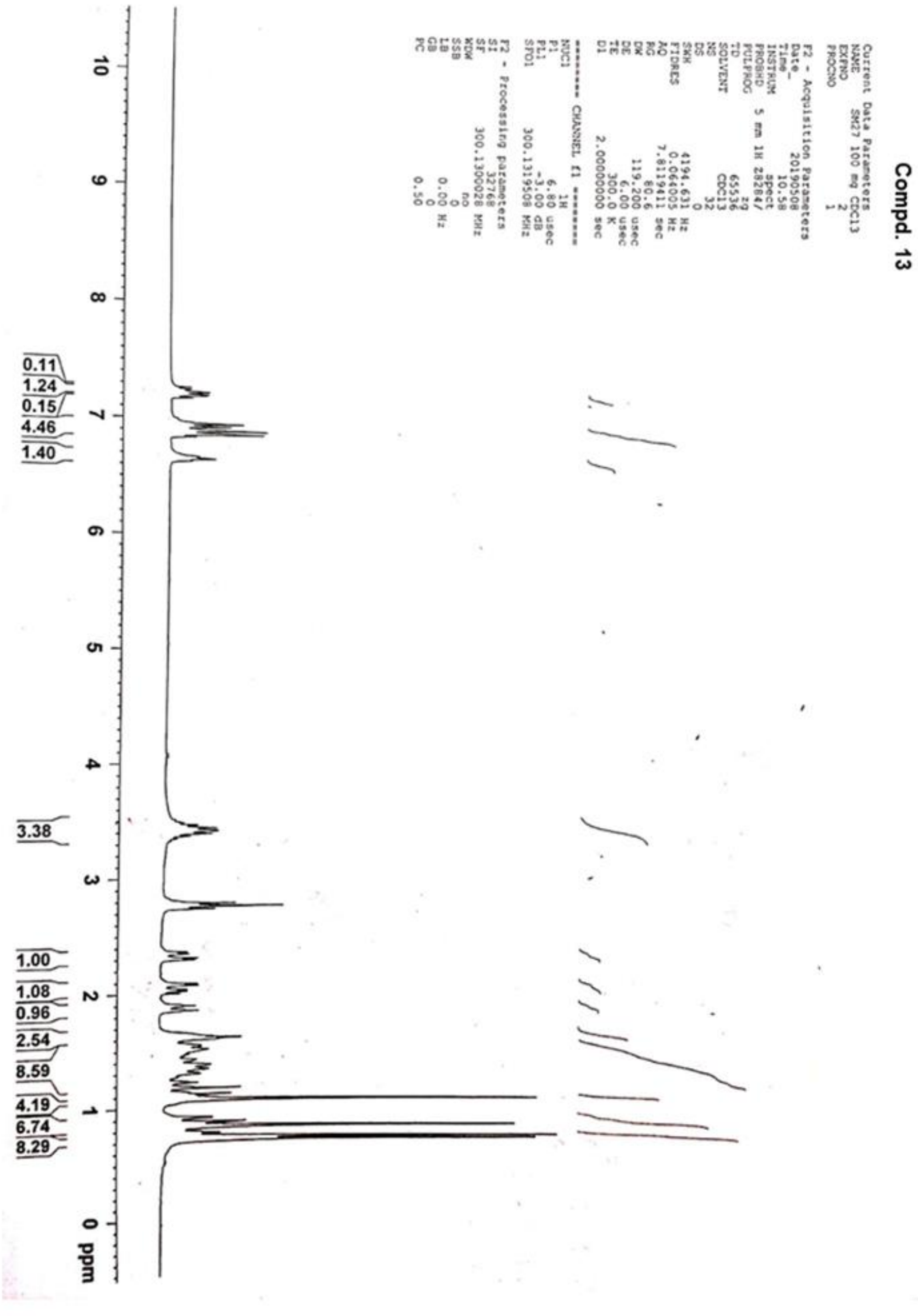

cansionato con CamScanner

Compd. 13

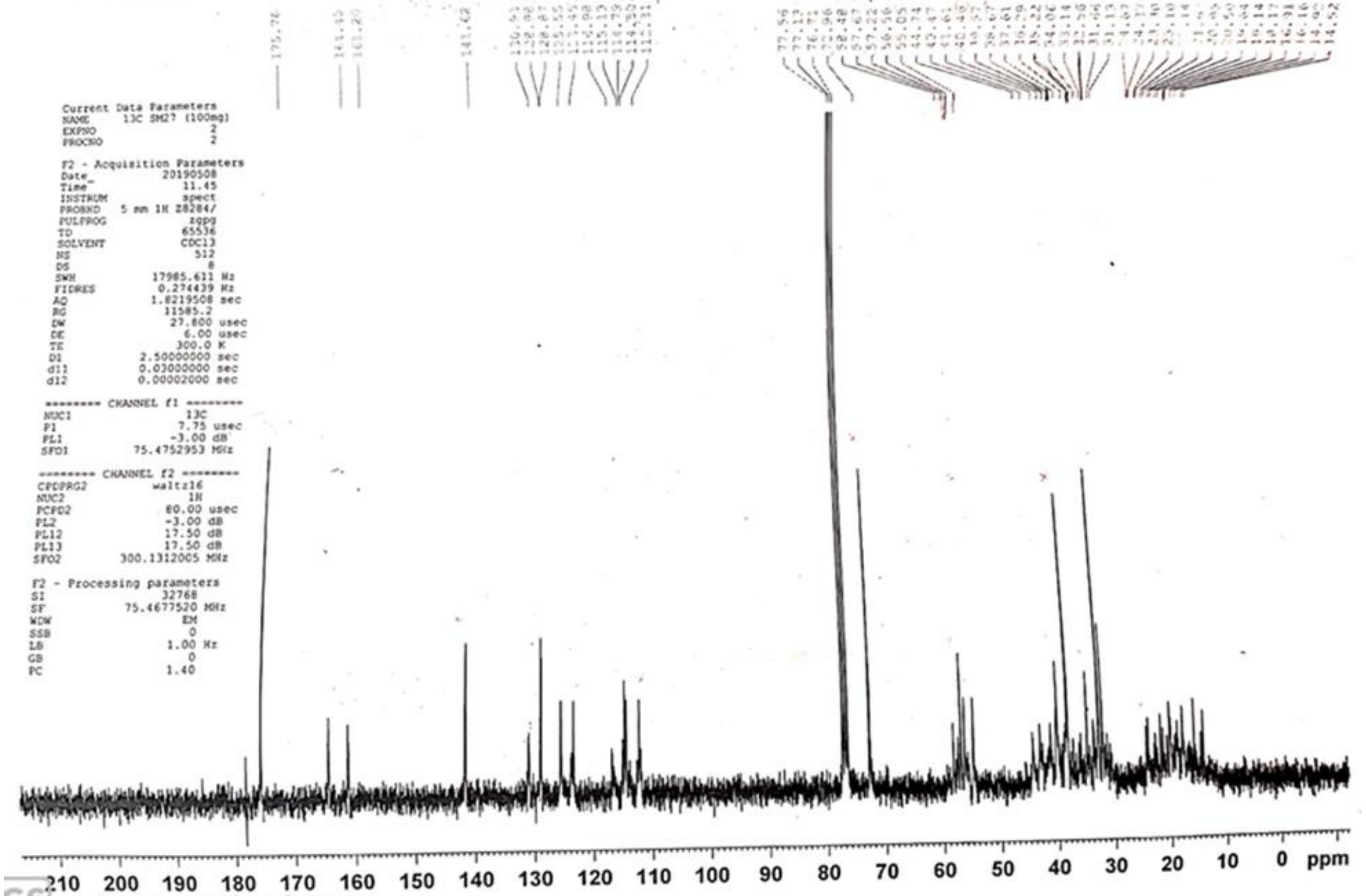

Scansionato con CamScanner

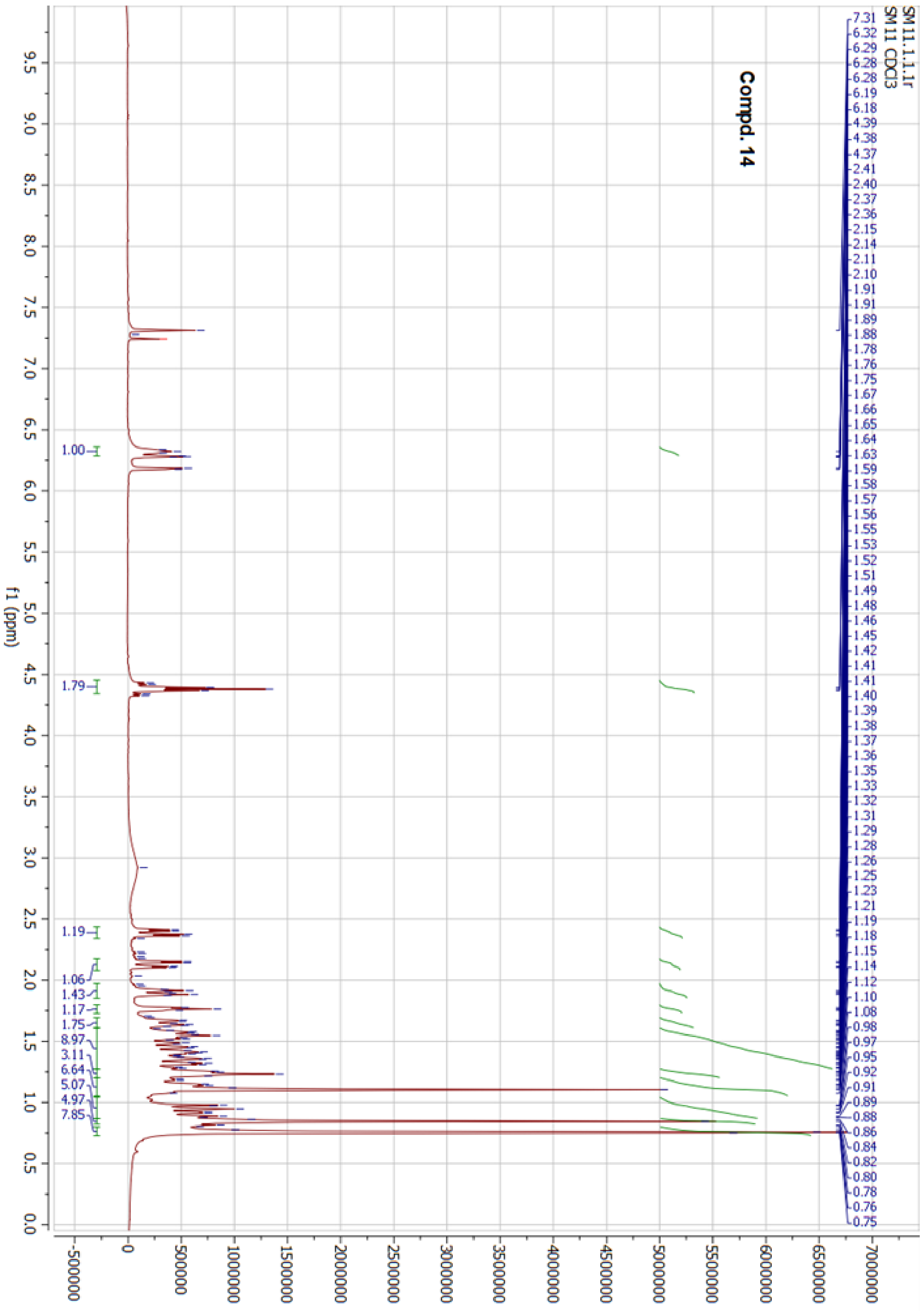
SM11.1.1.fr
SM 11 CDCl3
Compd. 14
f1 (ppm)

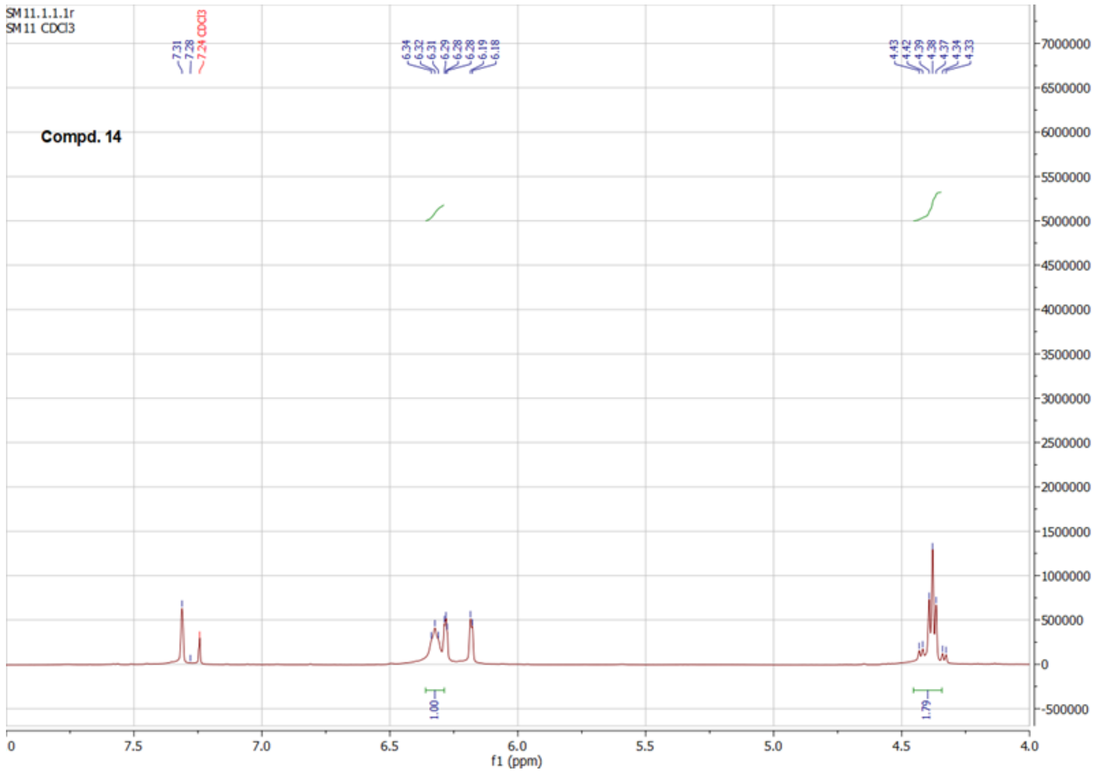
SM11.1.1.1r
SM11 CDCl3
Compd. 14
f1 (ppm)

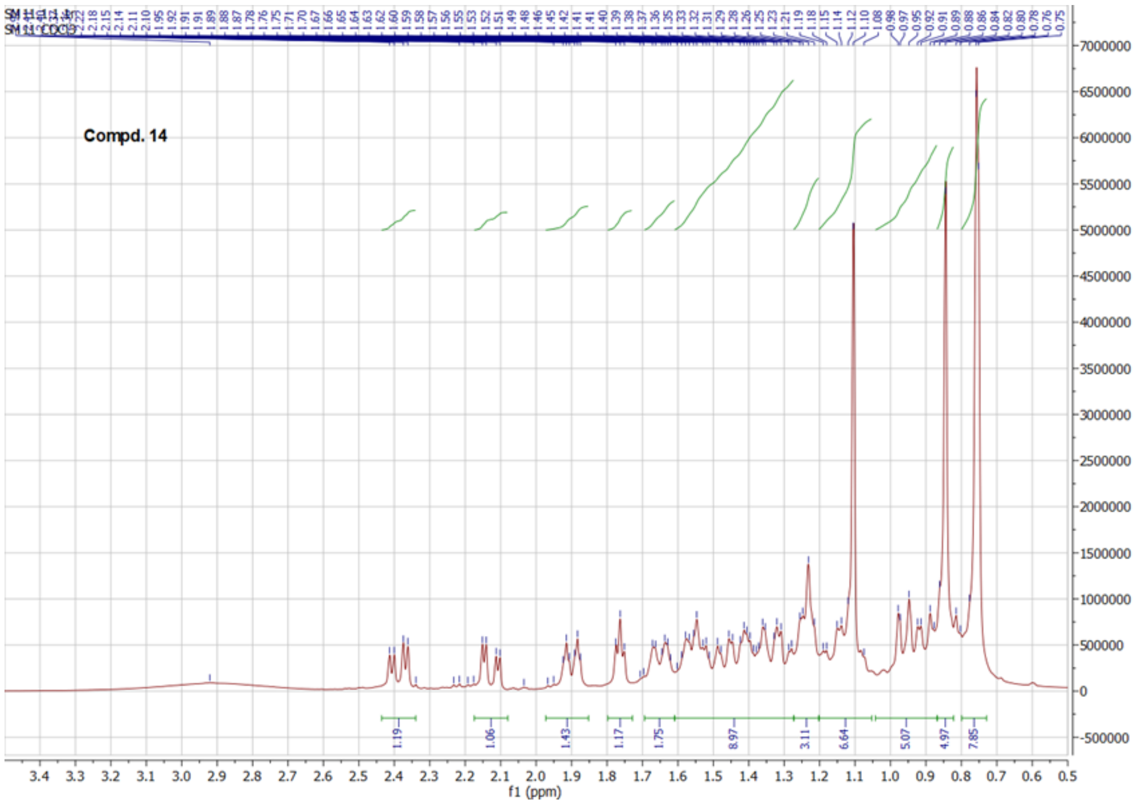
Compd. 14
f1 (ppm)

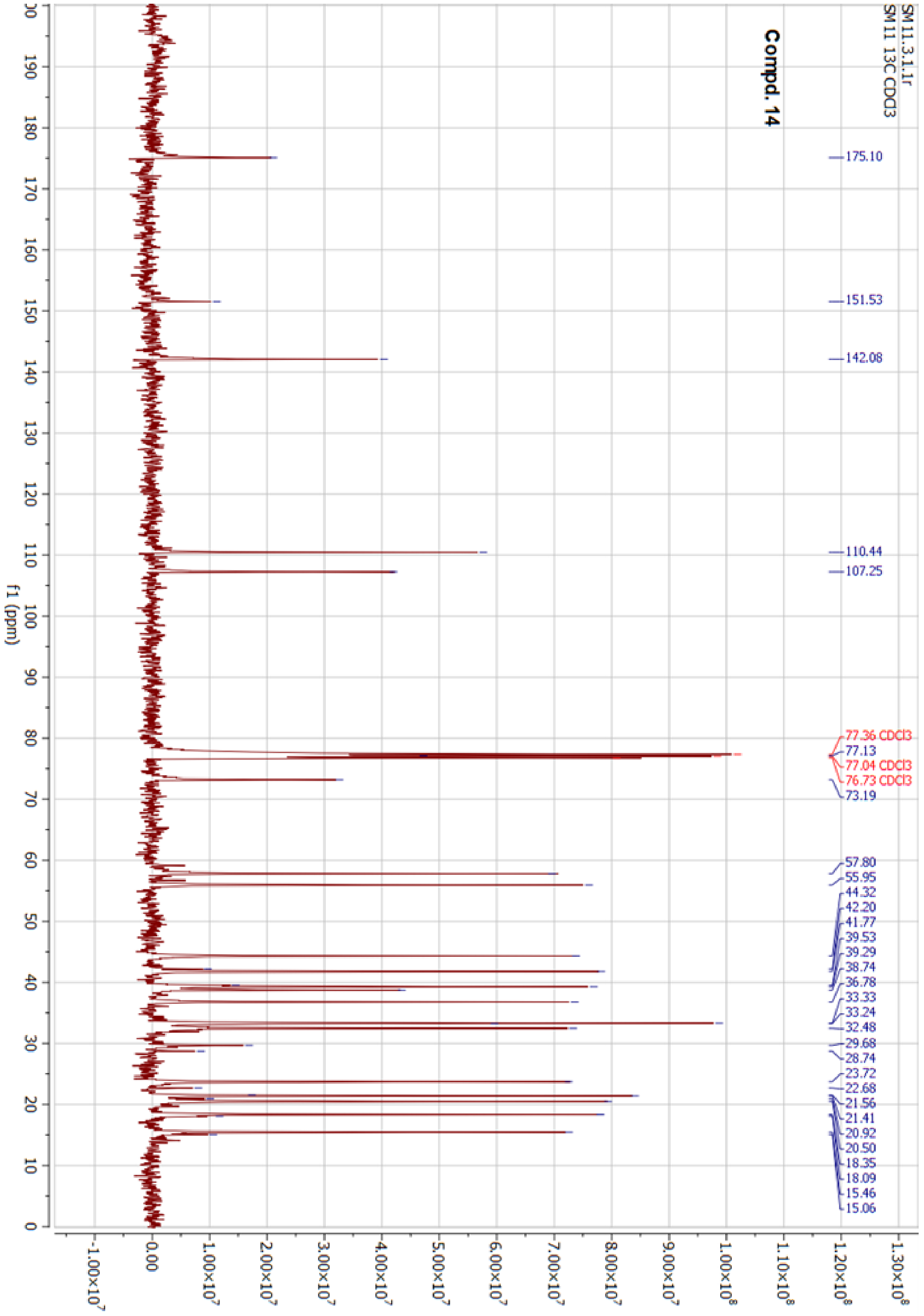
SM11.3.1.1r
SM11 13C CDCl3
Compd. 14
175.10
151.53
142.08
110.44
107.25
77.36 CDCl3
77.13
77.04 CDCl3
76.73 CDCl3
73.19
57.80
55.95
44.32
42.20
41.77
39.53
39.29
38.74
36.78
33.33
33.24
32.48
29.68
28.74
23.72
22.68
21.56
21.41
20.92
20.50
18.35
18.09
15.46
15.06
f1 (ppm)

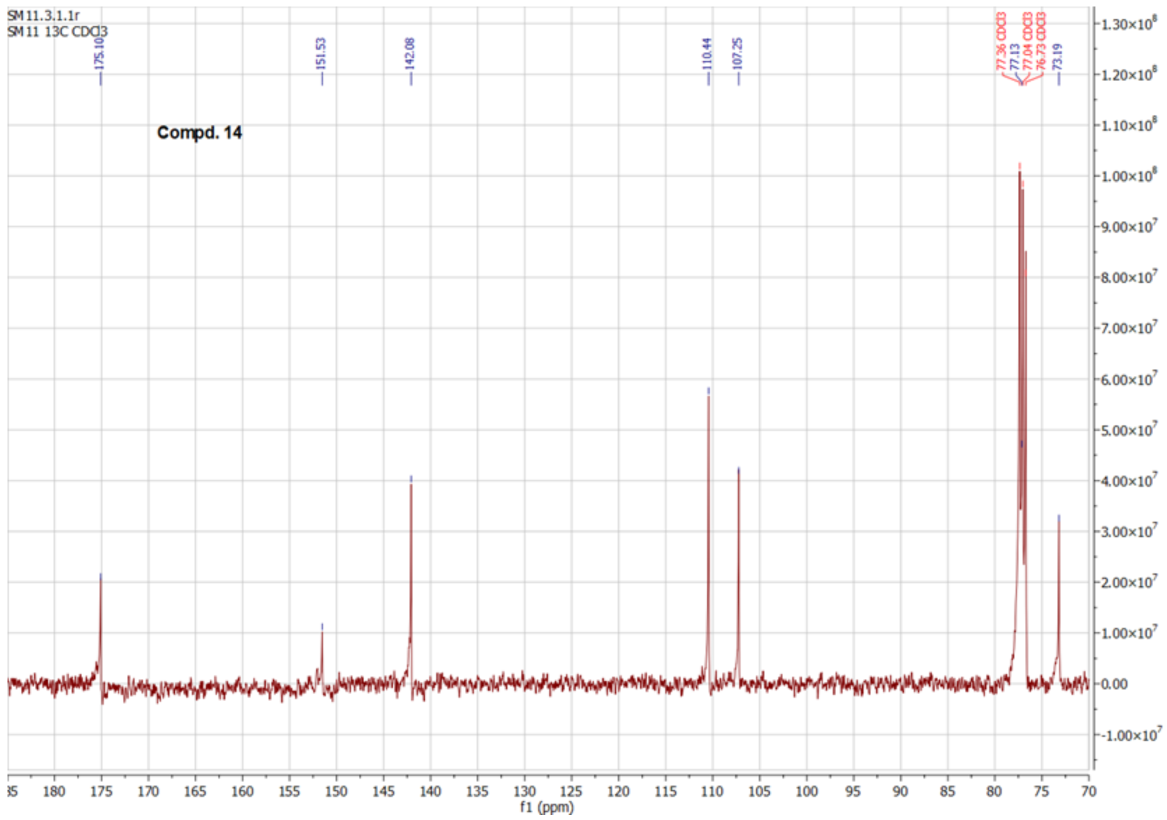
SM11.3.1.1r
SM11 13C CDCl3
Compd. 14
175.10
151.53
142.08
110.44
107.25
77.36 CDCl3
77.13
77.04 CDCl3
76.73 CDCl3
73.19
f1 (ppm)

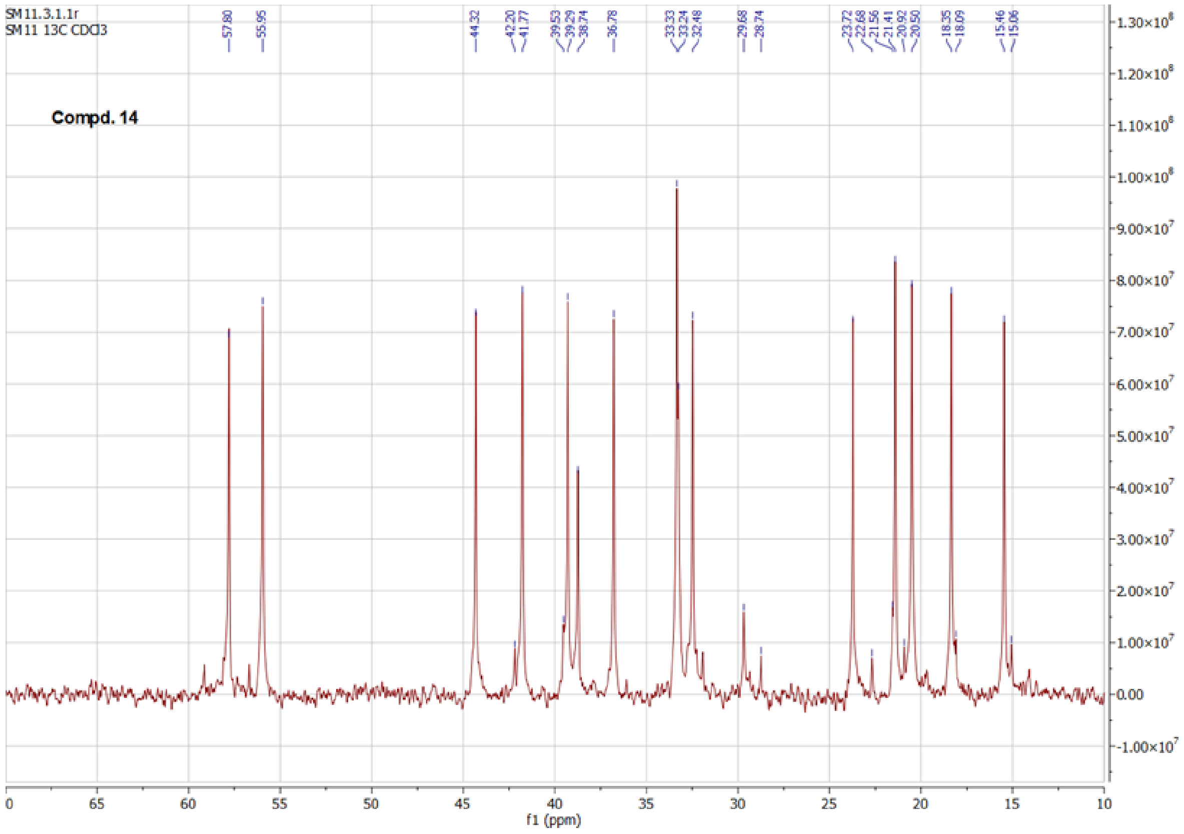
SM11.3.1.1r
SM11 13C CDCl3
Compd. 14
57.80
55.95
44.32
42.20
41.77
39.53
39.29
38.74
36.78
33.33
33.24
32.48
29.68
28.74
23.72
22.68
21.56
21.41
20.92
20.50
18.35
18.09
15.46
15.06
f1 (ppm)

Compd. 15

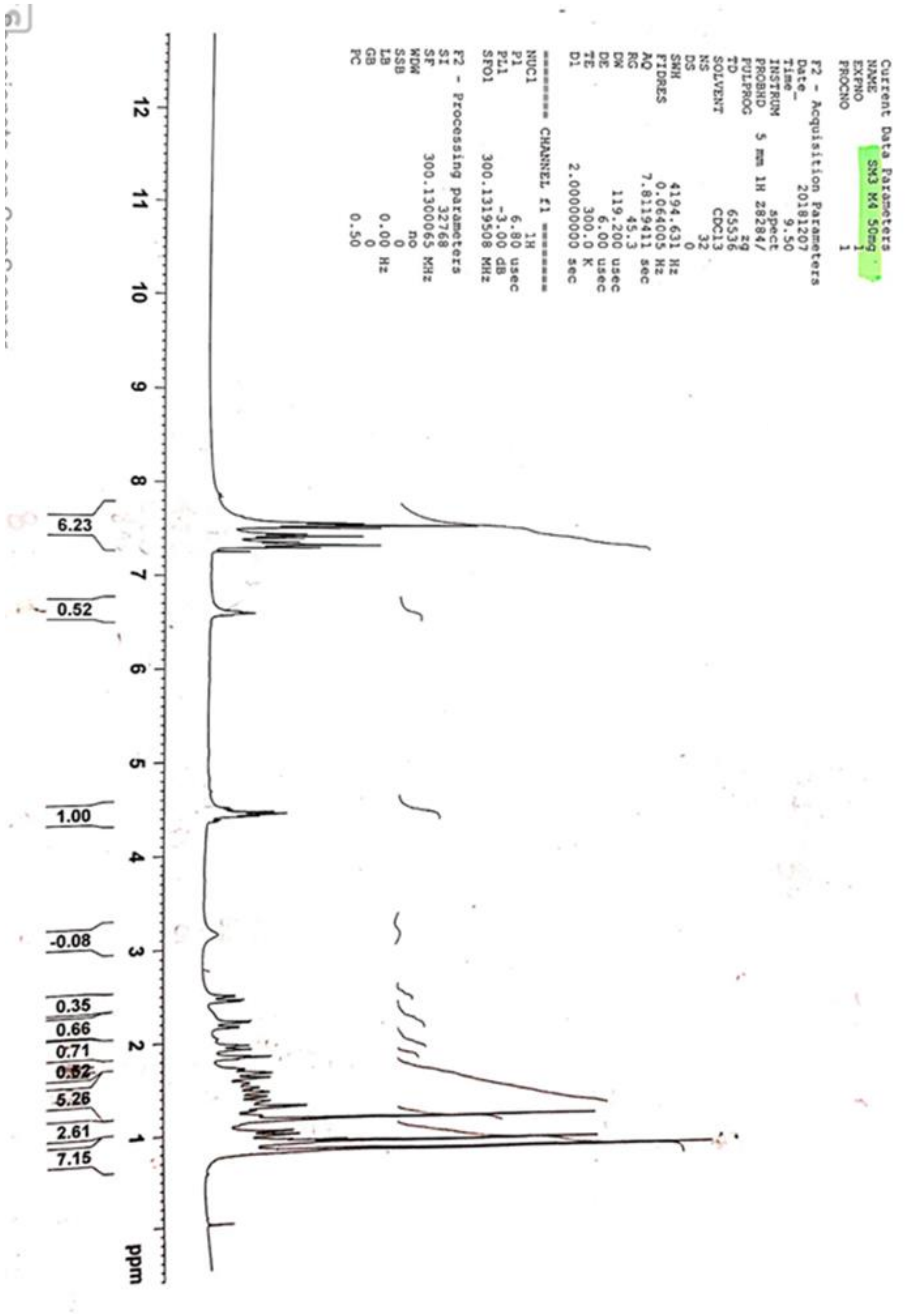
Current Data Parameters
NAME SM3 M4 50mg
EXPNO 1
PROCNO 1
F2 - Acquisition Parameters
Date_ 20181207
Time 9.50
INSTRUM spect
PROBHD 5 mm 1H Z8284/
PULPROG zg
TD 65536
SOLVENT CDCl3
NS 32
DS 0
SWH 4194.631 Hz
FIDRES 0.064005 Hz
AQ 7.8119411 sec
RG 45.3
DW 119.200 usec
DE 6.00 usec
TE 300.0 K
D1 2.0000000 sec
======== CHANNEL f1 ========
NUC1 1H
P1 6.80 usec
PL1 -3.00 dB
SFO1 300.1319508 MHz
F2 - Processing parameters
SI 32768
SF 300.1300065 MHz
WDW no
SSB 0
LB 0.00 Hz
GB 0
PC 0.50
6.23
0.52
1.00
-0.08
0.35
0.66
0.71
0.62
5.26
2.61
7.15
12
11
10
9
8
7
6
5
4
3
2
1
ppm

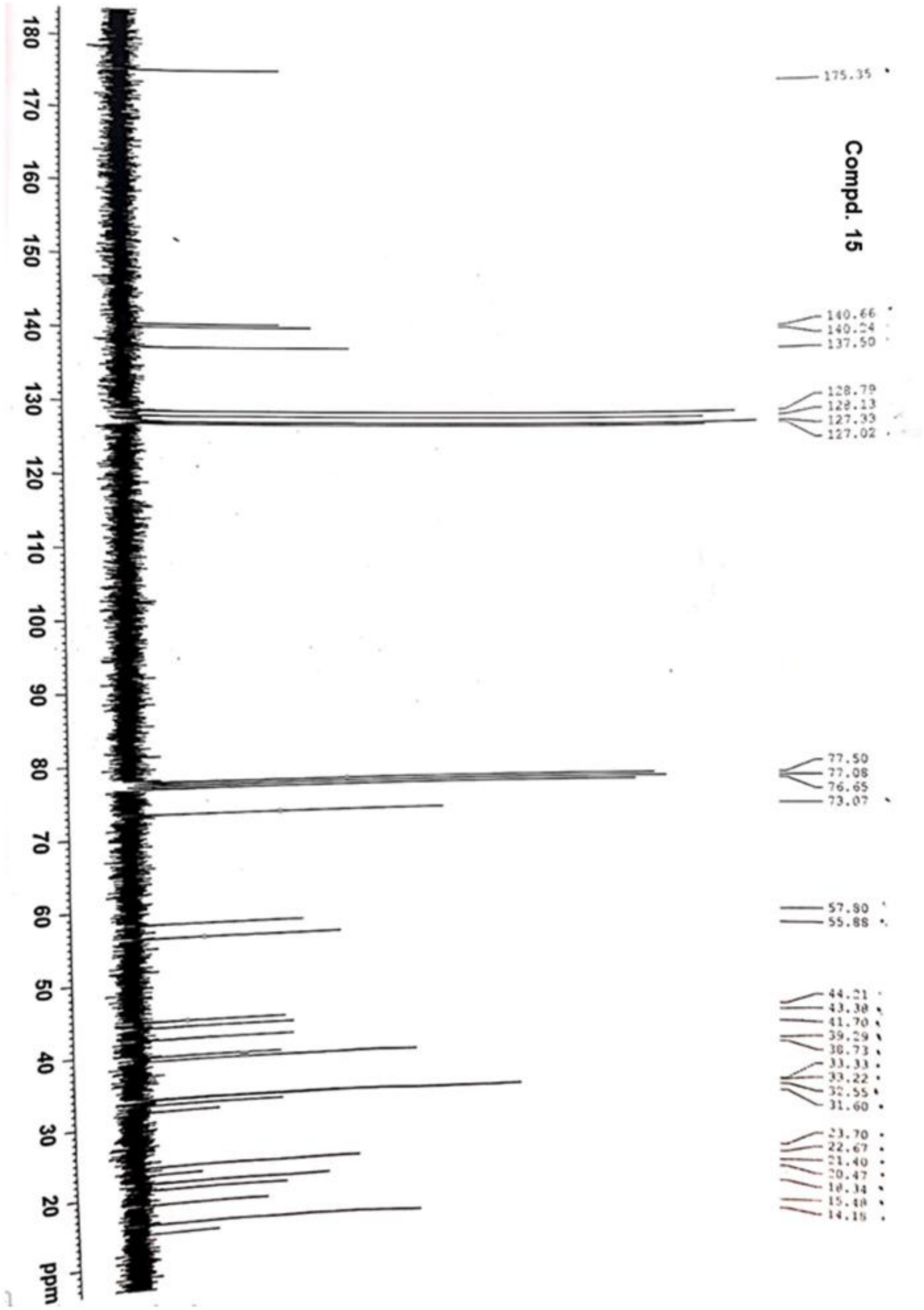
Compd. 15
175.35
140.66
140.24
137.50
128.79
128.13
127.33
127.02
77.50
77.08
76.65
73.07
57.80
55.88
44.21
43.30
41.70
39.29
38.73
33.33
33.22
32.55
31.60
23.70
22.67
21.40
20.47
18.34
15.48
14.18
ppm

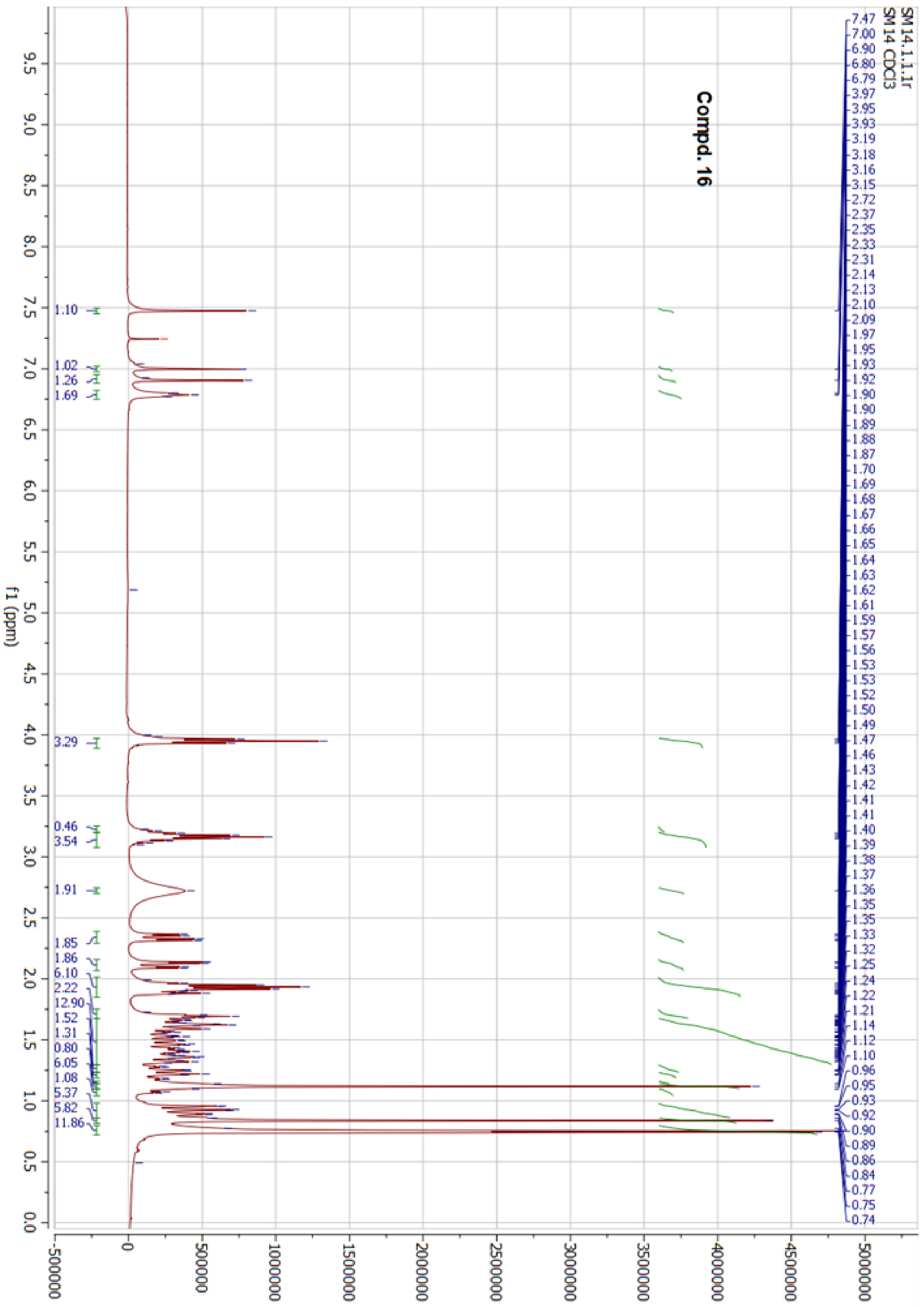
SM14.1.1.1r
SM14 CDCl3
Compd. 16
f1 (ppm)

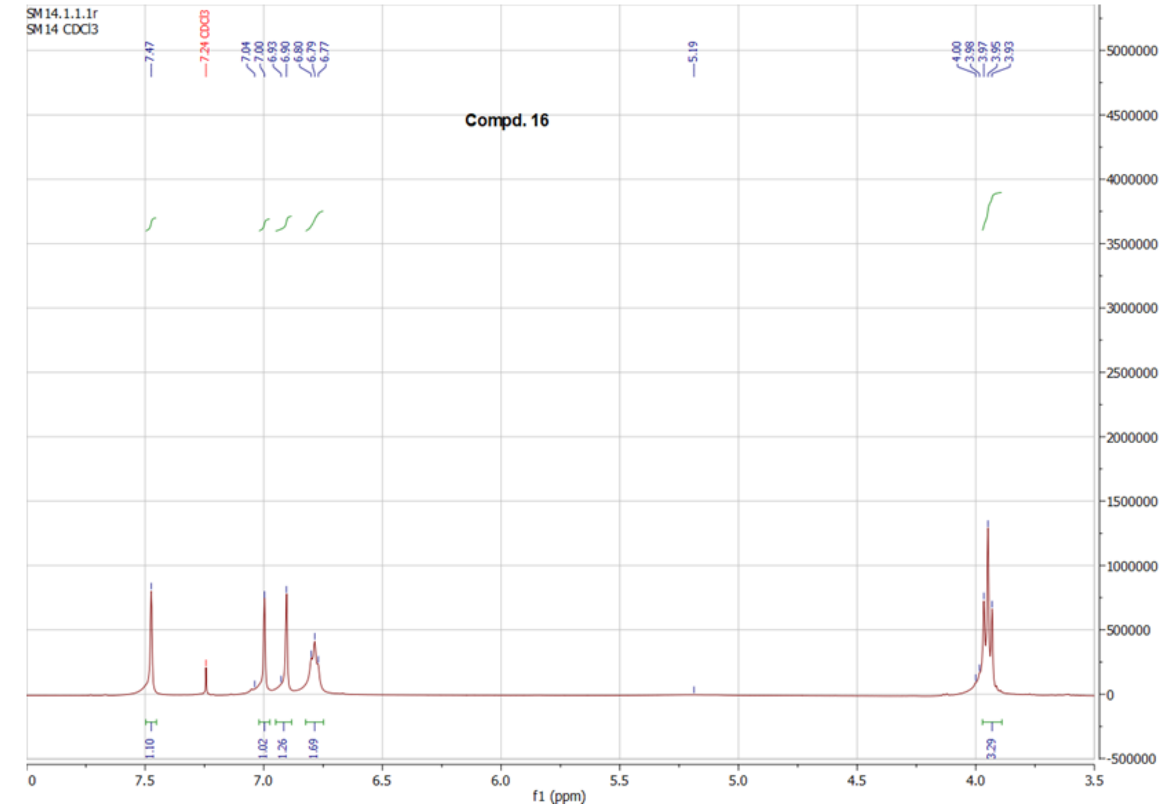
SM 14.1.1.1r
SM 14 CDCl3
Compd. 16
f1 (ppm)

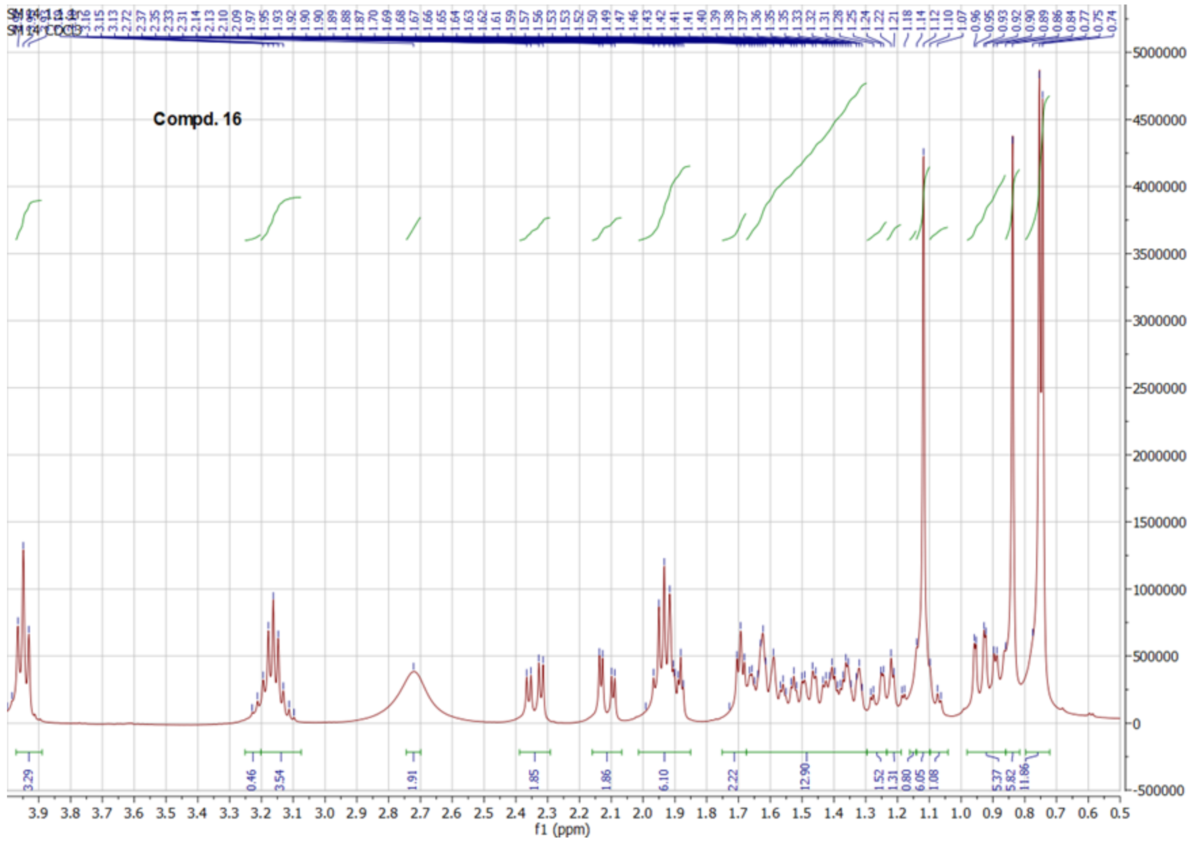
Compd. 16
f1 (ppm)

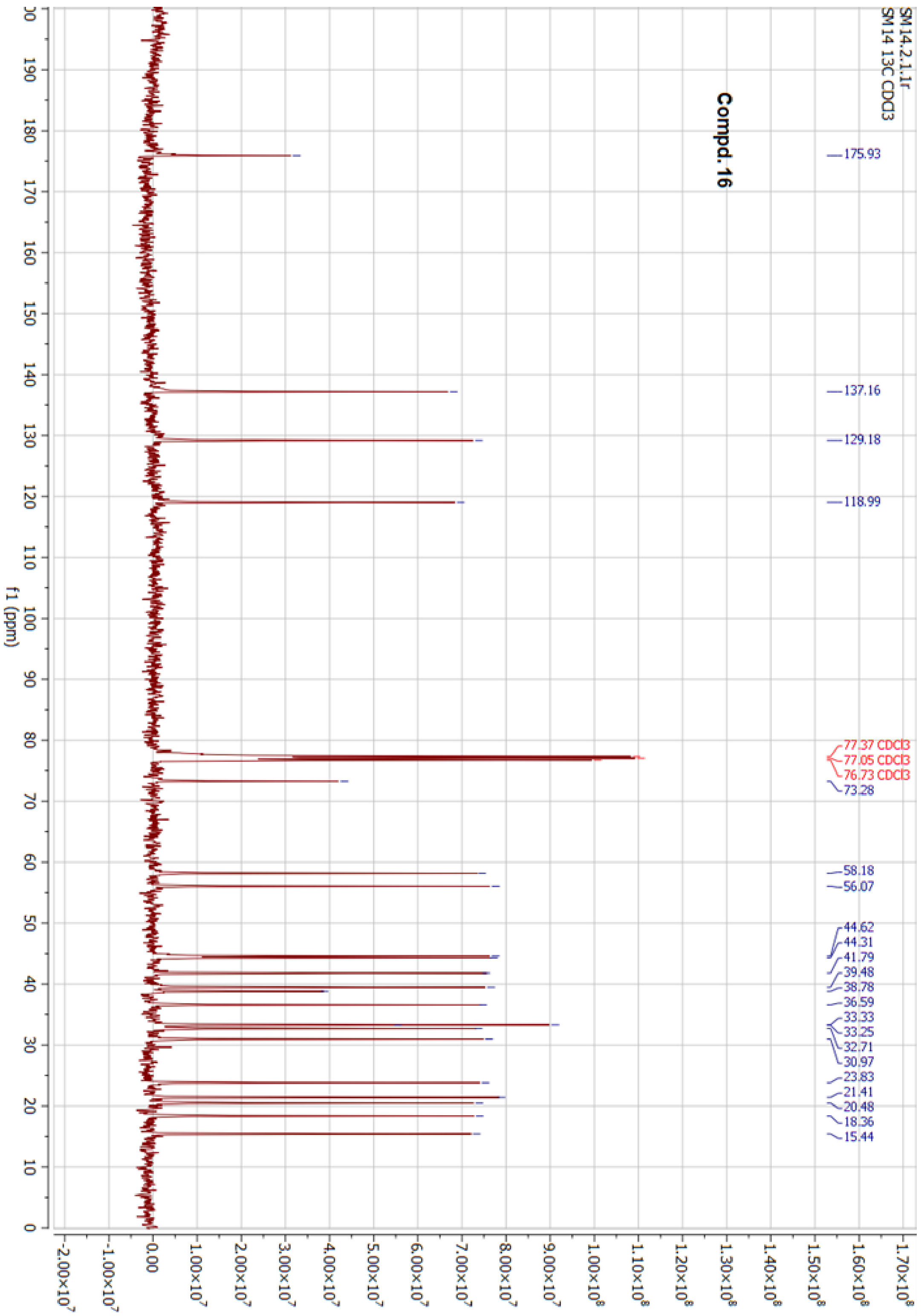
SM14.2.1.1r
SM14 13C CDCl3
Compd. 16
175.93
137.16
129.18
118.99
77.37 CDCl3
77.05 CDCl3
76.73 CDCl3
73.28
58.18
56.07
44.62
44.31
41.79
39.48
38.78
36.59
33.33
33.25
32.71
30.97
23.83
21.41
20.48
18.36
15.44
f1 (ppm)

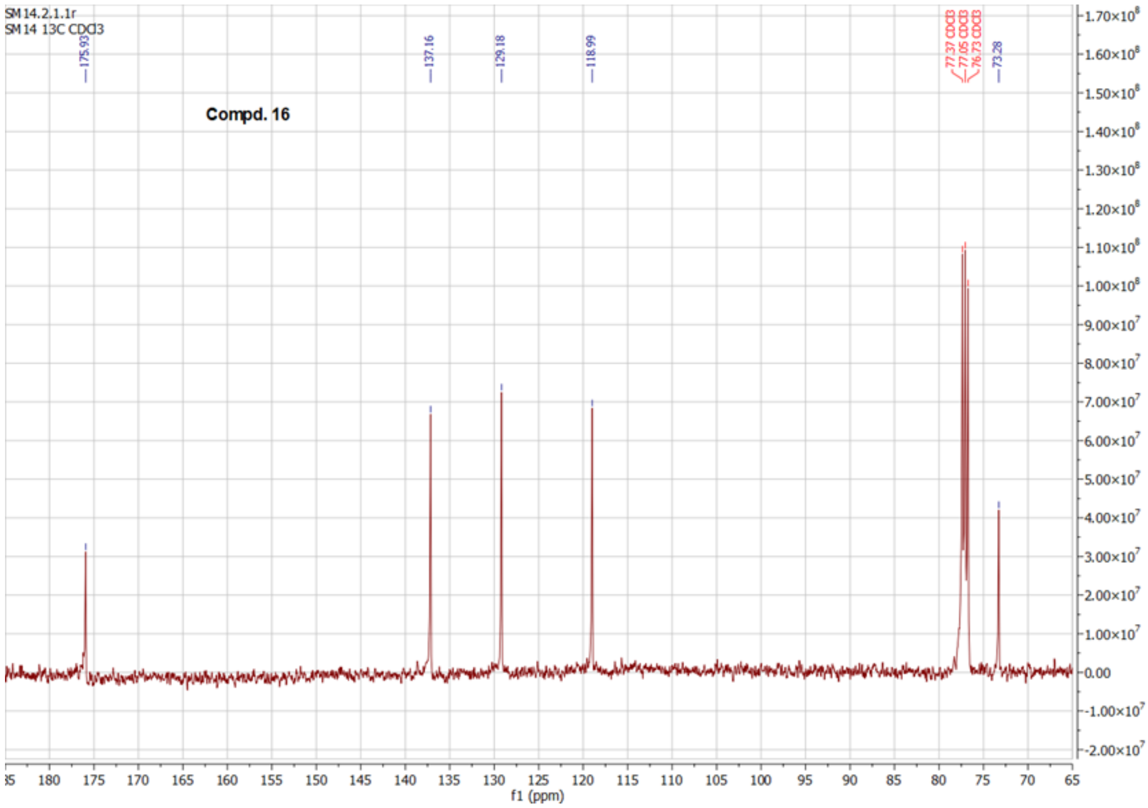

SM14.2.1.1r
SM14 13C CDCl3
Compd. 16
175.93
137.16
129.18
118.99
77.37 CDCl3
77.05 CDCl3
76.73 CDCl3
73.28
f1 (ppm)

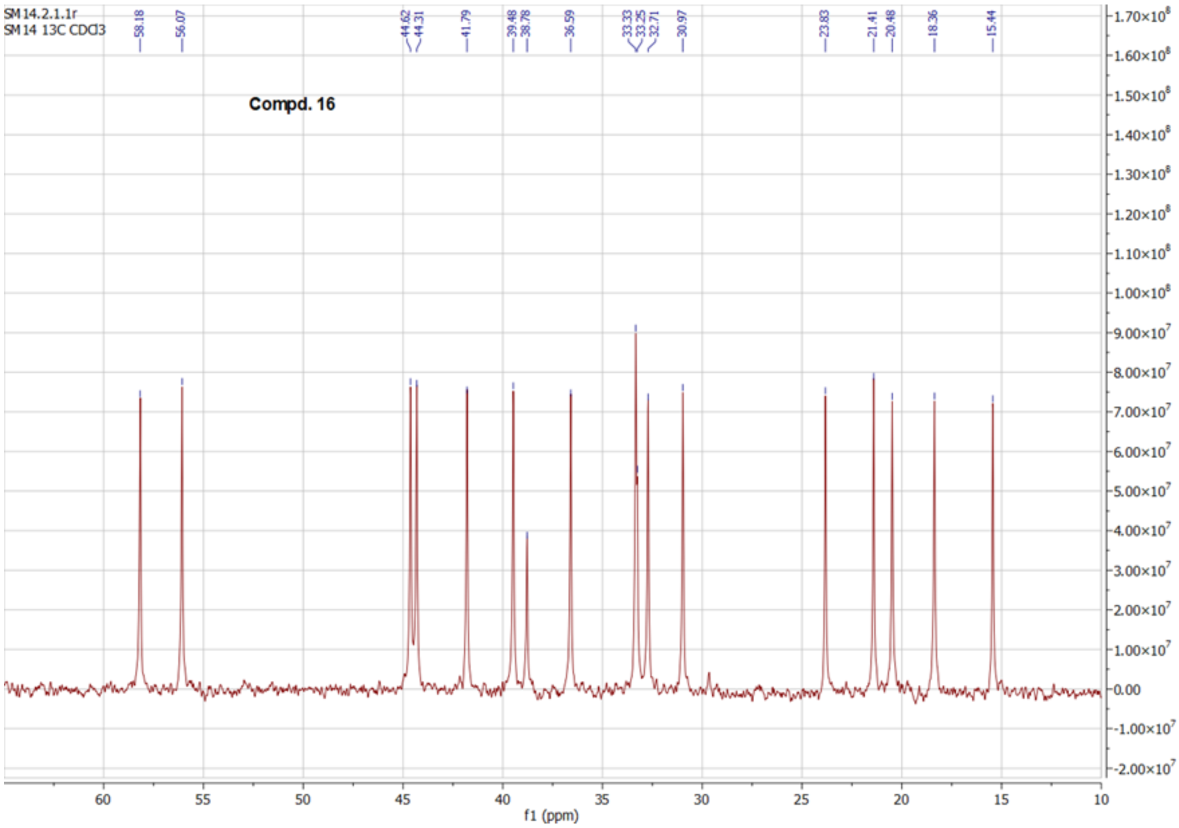

SM14.2.1.1r
SM14 13C CDCl3
Compd. 16
58.18
56.07
44.62
44.31
41.79
39.48
38.78
36.59
33.33
33.25
32.71
30.97
23.83
21.41
20.48
18.36
15.44
f1 (ppm)

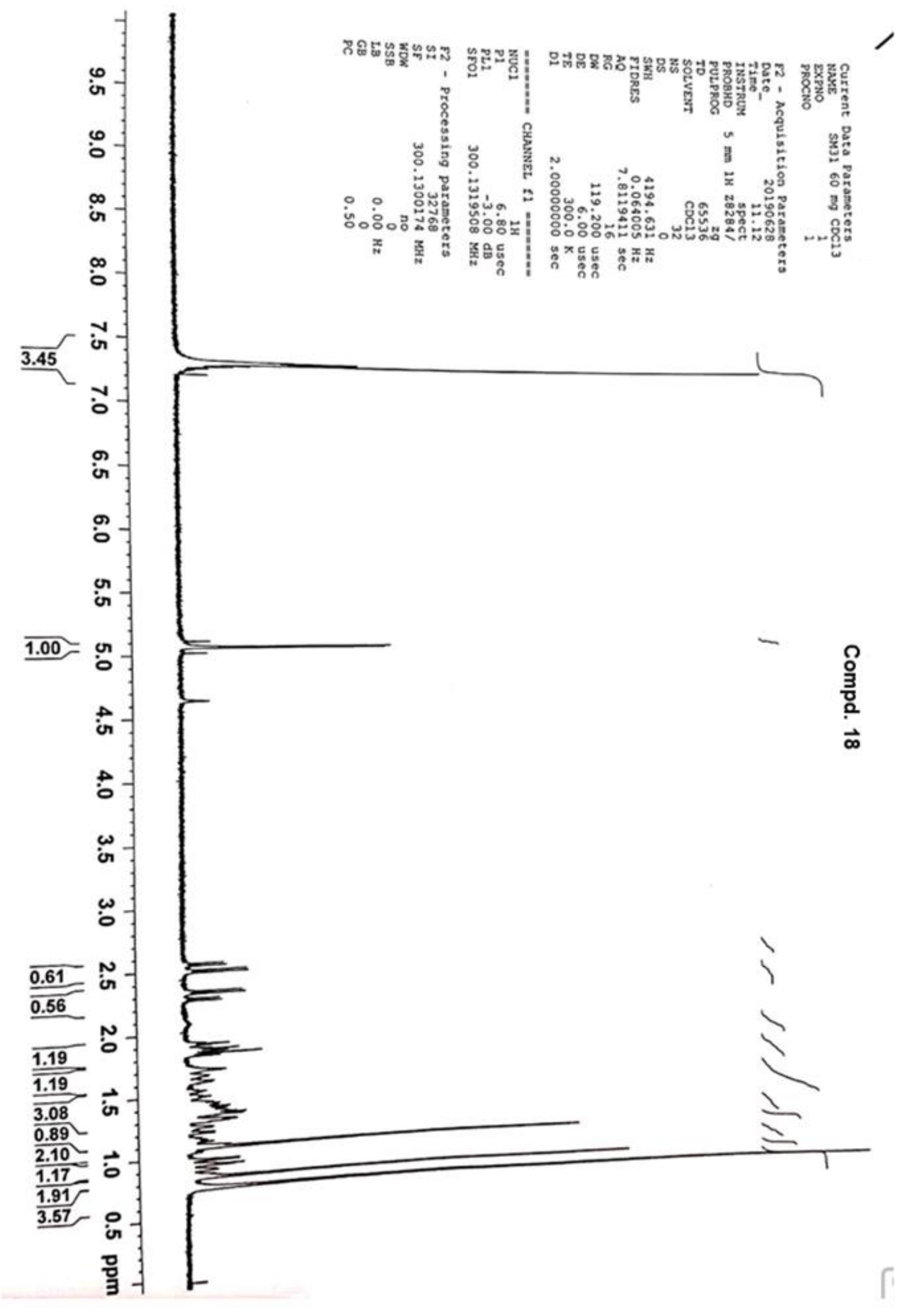
Compd. 18
Current Data Parameters
NAME SM31 60 mg CDC13
EXPNO 1
PROCNO 1
F2 - Acquisition Parameters
Date_ 20190628
Time 11.12
INSTRUM spect
PROBHD 5 mm 1H Z8284/
PULPROG zg
TD 65536
SOLVENT CDC13
NS 32
DS 0
SWH 4194.631 Hz
FIDRES 0.064005 Hz
AQ 7.8119411 sec
RG 16
DW 119.200 usec
DE 6.00 usec
TE 300.0 K
D1 2.00000000 sec
======== CHANNEL f1 ========
NUC1 1H
P1 6.80 usec
PL1 -3.00 dB
SFO1 300.1319508 MHz
F2 - Processing parameters
SI 32768
SF 300.1300174 MHz
WDW no
SSB 0
LB 0.00 Hz
GB 0
PC 0.50
9.5 9.0 8.5 8.0 7.5 7.0 6.5 6.0 5.5 5.0 4.5 4.0 3.5 3.0 2.5 2.0 1.5 1.0 0.5 ppm
3.45
1.00
0.61
0.56
1.19
1.19
3.08
0.89
2.10
1.17
1.91
3.57

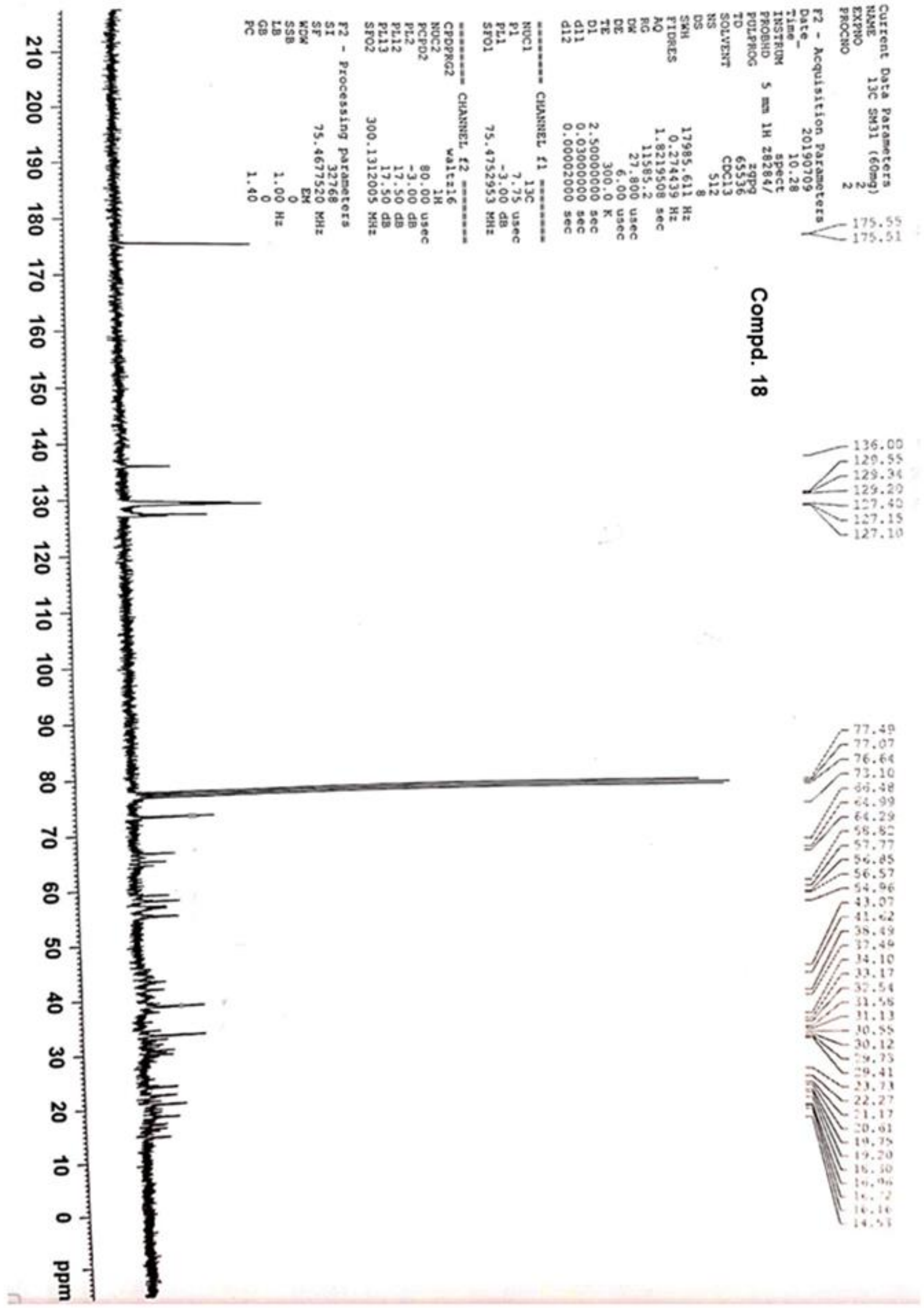
Compd. 18
ppm

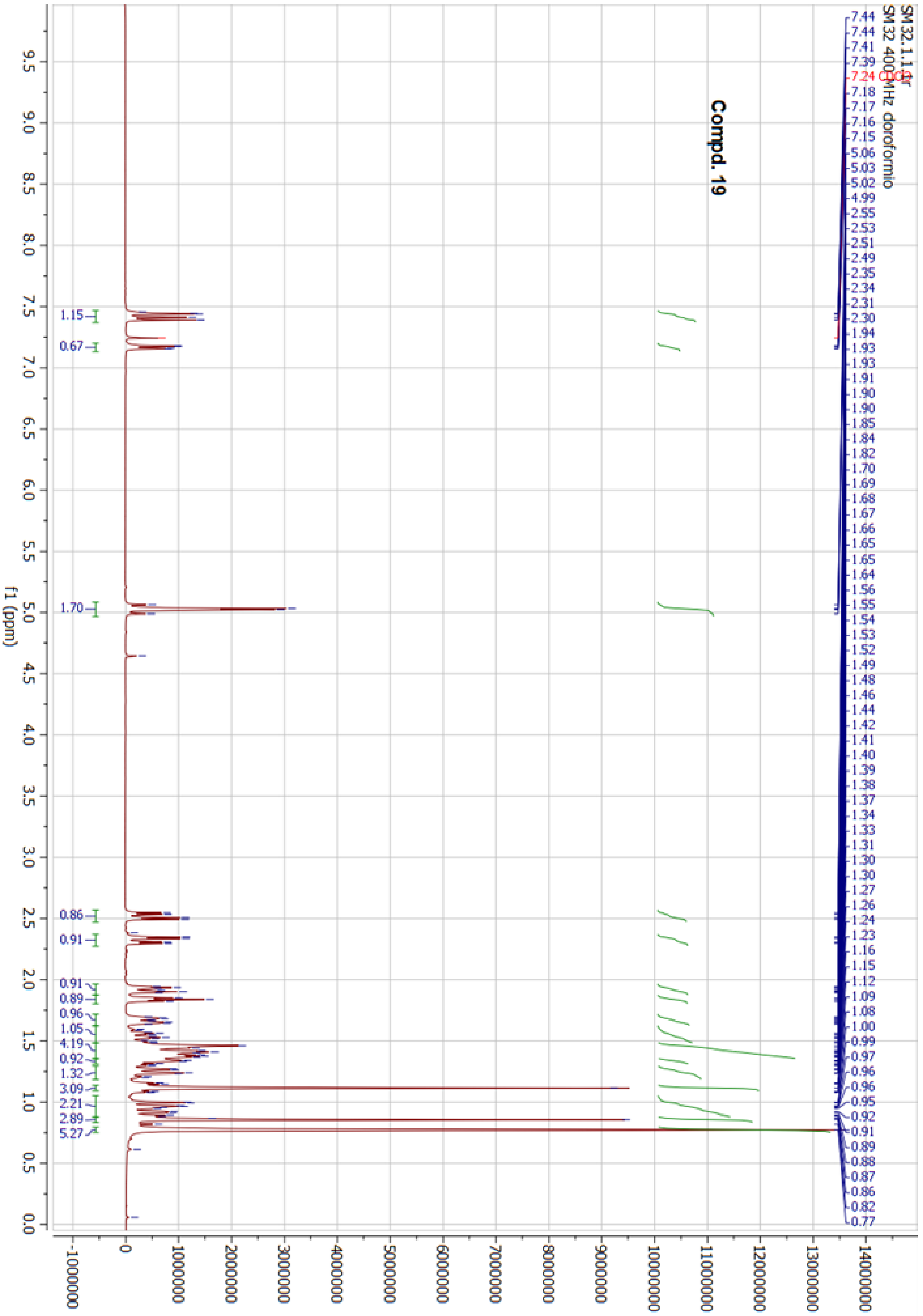
Compd. 19
f1 (ppm)

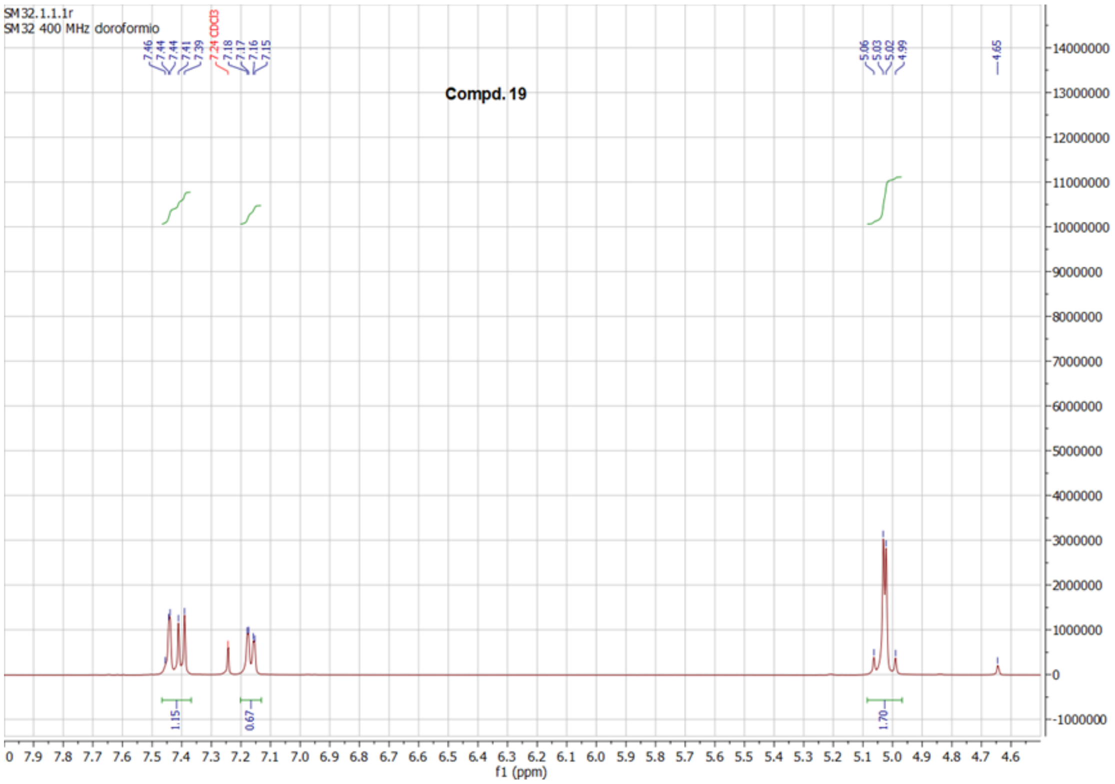
SM32.1.1.1r
SM32 400 MHz cloroformio
Compd. 19
f1 (ppm)

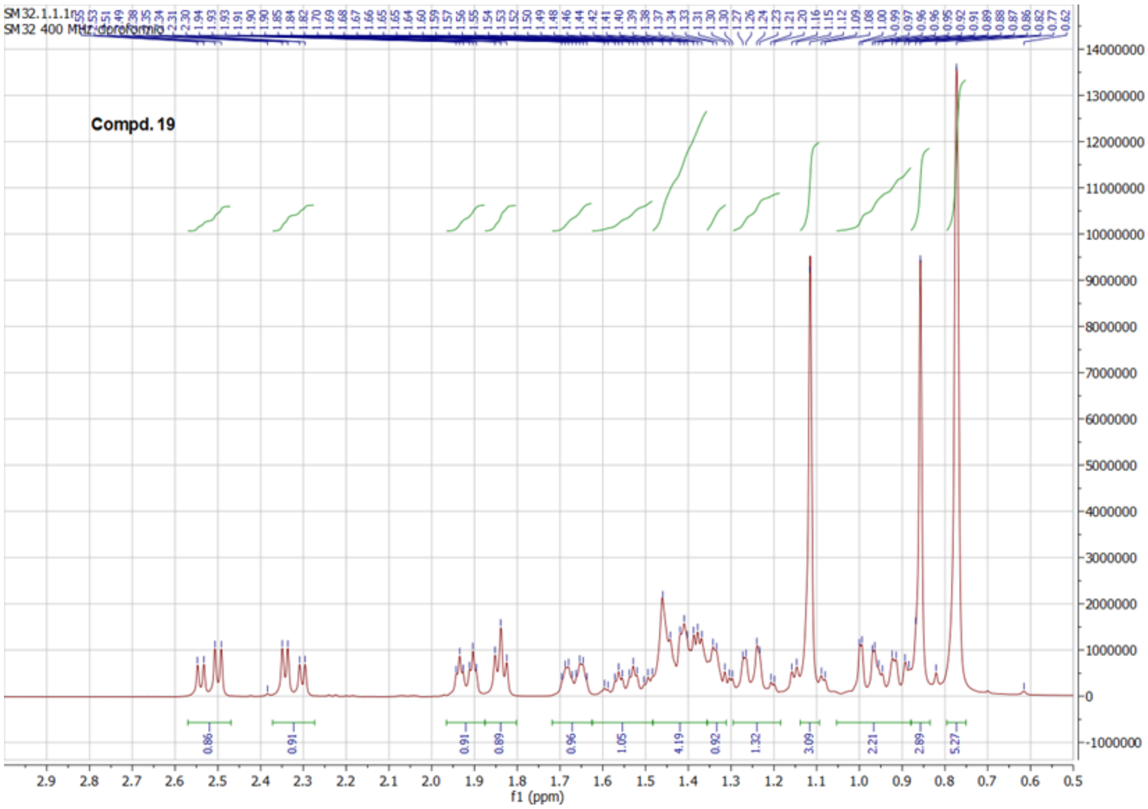
SM32.1.1.1r
SM32 400 MHz cloroformio
Compd. 19
f1 (ppm)

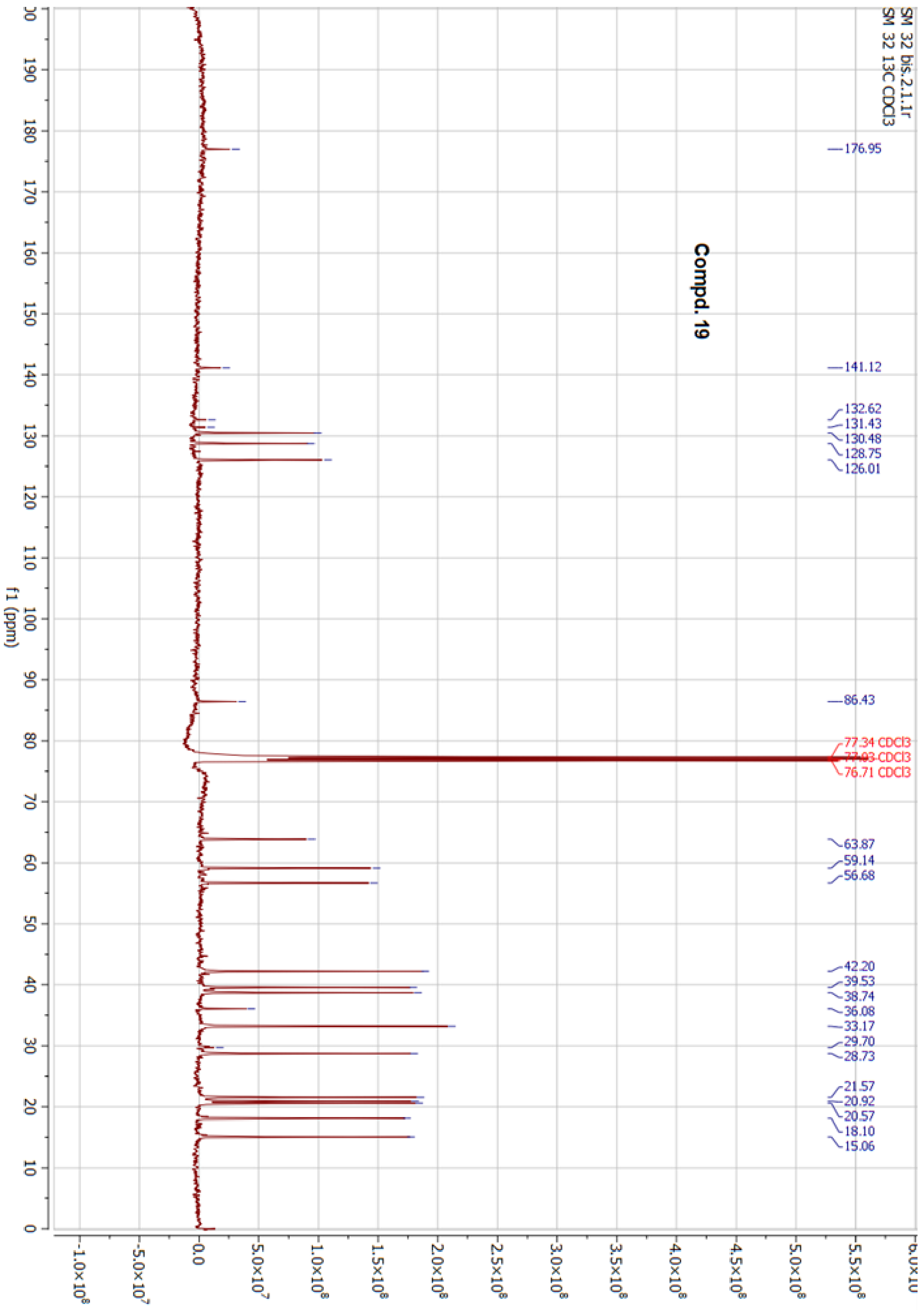
SM 32 bis.2.1.1r
SM 32 13C CDCl3
Compd. 19
176.95
141.12
132.62
131.43
130.48
128.75
126.01
86.43
77.34 CDCl3
77.03 CDCl3
76.71 CDCl3
63.87
59.14
56.68
42.20
39.53
38.74
36.08
33.17
29.70
28.73
21.57
20.92
20.57
18.10
15.06
f1 (ppm)

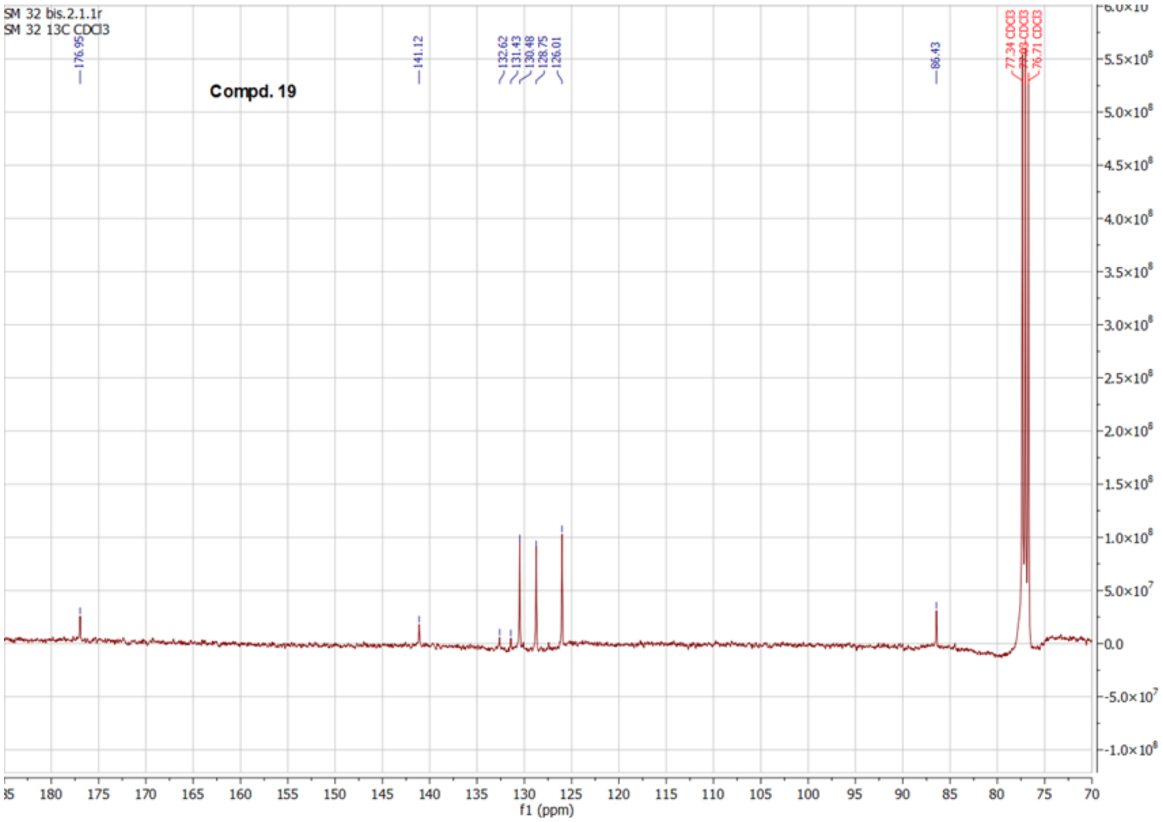
SM 32 bis.2.1.1r
SM 32 13C CDCl3
Compd. 19
176.95
141.12
132.62
131.43
130.48
128.75
126.01
86.43
77.34 CDCl3
77.03 CDCl3
76.71 CDCl3
f1 (ppm)

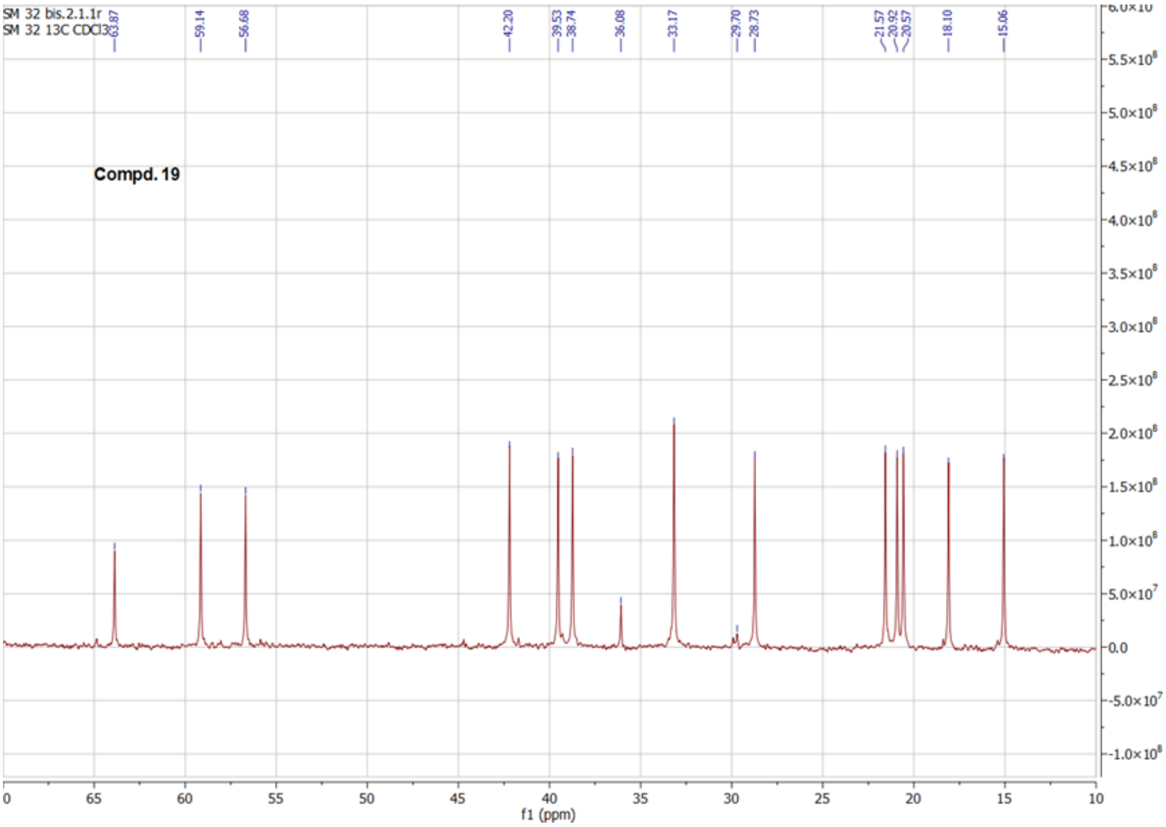
SM 32 bis.2.1.1r
SM 32 13C CDCl3
Compd. 19
63.87
59.14
56.68
42.20
39.53
38.74
36.08
33.17
29.70
28.73
21.57
20.92
20.57
18.10
15.06
f1 (ppm)

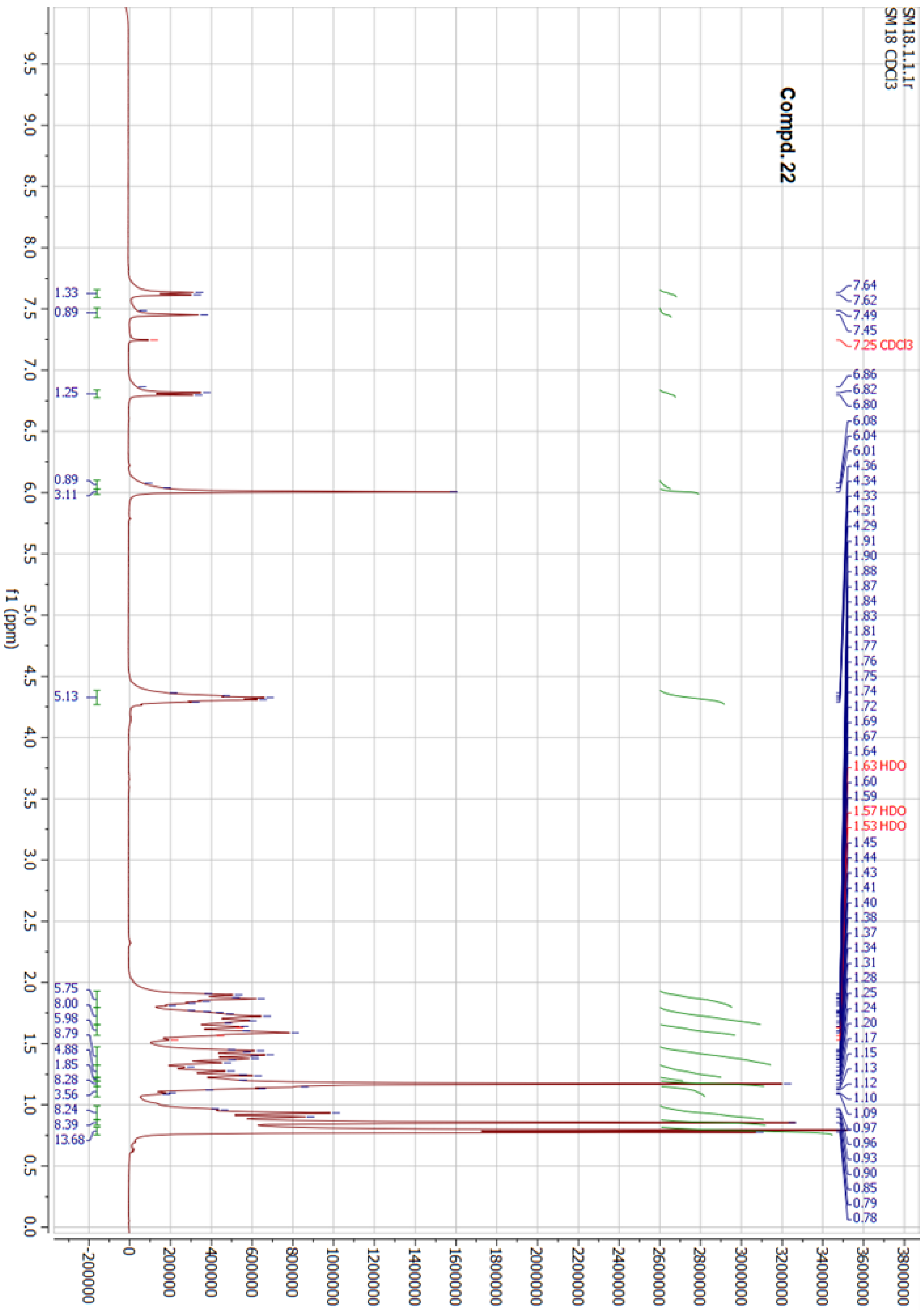
SM18.1.1.1r
SM18 CDCl3
Compd. 22
f1 (ppm)
7.25 CDCl3
1.63 HDO
1.57 HDO
1.53 HDO

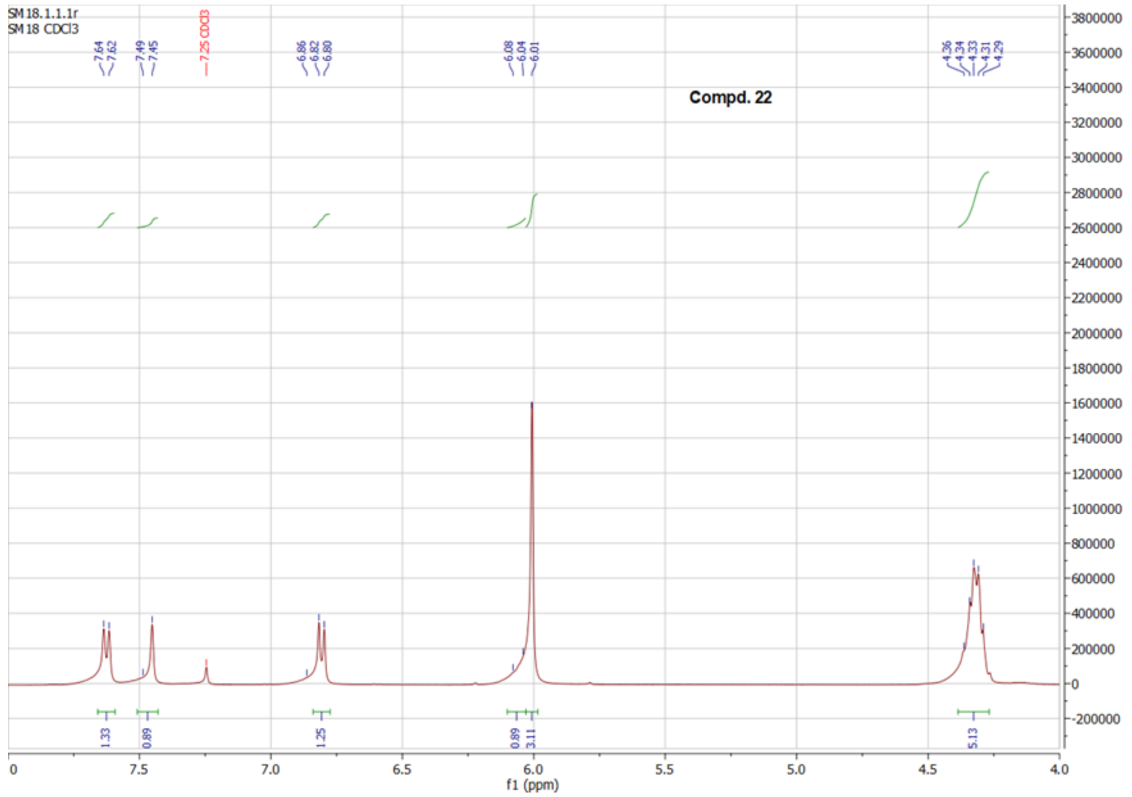
SM18.1.1.1r
SM18 CDCl3
Compd. 22
f1 (ppm)

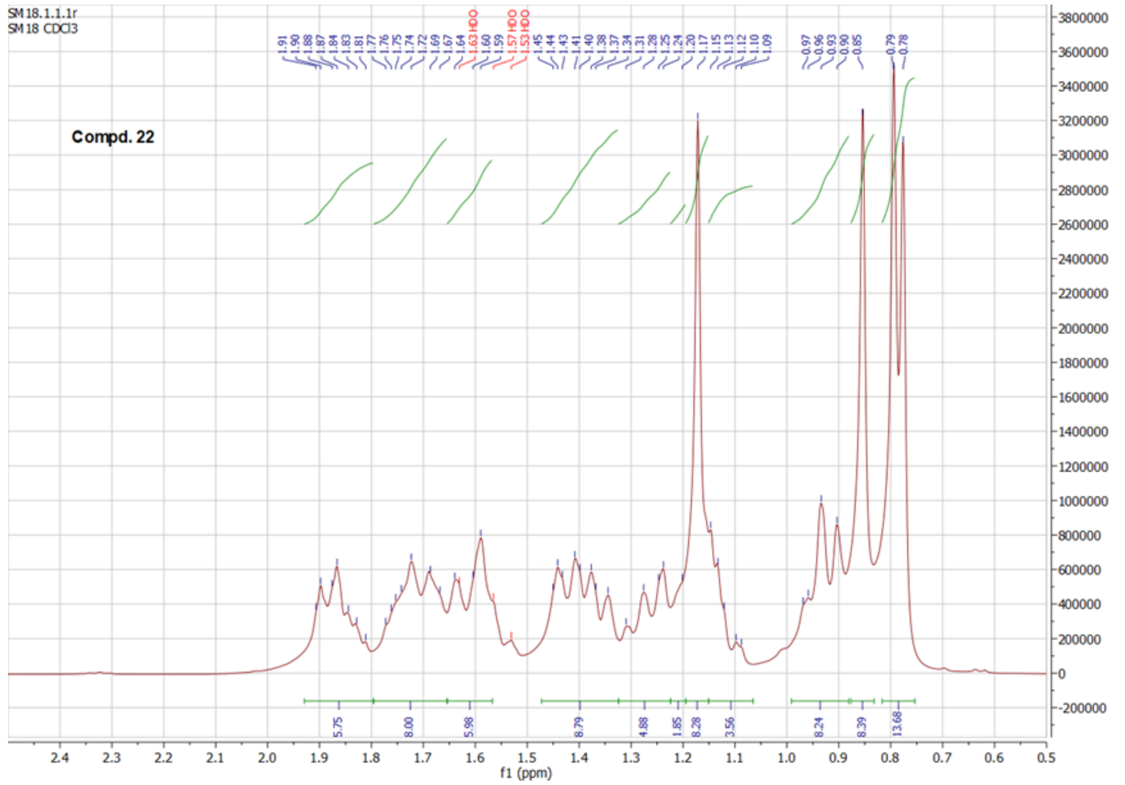
SM18.1.1.1r
SM18 CDCl3
Compd. 22
f1 (ppm)

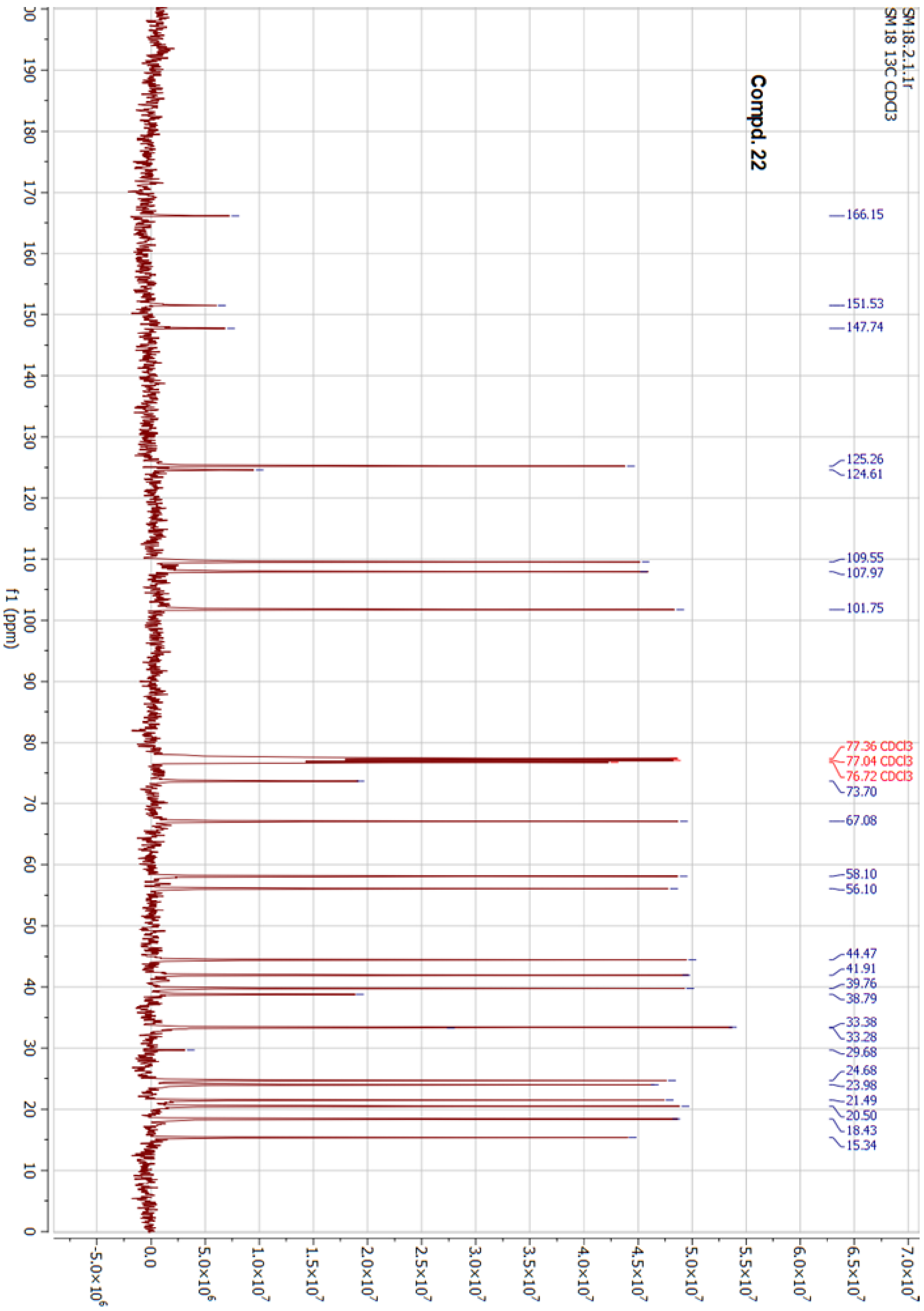
SM18.2.1.1r
SM 18 13C CDCl3
Compd. 22
166.15
151.53
147.74
125.26
124.61
109.55
107.97
101.75
77.36 CDCl3
77.04 CDCl3
76.72 CDCl3
73.70
67.08
58.10
56.10
44.47
41.91
39.76
38.79
33.38
33.28
29.68
24.68
23.98
21.49
20.50
18.43
15.34
f1 (ppm)

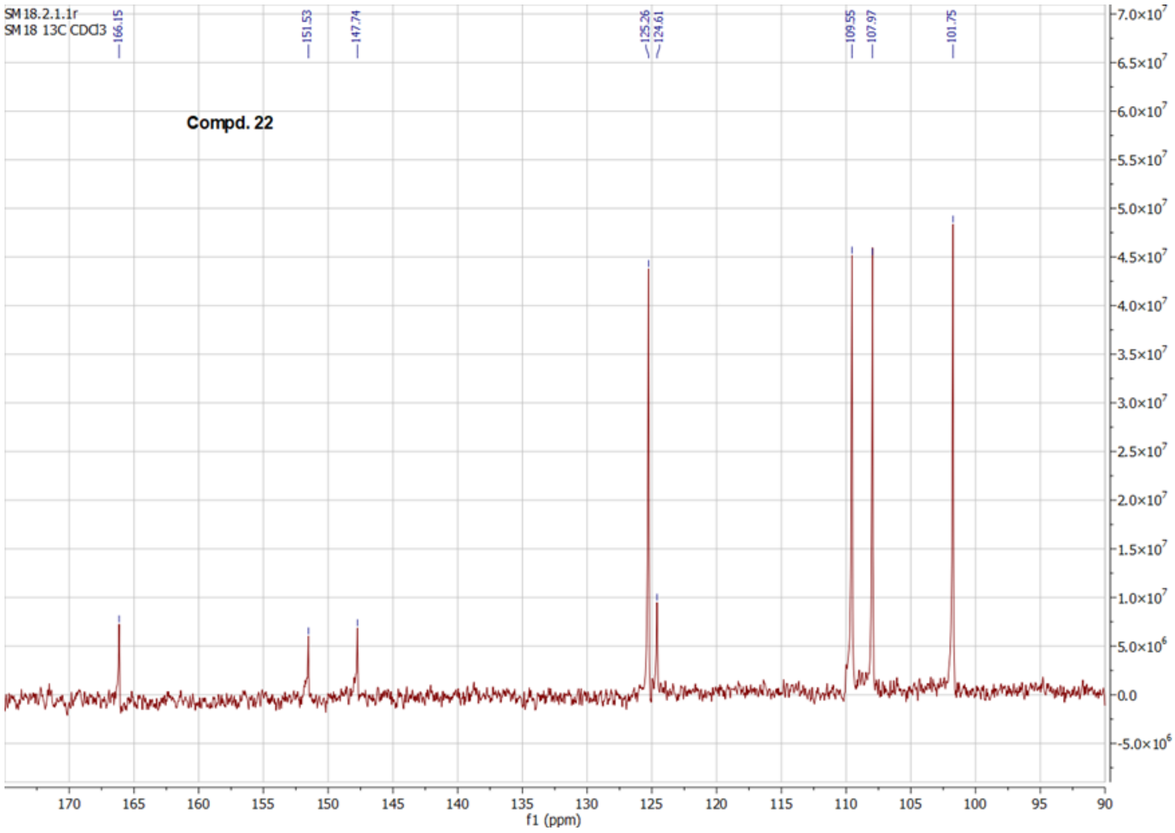
SM18.2.1.1r
SM18 13C CDCl3
Compd. 22
166.15
151.53
147.74
125.26
124.61
109.55
107.97
101.75
f1 (ppm)

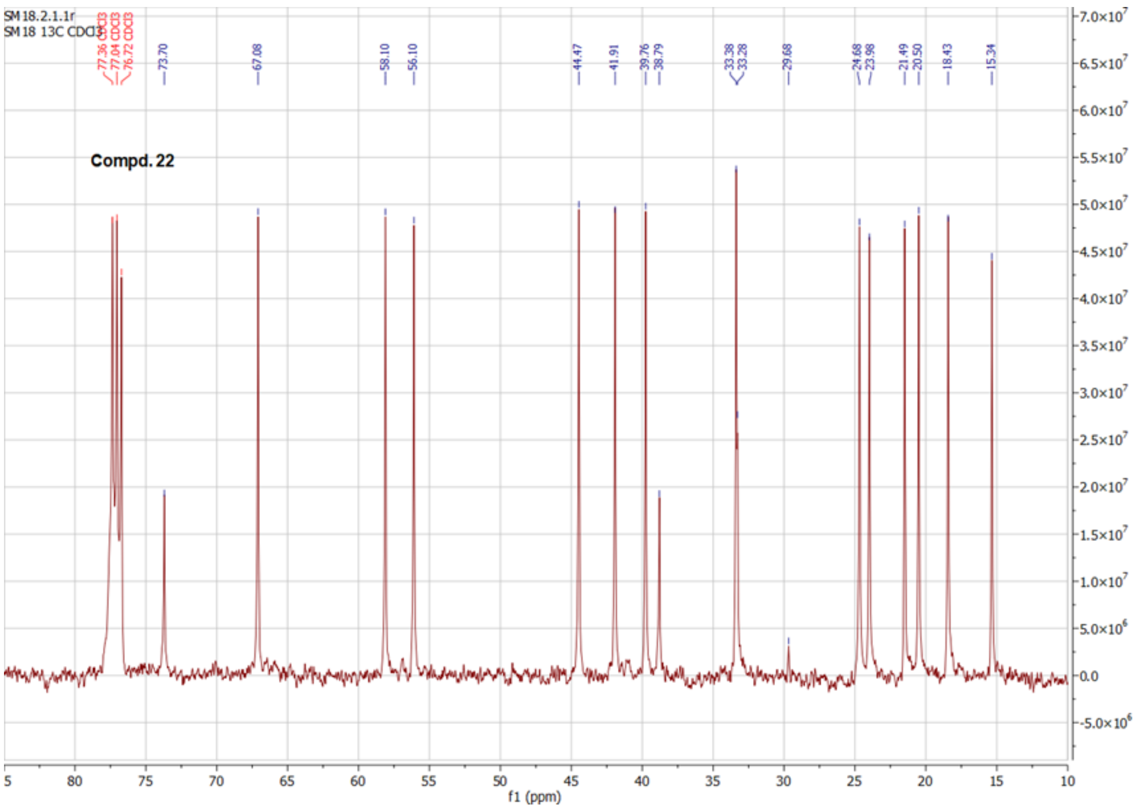
SM18.2.1.1r
SM18 13C CDCl3
Compd. 22
77.36 CDCl3
77.04 CDCl3
76.72 CDCl3
73.70
67.08
58.10
56.10
44.47
41.91
39.76
38.79
33.38
33.28
29.68
24.68
23.98
21.49
20.50
18.43
15.34
f1 (ppm)

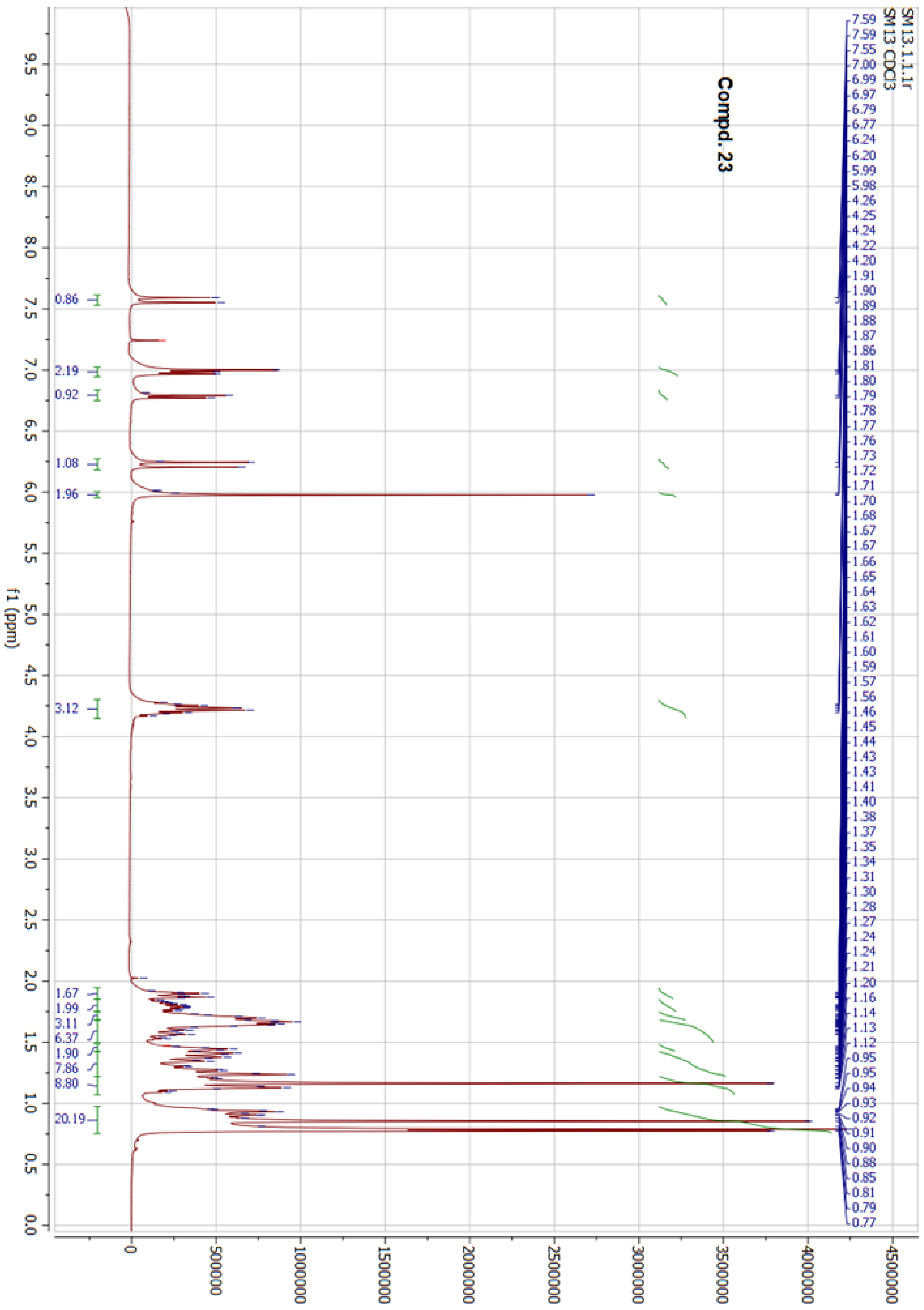
SM13.1.1.1r
SM13 CDCl3
Compd. 23
7.59
7.59
7.55
7.00
6.99
6.97
6.79
6.77
6.24
6.20
5.99
5.98
4.26
4.25
4.24
4.22
4.20
1.91
1.90
1.89
1.88
1.87
1.86
1.81
1.80
1.79
1.78
1.77
1.76
1.73
1.72
1.71
1.70
1.68
1.67
1.67
1.66
1.65
1.64
1.63
1.62
1.61
1.60
1.59
1.57
1.56
1.46
1.45
1.44
1.43
1.43
1.41
1.40
1.38
1.37
1.35
1.34
1.31
1.30
1.28
1.27
1.24
1.24
1.21
1.20
1.16
1.14
1.13
1.12
0.95
0.95
0.94
0.93
0.92
0.91
0.90
0.88
0.85
0.81
0.79
0.77
0.86
2.19
0.92
1.08
1.96
3.12
1.67
1.99
3.11
6.37
1.90
7.86
8.80
20.19
f1 (ppm)

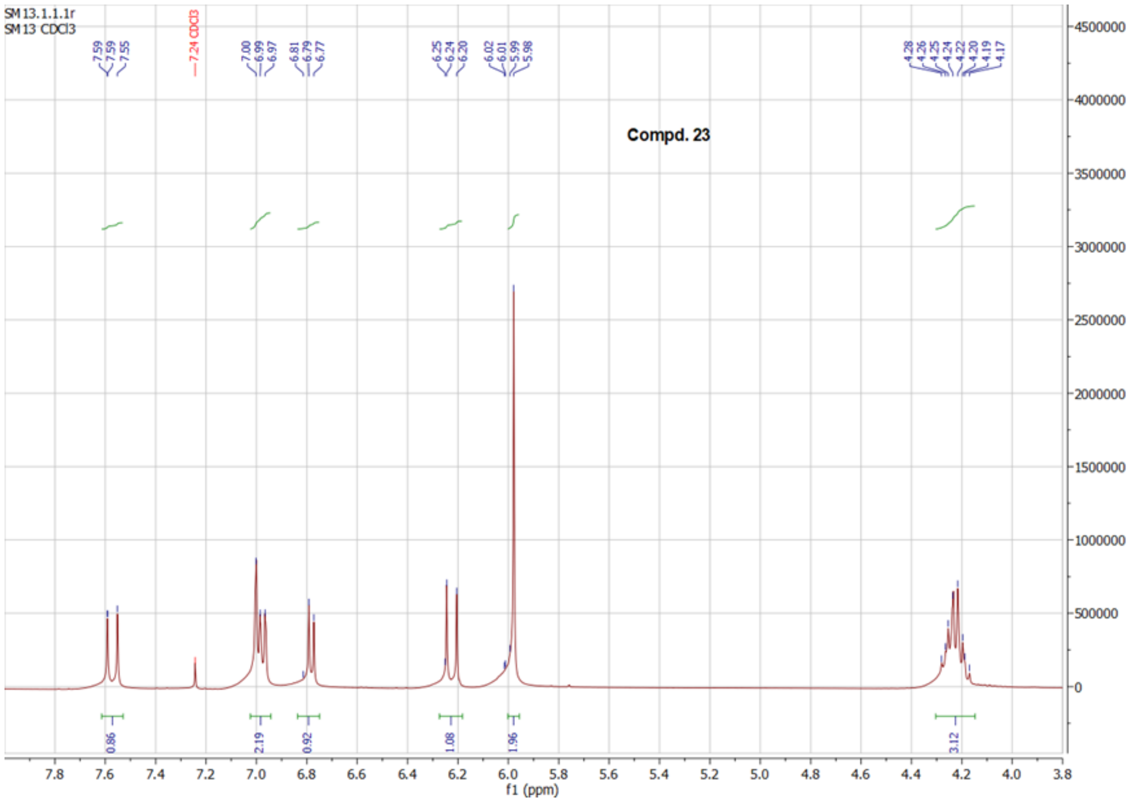
SM13.1.1.1r
SM13 CDCl3
Compd. 23
f1 (ppm)

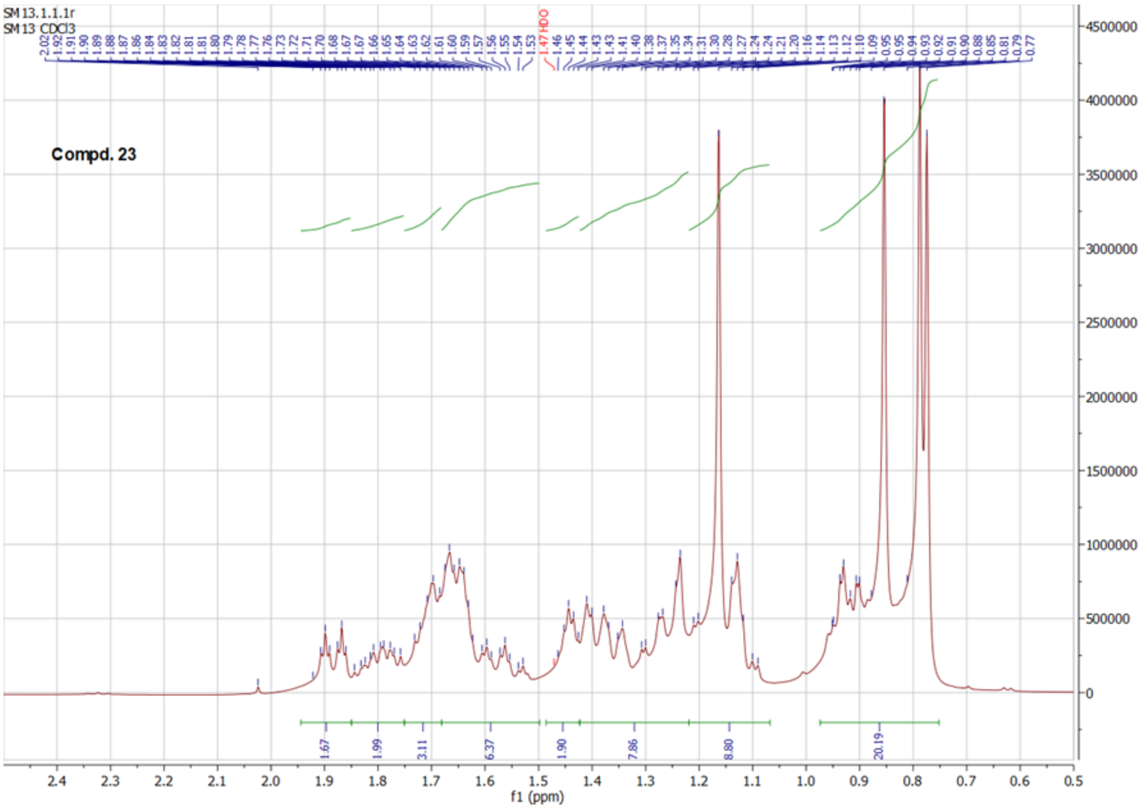
SM13.1.1.1r
SM13 CDCl3
Compd. 23
f1 (ppm)

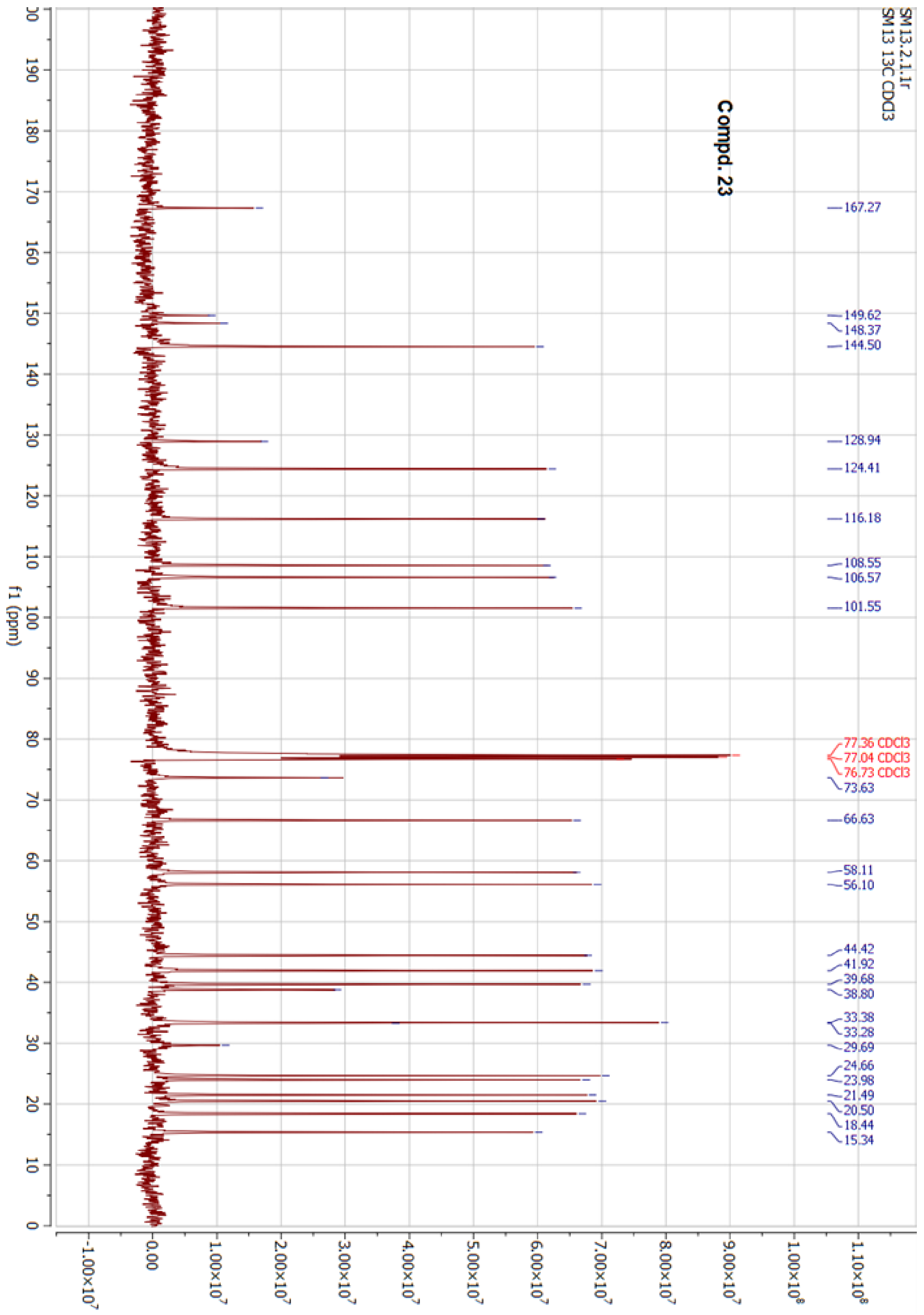
SM13.2.1.1r
SM13 13C CDCl3
Compd. 23
167.27
149.62
148.37
144.50
128.94
124.41
116.18
108.55
106.57
101.55
77.36 CDCl3
77.04 CDCl3
76.73 CDCl3
73.63
66.63
58.11
56.10
44.42
41.92
39.68
38.80
33.38
33.28
29.69
24.66
23.98
21.49
20.50
18.44
15.34
f1 (ppm)

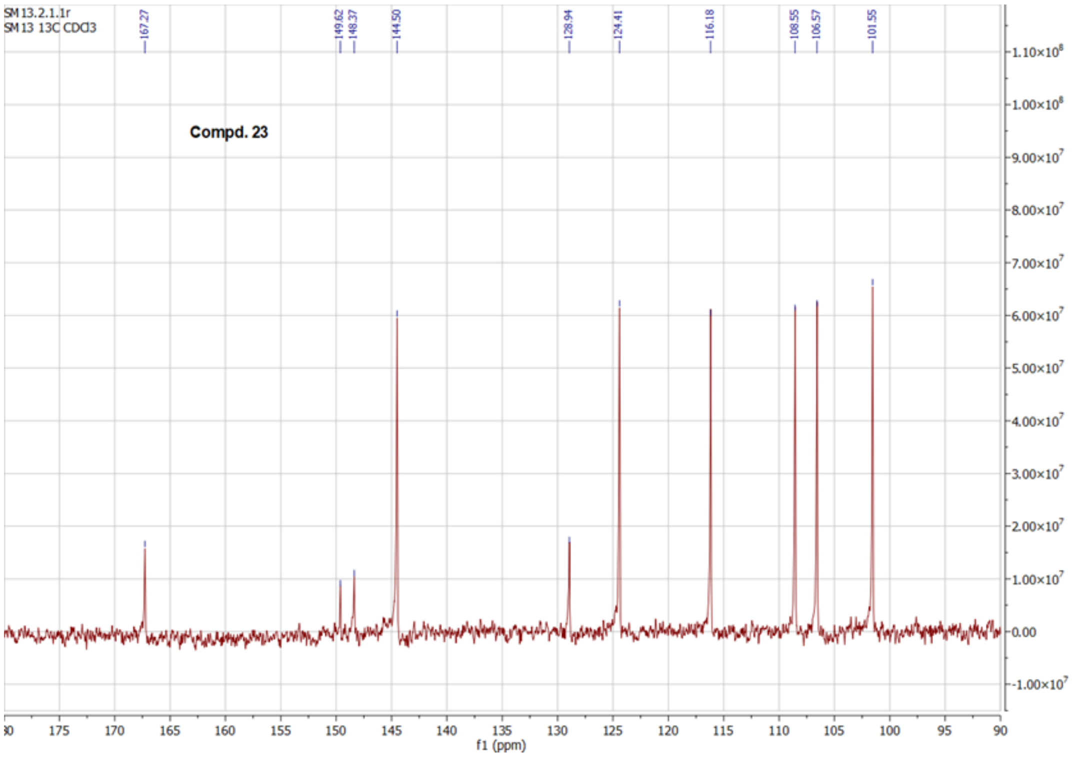
SM13.2.1.1r
SM13 13C CDCl3
Compd. 23
167.27
149.62
148.37
144.50
128.94
124.41
116.18
108.55
106.57
101.55
f1 (ppm)

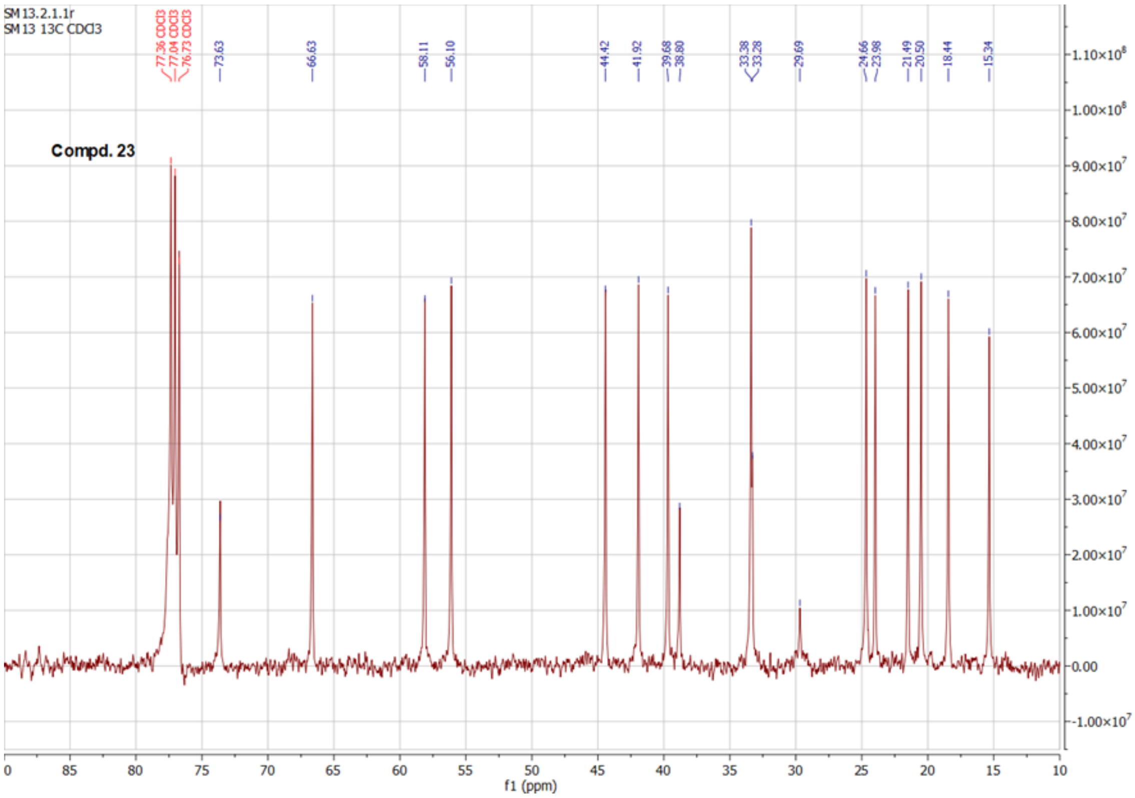
SM13.2.1.1r
SM13 13C CDCl3
Compd. 23
77.36 CDCl3
77.04 CDCl3
76.73 CDCl3
73.63
66.63
58.11
56.10
44.42
41.92
39.68
38.80
33.38
33.28
29.69
24.66
23.98
21.49
20.50
18.44
15.34
f1 (ppm)

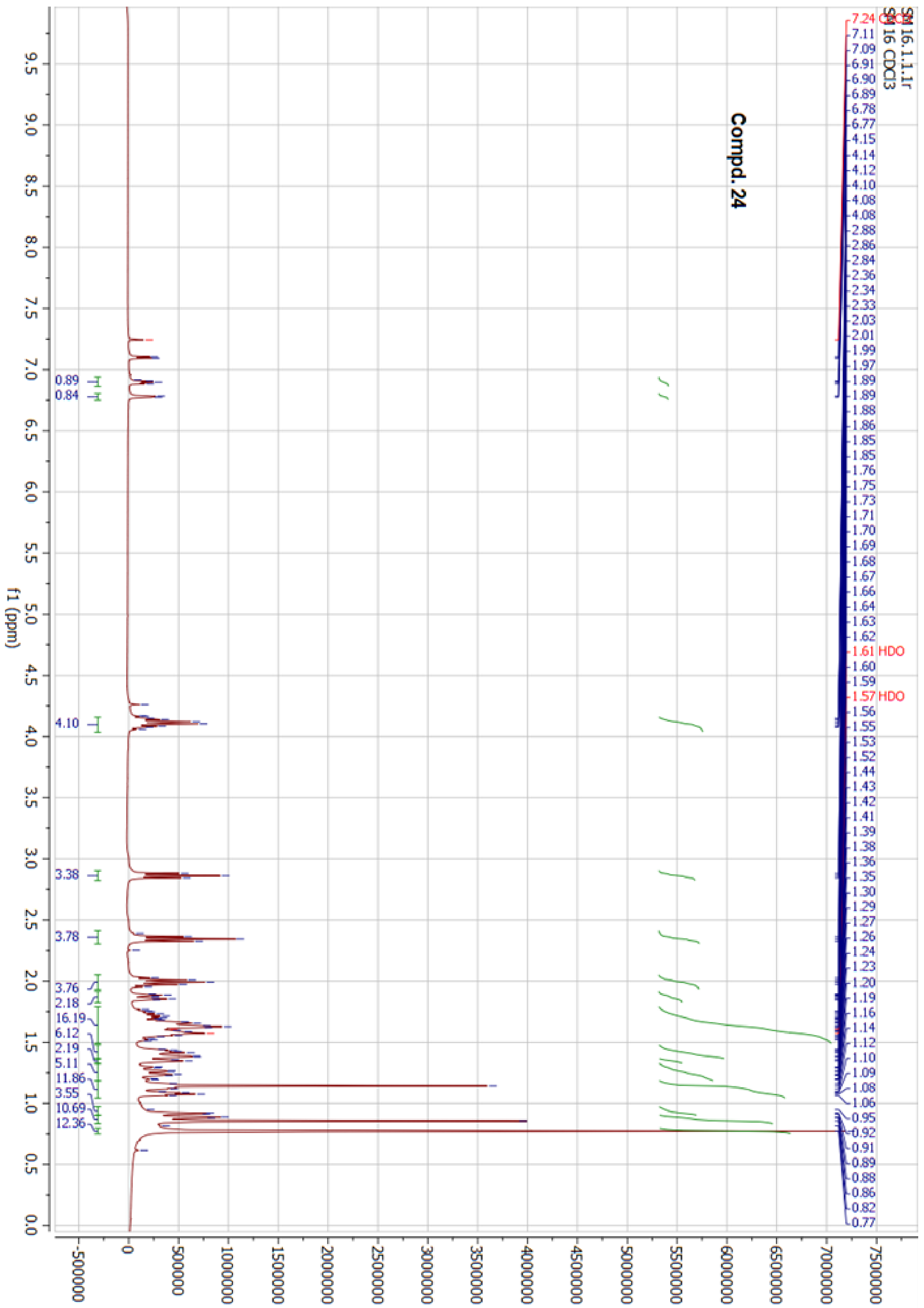
SJ 16.1.1.1r
SJ 16 CDCl3
Compd. 24
f1 (ppm)

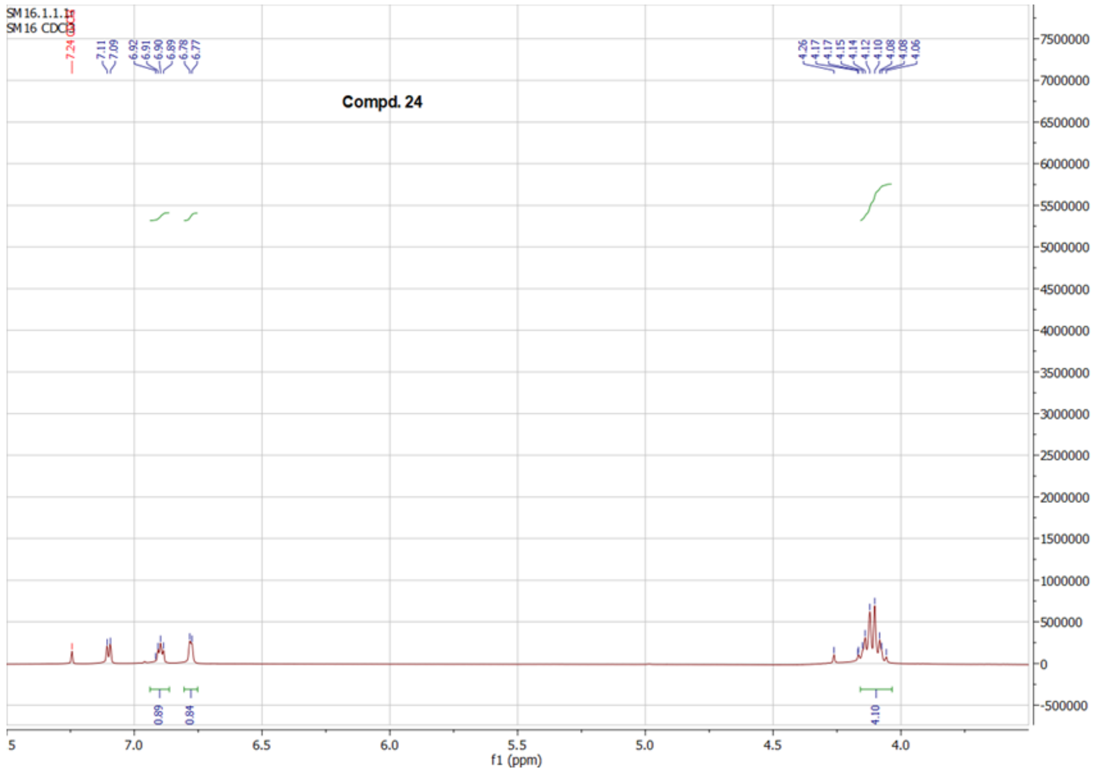
SM16.1.1.1r
SM16 CDCl3
7.24 CDCl3
7.11
7.09
6.92
6.91
6.90
6.89
6.78
6.77
4.26
4.17
4.17
4.15
4.14
4.12
4.10
4.08
4.08
4.06
Compd. 24
0.89
0.84
4.10
f1 (ppm)

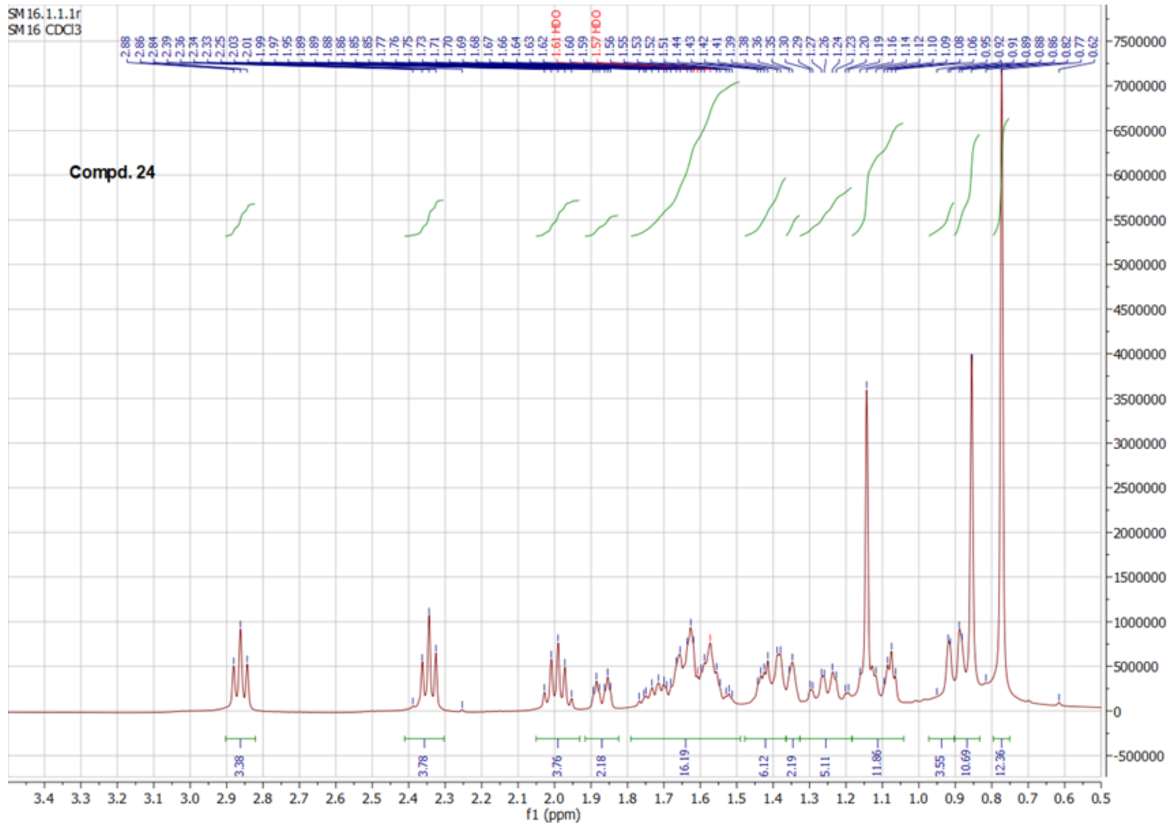
SM16.1.1.1r
SM16 CDCl3
2.88
2.86
2.84
2.39
2.36
2.34
2.33
2.25
2.03
2.01
1.99
1.97
1.95
1.89
1.89
1.88
1.86
1.85
1.85
1.77
1.76
1.75
1.73
1.71
1.70
1.69
1.68
1.67
1.66
1.64
1.63
1.62
1.61 HDO
1.60
1.59
1.57 HDO
1.56
1.55
1.53
1.52
1.51
1.44
1.43
1.42
1.41
1.39
1.38
1.36
1.35
1.30
1.29
1.27
1.26
1.24
1.23
1.20
1.19
1.16
1.14
1.12
1.10
1.09
1.08
1.06
0.95
0.92
0.91
0.89
0.88
0.86
0.82
0.77
0.62
Compd. 24
3.38
3.78
3.76
2.18
16.19
6.12
2.19
5.11
11.86
3.55
10.69
12.36
f1 (ppm)

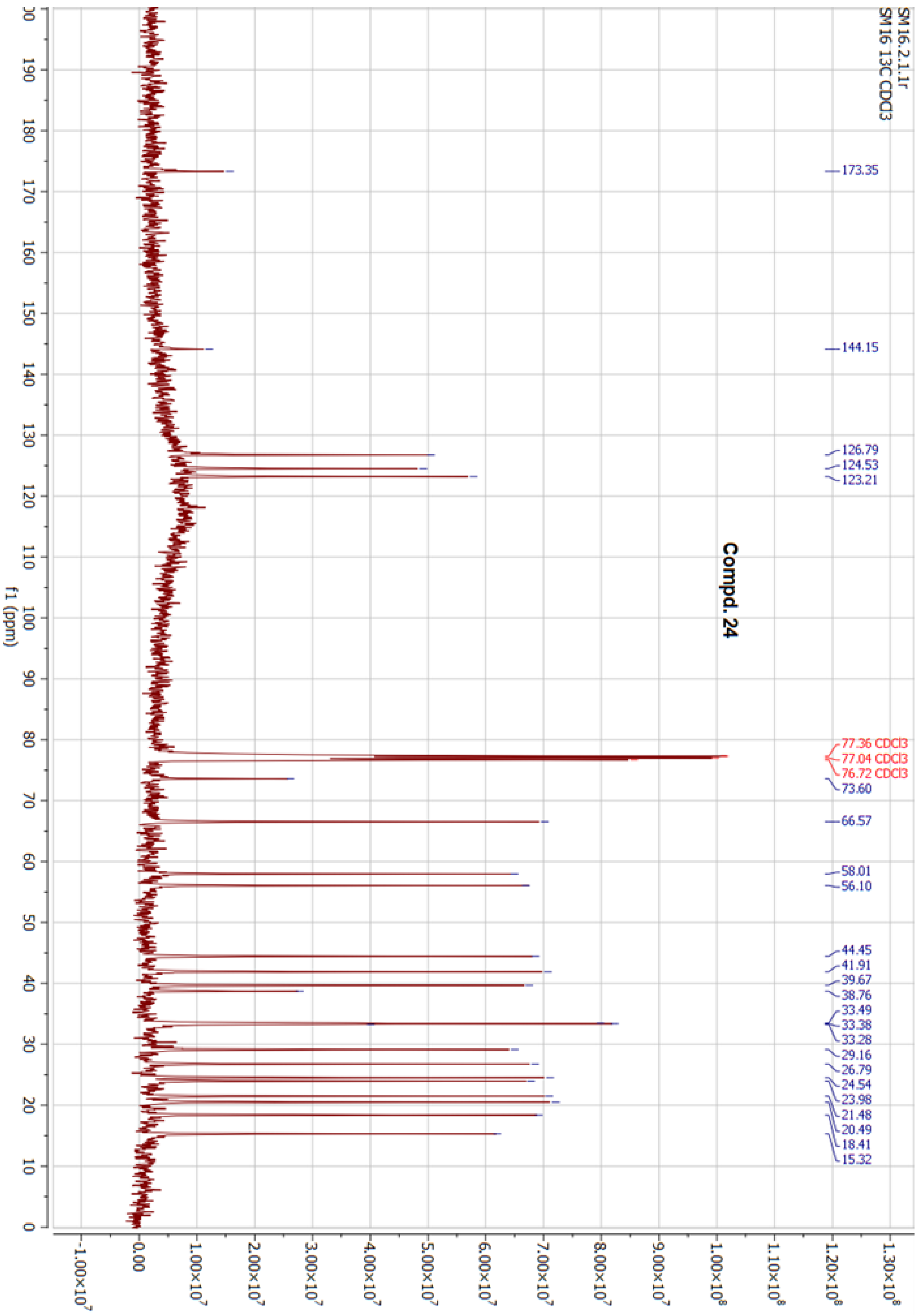
SM16.2.1.1r
SM16 13C CDCl3
Compd. 24
173.35
144.15
126.79
124.53
123.21
77.36 CDCl3
77.04 CDCl3
76.72 CDCl3
73.60
66.57
58.01
56.10
44.45
41.91
39.67
38.76
33.49
33.38
33.28
29.16
26.79
24.54
23.98
21.48
20.49
18.41
15.32
f1 (ppm)

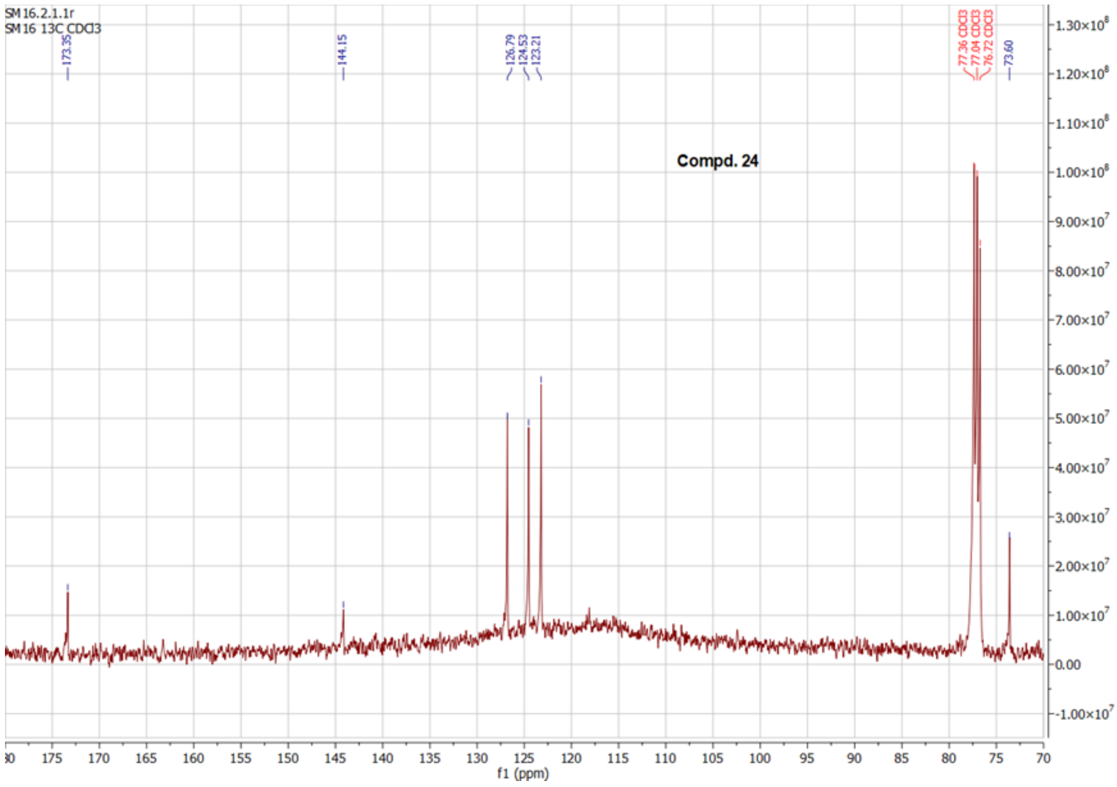
SM16.2.1.1r
SM16 13C CDCl3
173.35
144.15
126.79
124.53
123.21
77.36 CDCl3
77.04 CDCl3
76.72 CDCl3
73.60
Compd. 24
f1 (ppm)

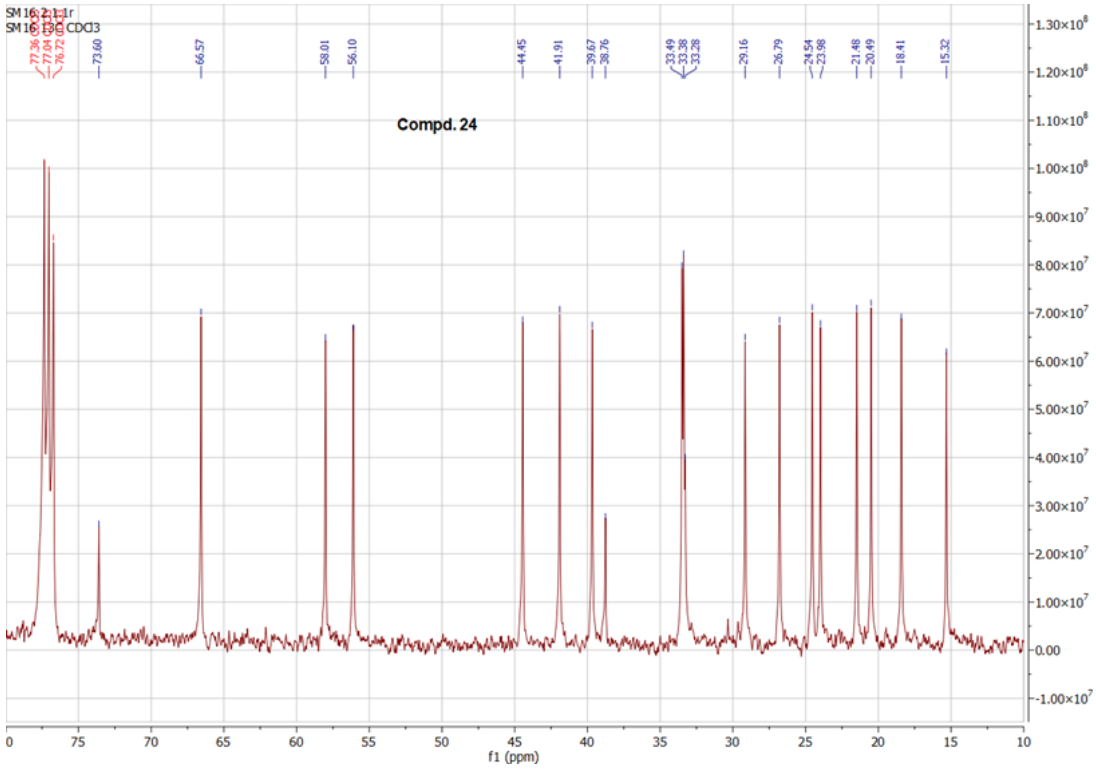
SM16.2.1.1r
SM16 13C CDCl3
77.36
77.04
76.72
73.60
66.57
58.01
56.10
44.45
41.91
39.67
38.76
33.49
33.38
33.28
29.16
26.79
24.54
23.98
21.48
20.49
18.41
15.32
Compd. 24
f1 (ppm)

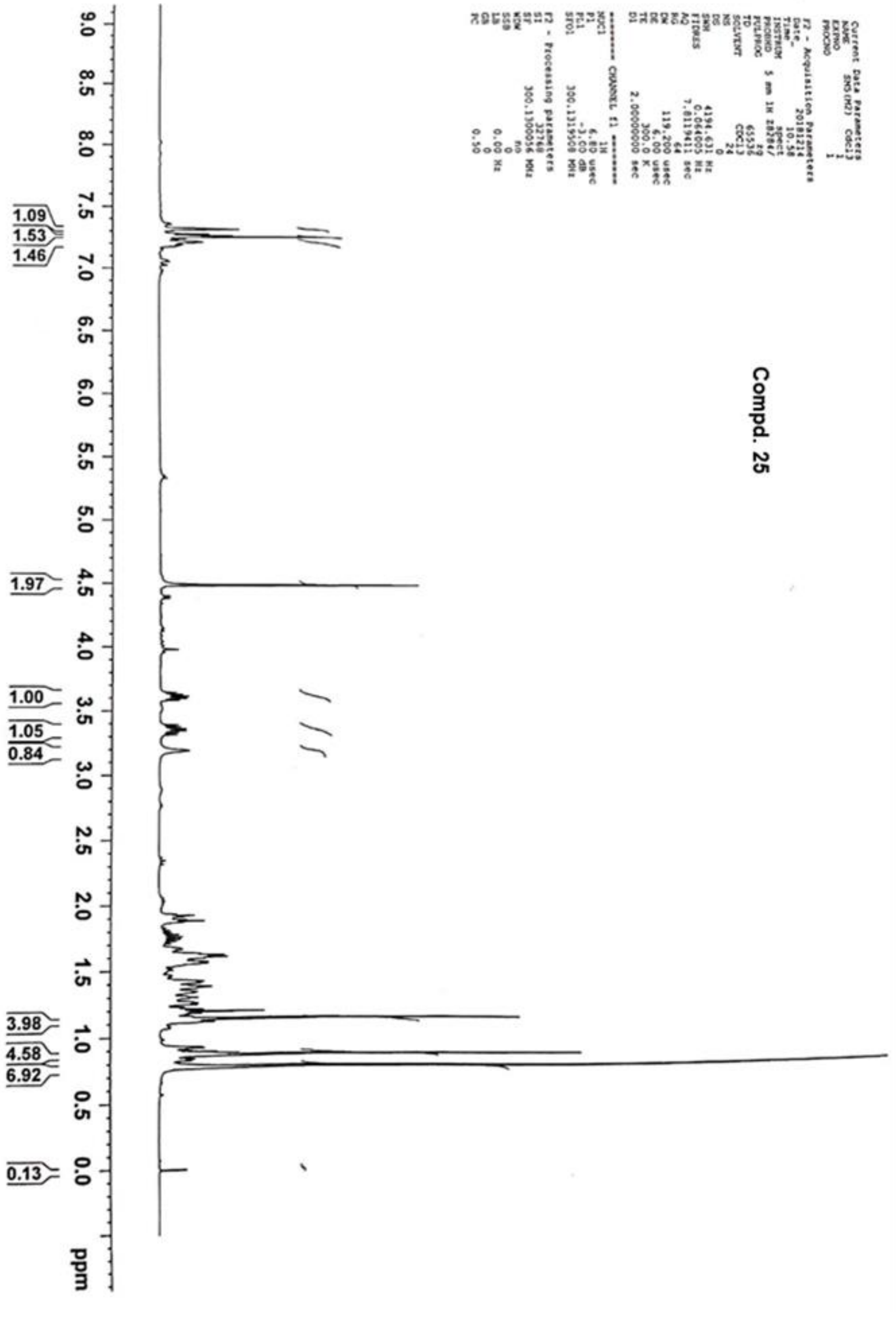
Compd. 25
9.0
8.5
8.0
7.5
7.0
6.5
6.0
5.5
5.0
4.5
4.0
3.5
3.0
2.5
2.0
1.5
1.0
0.5
0.0
ppm
1.09
1.53
1.46
1.97
1.00
1.05
0.84
3.98
4.58
6.92
0.13

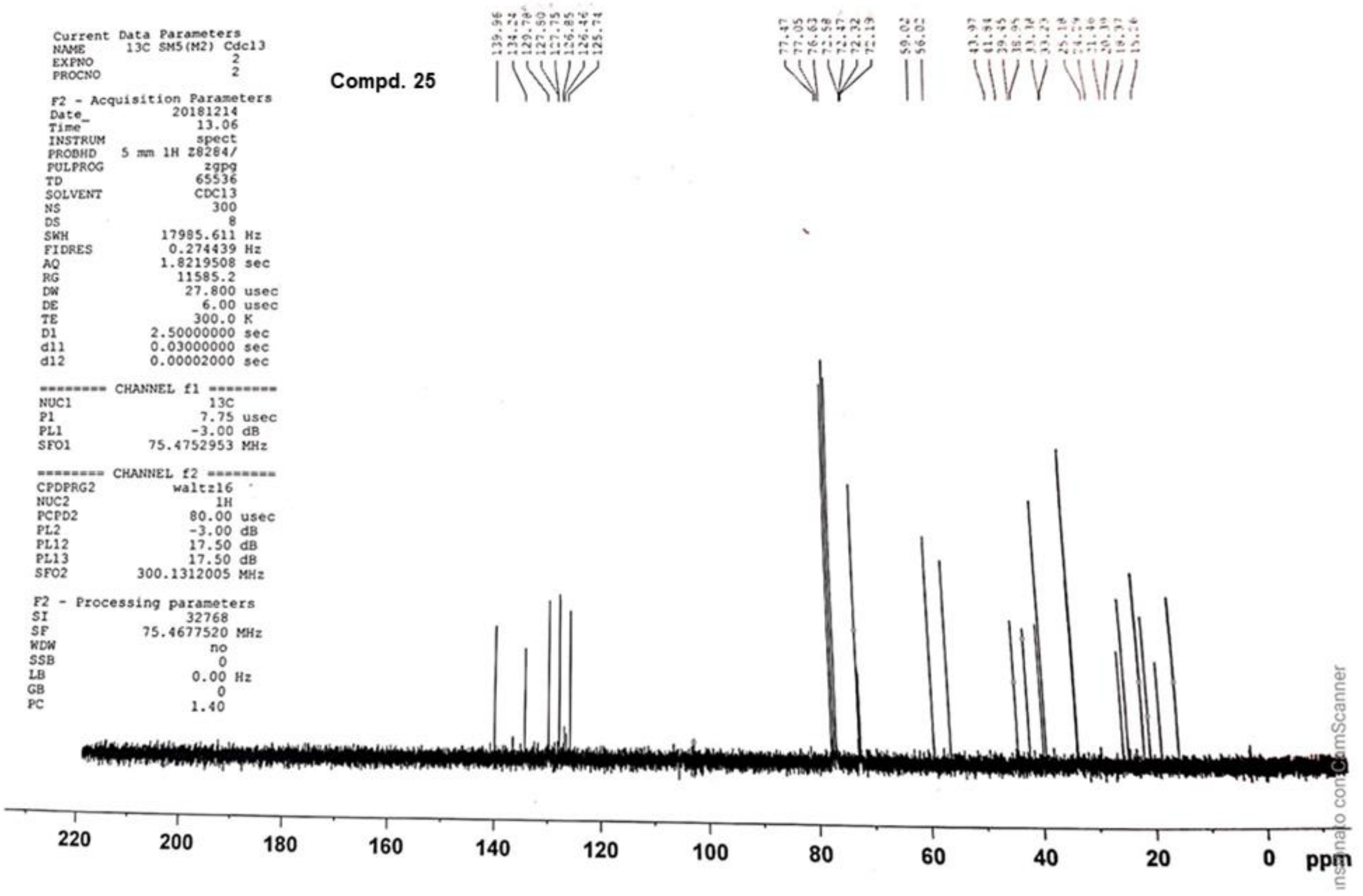
Current Data Parameters
NAME 13C SM5(M2) Cdcl3
EXPNO 2
PROCNO 2
F2 - Acquisition Parameters
Date_ 20181214
Time 13.06
INSTRUM spect
PROBHD 5 mm 1H Z8284/
PULPROG zgpg
TD 65536
SOLVENT CDCl3
NS 300
DS 8
SWH 17985.611 Hz
FIDRES 0.274439 Hz
AQ 1.8219508 sec
RG 11585.2
DW 27.800 usec
DE 6.00 usec
TE 300.0 K
D1 2.50000000 sec
d11 0.03000000 sec
d12 0.00002000 sec
======== CHANNEL f1 ========
NUC1 13C
P1 7.75 usec
PL1 -3.00 dB
SFO1 75.4752953 MHz
======== CHANNEL f2 ========
CPDPRG2 waltz16
NUC2 1H
PCPD2 80.00 usec
PL2 -3.00 dB
PL12 17.50 dB
PL13 17.50 dB
SFO2 300.1312005 MHz
F2 - Processing parameters
SI 32768
SF 75.4677520 MHz
WDW no
SSB 0
LB 0.00 Hz
GB 0
PC 1.40
139.96
134.24
129.78
127.80
127.75
126.85
126.46
125.74
77.47
77.05
76.63
72.58
72.47
72.32
72.19
59.02
56.02
43.97
41.94
39.45
38.95
33.38
33.23
25.18
24.79
21.46
20.39
18.37
15.16
220
200
180
160
140
120
100
80
60
40
20
0
ppm
Compd. 25
